# Highly energy-efficient information-handling dynamics of the brain

**DOI:** 10.1093/nsr/nwag373

**Published:** 2026-06-15

**Authors:** Jinxuan Ma, Wanlin Guo

**Affiliations:** State Key Laboratory of Mechanics and Control for Mechanical Structures, Nanjing University of Aeronautics and Astronautics, Nanjing 210016, China; State Key Laboratory of Mechanics and Control for Mechanical Structures, Nanjing University of Aeronautics and Astronautics, Nanjing 210016, China; Institute for Frontier Science, Nanjing University of Aeronautics and Astronautics, Nanjing 210016, China

**Keywords:** neural ensemble, information-handling dynamics, non-linear dynamics, Landauer’s principle

## Abstract

Artificial intelligence can outperform humans in specific tasks but at extremely high energy cost. Here we show that the brain can agglomerate neurons into a sphere to achieve energy-efficient, ultra-long-period electrophysiological activities. Chaos dynamics and fractal theory demonstrated the principle of the brain to memorize and process information through the formation of electrophysiological strange attractors under the global cooperation of neurons in the spatiotemporal structure. The neural sphere-based information-handling dynamics of the brain can then be constructed, predicting a storage capacity of 7.48 × 10^18^ bytes (3 orders higher than the previous estimates) and a computational power of 6.24 × 10^18^ floating-point operations per second (FLOPS) (∼78 000 modern GPUs) for the human brain with a surprising energy efficiency up to 79% via Landauer’s principle, 8 orders higher than that of the latest computer chips.

## Teaser

Information-handling dynamics reveals how neural collaboration generates diverse electrophysiological activities, offering a new explanation for the vast memory, powerful computation and exceptional energy efficiency of the brain.

## INTRODUCTION

People have long been committed to the exploration of the information mechanism of the brain to inspire the design of artificial intelligence (AI). In 2016, the groundbreaking emergence of AlphaGo [1] sparked a new AI storm that continues to this day. Relying on trillions of nanoscale field-effect transistors, AI systems operate at a frequency of several gigahertz, but at the cost of huge energy consumption. In comparison, the human brain consists of 86 billion micron-scale neurons that transmit information through action potential spikes in the order of milliseconds. It achieves remarkable cognitive feats with a mere 20 W of metabolic power [2]. However, the highly energy-efficient working mechanisms underlying the brain remain unclear. Based on the independence of neurons [3], the electrophysiological mechanisms [4–10] of neurodynamics can explain the signal transmission of action potentials in the brain. A variety of advanced imaging techniques [11–13] provide increasing data support for electrical signal transmission [14,15], neuronal morphology [16] and neural network structures [17–21]. The European Human Brain Project [22] has also made great efforts in this regard. Nevertheless, it remains a great challenge to understand how the brain memorizes and processes information from the huge information flow [23], heterogeneous networks [24,25] and highly non-linear dynamics of connectomics. Here we developed a neural sphere (NS) by optimizing the neural distribution, with the objective of minimum energy consumption to simulate the electrophysiological activities of the brain. Based on the fundamental principle and numerical simulations of generating different potential activities in the NS, we reveal the information-handling dynamics of the brain to explain the highly energy-efficient functionalities of the brain. Different from previous knowledge such as linear superposition based on synaptic polymorphism [23] or the all-or-nothing of action potentials, we found that the brain memorizes and processes information through the global cooperation of an NS in the spatiotemporal structure. The NS shows that the human brain can memorize and process information at 7.48 exabytes and 6.24 exaFLOPS, respectively. Combining Landauer’s principle with the above estimates, the power of the human brain for computing and communication is only 1.26 times the Landauer limit.

## NEURAL SPHERE FOR SIMULATING THE BRAIN

**Figure 1.**
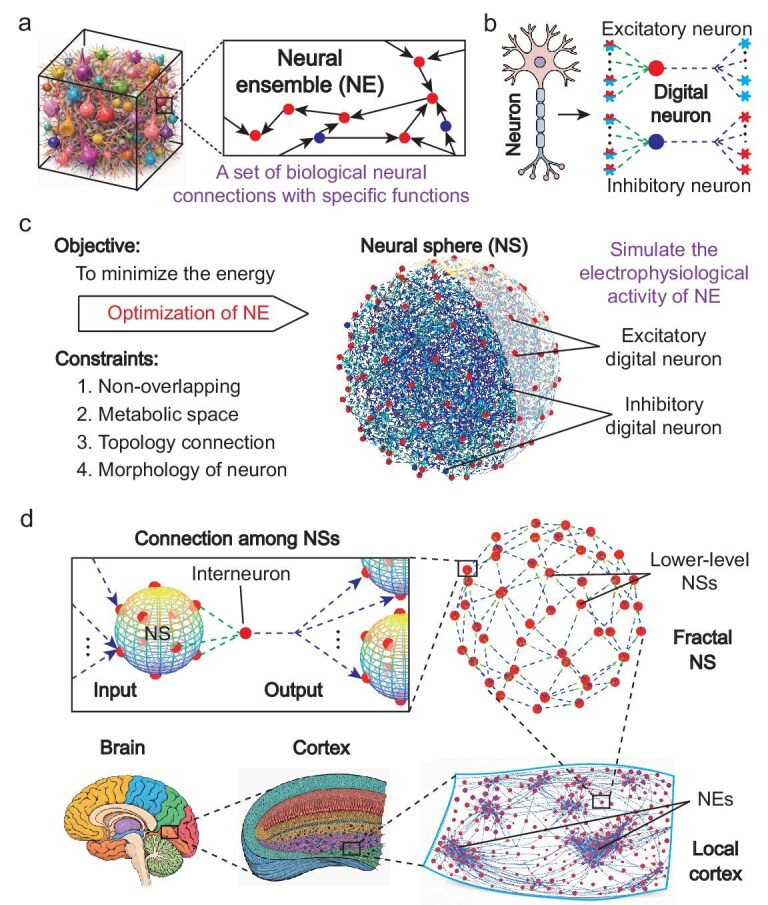
Schematic of the neural sphere (NS). (a) Schematic of the neural ensemble (NE). (b) Construction of digital neurons. The red dot indicates the soma of the excitatory neuron. The blue dot indicates the soma of the inhibitory neuron. The green dashed lines represent the dendrites. The blue dashed lines represent the axons. The cyan asterisks indicate the excitatory synapses. The red asterisks indicate the inhibitory synapses. (c) Optimization and construction of the NS. (d) Schematic of the large-scale connection among NSs in the cerebral cortex.

In the cerebral cortex, a large number of local neural connections can realize complex functions such as visual perception and emotional processing. The set of biological neural connections with specific functions is defined as the neural ensemble (NE) (Fig. 1a). To explore the information-handling dynamics of the brain, we develop an NS that unifies the morphological and functional features of the brain to simulate the electrophysiological activity of the NE. It includes the construction of digital neurons and the optimization of the distribution of neural connections. The detailed construction of the NS is presented in Methods S1.

In order to describe the microstructure and electrophysiological function [4–8,26], the biological neuron needs to be parameterized into the digital neuron by a group of orderly connected nodes (Fig. 1b). Each node includes the morphological information extracted by the neuron digital reconstruction technology [16] and the electrophysiological information. Depending on the neurotransmitter from the synaptic output, digital neurons are classified as excitatory digital neurons and inhibitory digital neurons. In the subsequent simulations, based on electrophysiological recordings from human hippocampal neurons [27], a general dynamical model (i.e. a variant of the Hodgkin–Huxley model [4–8,26] containing abundant ion channels) was used to fit the action potentials of digital neurons. The parameter selection of digital neurons is presented in Note S1.

Through the connection of digital neurons, the connection topologically equivalent to NE can be restored. Since the transmission of neural information is subject to strict metabolic constraints, the encoding and connectivity patterns of the brain networks exhibit significant energy efficiency optimization [28]. With the objective function of minimizing the energy consumption of NE, the optimal position distribution of neurons in the NE can be obtained by mathematical optimization (Fig. 1c). Its constraints include that neurons must not overlap, neurons must be within the maximum metabolic space, neural topological connections remain unchanged, and axons and dendrites of each neuron remain in a morphology with minimum energy consumption. By combining dynamic programming and genetic algorithms, a numerical solution with an average error of <0.2% can be obtained within a limited time budget. It is found that the sphere distribution of neuronal soma positions results in the NE of symmetric topology connection consume the least energy. In the NE of the general topology connection, the sphere distribution is expected to stably represent at least a suboptimal solution. Detailed optimization methods and results are presented in Methods S1.3, Note S2 and Movie S1. Based on this conclusion of optimization, a set of digital neurons with ordered connection and sphere distribution conforming to the minimum energy consumption criterion is defined as the NS. The somas are evenly distributed on the surface of the sphere following the Fibonacci grid [29], and the connections between digital neurons are distributed inside the sphere. However, other constraints besides energy can make the structure of the NE other than a perfect sphere, including irregular spatial constraints, different requirements for communication efficiency, robustness, plasticity, dynamical criticality etc. Using a standard NS can not only greatly simplify the complexity of model construction and simulation, but also effectively restore the electrophysiological activity characteristics of a small-scale NE. The implementation of simulation is presented in Methods S2.

Through the interconnection of a large number of small-scale NSs, the large-scale clusters of NSs can be constructed to simulate the electrophysiological activities of NEs with more complex functions or even the whole brain (Fig. 1d). The information transmission among NSs relies on the connections of a few interneurons. The interneuron integrates the electrophysiological activity of the NS and output to some neurons in other NSs. They are either independent or coupled in the NS. Besides this, an NE with more complex functions can be simulated by an NS with a fractal structure. Every Fibonacci grid node in the fractal NS could be a lower-level NS. At the macroscopic cortical scale, a topologically equivalent small-world network [30] can be constructed on the basis of mostly local clustered connections and a few connections made over longer distances among NSs, thereby enabling the simulation of large-scale electrophysiological activity in the local brain. Moreover, real biological neural systems are formed through multi-party tradeoffs and long-term evolution. In order to save energy and space as much as possible, and adapt to complex functional requirements, some distributions of neurons are a large number of nesting and stacking of incomplete near-NS structures, including the fold on the cortical surface, the cortical column, the local spherical surface, the multi-layered spoon-shaped stack, the tubular distribution and so on. These fragmented near-NS structures can be initially isolated from some morphological data [27] (Notes S2–S3).

## ELECTROPHYSIOLOGICAL ACTIVITY OF AN NS

Through a series of systematic simulations of small-scale NSs with different connection probability *p* and initial condition, it has been found that an NS will exhibit four typical electrophysiological activities. It includes briefly triggering a few action potentials, sustained short-period action potential activity, sustained long-period action potential activity and sustained random potential activity. Comprehensive data of electrophysiological activities for small-scale NSs are presented in Note S4.

**Figure 2.**
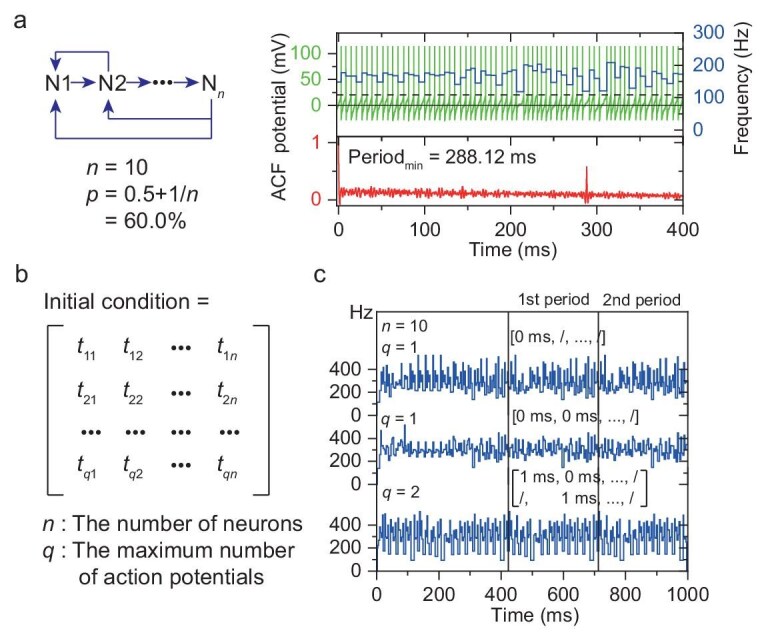
The electrophysiological activity of an NS. (a) Full positive feedback-connected NS. (Left) Synaptic connection schematic diagram. N*_i_* is the digital neuron with index *i*. (Right) The solid green line is the membrane potential time series of one of the digital neurons. The black dashed line is the threshold potential, 20 mV. The blue solid line is the real-time frequency spectra of action potential as a function of time. The solid red line represents the autocorrelation coefficient function, ACF [31]. (b) Matrix representation of the initial conditions of the NS. (c) Real-time frequency of triggered action potentials for one of the neurons of a full positive feedback-connected NS in three initial conditions.

Taking the NS of 10 digital neurons as an example, Fig. 2a shows the membrane potential activities of one of the neurons in the full positive feedback-connected NS. According to the first peak of the autocorrelation coefficient function of the membrane potential activity time series [31], it is found that action potential activity has an ultra-long period, 288.11 ms, which is on the same level as the average human reaction time (200–300 ms) [32]. The average action potential frequency is 167 Hz, belonging to fast gamma waves (80–200 Hz) [15]. A large amount of ordered information is able to be stored in the NE through periodic changes in action potential activity to realize the memory and learning functions of the brain.

Based on comprehensive numerical experiments, it is found that most of the NSs within 40% < *p* < 70% have ultra-long period (T > 100 ms) electrophysiological activity. The set of periodic membrane potential activity of all neurons in the NS is defined as the periodic potential waveform. These periodic potential waveforms are significantly affected by the initial conditions. The initial condition for the NS with *n* neurons can be represented as a matrix with *q* rows and *n* columns (Fig. 2b). *q* represents the maximum number of action potentials triggered. *t_ji_* represents neuron *i* triggering *j*th action potential at moment *t_ji_. t_ji_* = / represents no action potential triggered. In the examples of Fig. 2c, different initial conditions will converge to different periodic potential waveform through the NS.

**Figure 3.**
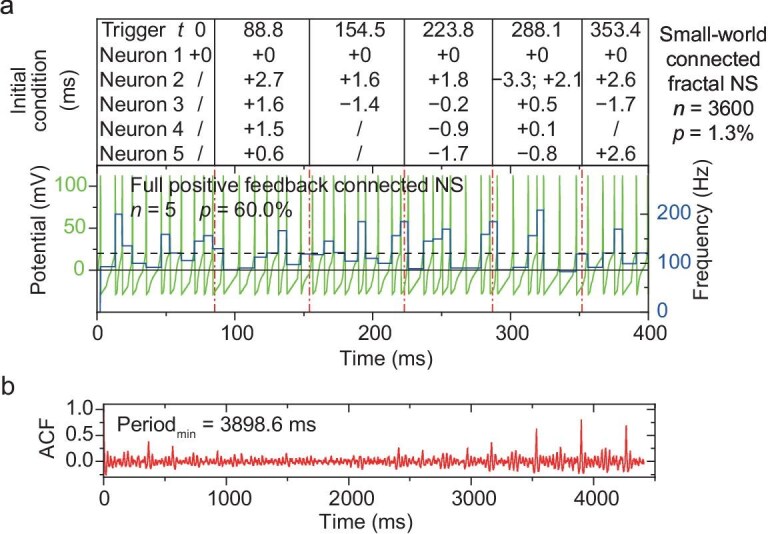
Electrophysiological simulation of the small-scale NS in large-scale fractal NS. The fractal NS is composed of 600 5-neuron full positive feedback-connected NSs and 600 interneurons via the small-world network connection. (a) One of the electrophysiological activities of the 5-neuron NSs. (Upper) The firing times at which the NS receives inputs from the other NSs, and the initial conditions correspond to each waveform segment. (Lower) The solid green line is the membrane potential time series of one of the digital neurons. The blue solid line is the real-time frequency spectra of action potential as a function of time. The red lines represent the trigger time when the NS receives a new input. (b) The autocorrelation coefficient function (ACF) of the above 5-neuron NS.

Furthermore, the electrophysiological activities of large-scale NSs also have long-period characteristics. A symmetrical fractal NS with 3600 neurons is used for simulation. It has 600 Fibonacci grid nodes. The nodes are connected via the small-world network with a connection probability of 1.6%. Each node is a 5-neuron full positive feedback-connected NS and an interneuron responsible for integrating the output. In each 5-neuron NS, only neuron 1 is used to receive several inputs from other interneurons. The initial condition for the simulation of the fractal NS is that neuron 1 in each 5-neuron NS triggers an action potential at 0 ms. Figure 3 shows the electrophysiological activity of one of the neurons in a 5-neuron NS. At each red line in Fig. 3a, this 5-neuron NS receives inputs from other interneurons in turn. The potential waveform between two adjacent inputs is equivalent to a part of the potential waveform in an independent 5-neuron NS with a specific initial condition. Through the concatenation of potential waveforms of the independent NS with different initial conditions, the electrophysiological activity of this neuron in the 5-neuron NS will exhibit an ultra-long period of up to 3898.6 ms (Fig. 3b). Besides this, a fractal NS of 15 600 neurons with reduced symmetry is constructed and simulated to further illustrate that the large-scale NS exhibits similar electrophysiological activity characteristics to the small-scale NS, with only differences in the time scale. Detailed simulation data are presented in Note S5.

In the actual brain, different-scale NSs are coupled with each other and continuously receive external inputs to update the electrophysiological activities. Based on the above numerical experiments, the electrophysiological activity of a neuron in the brain is the permutation of a set of potential waveforms corresponding to different initial conditions on a time series. Also, some macroscopic biological behaviors can verify the rationality of periodic electrophysiological activities, including the rhythm of the brain, the regular activity of organs, the sleep cycle, the biological clock and so on.

## INFORMATION FEATURES OF AN NS

**Figure 4.**
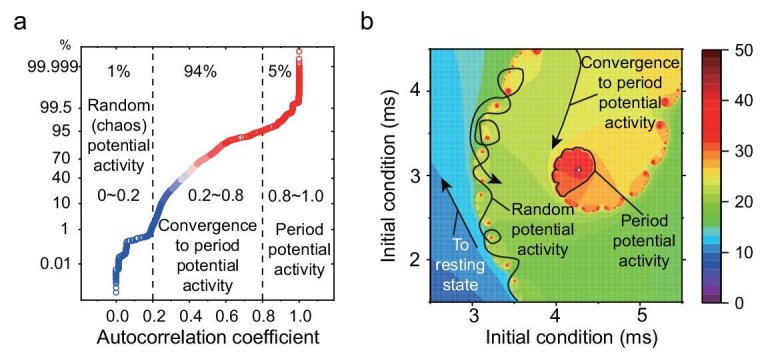
Electrophysiological simulation of an NS and hysteresis complex iteration theory of a simplified NS dynamical system. (a) The cumulative probability curve of the autocorrelation coefficient of all neuronal potential activities under all initial conditions. (b) One of the 2D local projections of the phase portrait for a simplified NS dynamical system. The horizontal and vertical coordinates respectively correspond to the initial conditions of the two neurons in the NS. The color graph represents the number of iterations.

Corresponding to all sets of the initial condition matrix, the universal set of potential activities describes the information features of NSs. Combining electrophysiological simulation with chaos theory of the non-linear NS dynamical system (Fig. 4), the fundamental principle of the different potential activities exhibited by NSs can be well revealed.

In order to characterize the information features of NSs, 221 551 groups of electrophysiological activities under all initial conditions of the full positive feedback NS with *n* = 5 and *q* = 1 are simulated. These initial conditions cover all situations that guarantee that the NS generates sustained potential activity. The autocorrelation coefficient of the membrane potential activity time series close to 1 or 0 indicates that the potential activity has stronger periodicity or chaotic property, respectively. According to the cumulative probability curve in Fig. 4a, most of the potential activity (94%) will converge to periodic potential activity. Only a few potential activities are random (1%) or periodic (5%). Comprehensive simulation data are presented in Note S6.

To explain the above mathematical principle of the information features of NSs, an NS dynamical system is constructed to analyze how the initial conditions drive the potential activity of NSs. The information interaction between neurons is a typical non-linear process. The non-linear features of the NS information system originate from the synaptic integration of neurons, the all-or-nothing action potential output property, the hysteresis between input and output, and the complexity of network connections. In order to simulate NS dynamical systems in acceptable time, it is necessary to simplify the system without changing the source of non-linearity, including reducing the action potential waveform to a spike functional form, incorporating some linear parameters, and transforming the control parameters. In a simplified NS dynamical system, the state of each neuron is described by a complex number with the real part representing the state of action potential and the imaginary part representing the initial condition *t_i_*. Each complex iteration of the dynamical system is equivalent to the synaptic integration of each neuron for several hysteresis inputs from the remaining connected neurons. Taking the initial state of each neuron as a dimension, the NS dynamical system will constitute an *n*-dimensional complex space. In the full positive feedback NS dynamical system with *n* = 5 and *q* = 1, staining one of the planar projections of the high-dimensional complex space according to the number of iterations, a phase portrait with a beautiful fractal structure and a large number of strange attractors can be obtained (Fig. 4b). The periodic potential waveform is a high-dimensional strange attractor in space. The point of convergence to the attractor corresponds to convergence to periodic potential activity. All points flowing on the high-dimensional attractor correspond to a group of specific initial condition matrixes. Being randomly and alternately attracted by several attractors corresponds to random potential activity. The proportion of area in the phase portrait of the above three cases is well consistent with the simulated data in Fig. 4a. The trajectories of the different types of electrophysiological activities are all represented by arrows in Fig. 4b. Detailed theoretical modeling of the dynamical system is presented in Methods S3, Note S7 and Movie S2.

## INFORMATION HANDLING DYNAMICS OF THE BRAIN

**Figure 5.**
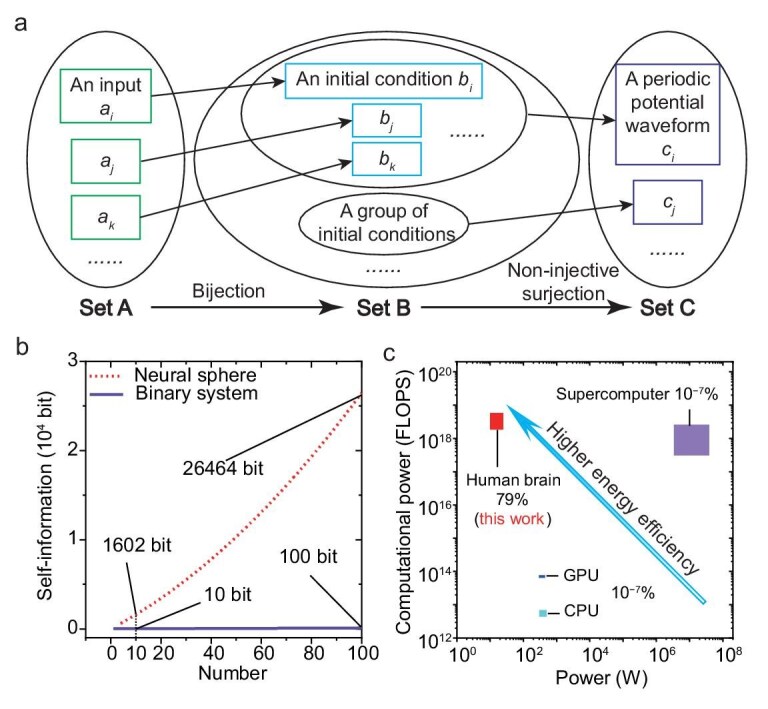
Evaluation of storage capacity and computational efficiency for neural sphere and binary system. (a) Schematic of NS memorizing and processing information. (b) Curves of the self-information of NS and binary system as a function of the number of neurons or devices. (c) Comparison of computational efficiency, power and energy efficiency between the human brain and silicon-based devices. The data of each device are presented in Note S9. The computing power is measured by double-precision floating-point operations per second (FLOPS). The respective energy efficiency is annotated in the figure.

According to the information features of the NS, the information-handling dynamics of the brain is revealed to describe how the brain organizes, memorizes and processes information. A large number of NEs in the brain can be abstracted as NSs with long-period potential activity. Through the regulation of short-term synaptic plasticity from a group of neurons, the input to an NS is a group of action potentials that trigger sequentially at specific moments. It is equivalent to the group of membrane potential states of all neurons at a particular instant in the periodic potential waveform. Through the bijection from the input information outside the NS to the initial conditions (Set A to Set B in Fig. 5a) and the non-injective surjection from the initial conditions to the periodic potential waveforms (Set B to Set C), the input information can be stored in the NS (Set A to Set C). Through the integration of small-scale NSs with different structures and functions, the biological information flow will be iteratively memorized and processed across different NSs. Methods S4 further describes the working mechanism of NS for organizing information.

In order to quantify the advantages of the information handling of the brain, it is necessary to calculate its storage capacity and computational power through the principles of Shannon information theory [33] and floating-point operations per second (FLOPS), respectively. When triggering a certain periodic potential waveform in an NS with an initial condition, a piece of information stored in the waveform can be repeatedly extracted. By counting the number of periodic potential waveforms and the amount of information that can be processed in a period, the storage and computing capacity of an NS can be estimated, respectively. At the mathematical limit (Fig. 4b), an NS theoretically has an infinite number of periodic potential waveforms. Limiting the initial condition matrix by limiting the temporal resolution is able to further limit the number of periodic potential waveforms.

By calculating the self-information of the binary system and the NS (Methods S5), the curves of the self-information as a function of the number of devices or neurons are obtained and shown in Fig. 5b. The binary system of current computer architecture has low self-information. When the time resolution is the physical limit (Planck time, 5.39 × 10^−44^ s), the self-information of an NS is three or more orders of magnitude larger than that of the binary system. The storage capacity of the human brain can also be simply estimated via an NS. Assuming that the human brain has 86 billion neurons, each neuron has about 5000 synapses. The connection structure of neurons in the human brain is a small-world network composed of a large number of fractal NSs, as shown in Fig. 1d. The maximum estimate of the storage capacity for the human brain modeled by the NS is then 7.48 × 10^18^ bytes. It is much larger than the previous maximum estimate of 1 × 10^15^ bytes [23]. Note S8 further presents the self-information of NSs in different conditions and 2D material devices [34–36].

Although an NS is not well-suited for floating-point operations, the integrated output of a single action potential or the memory space operable per unit time can be equivalent to several floating-point operations to measure its computational power (Methods S6). Through the above two equivalent methods, the maximum computational power of the human brain modeled by an NS is estimated to be 1.72 × 10^18^ and 6.24 × 10^18^ FLOPS (Fig. 5c). Its computational power is slightly higher than that of the supercomputer, equivalent to 78 000 latest-generation GPUs.

**Figure 6.**
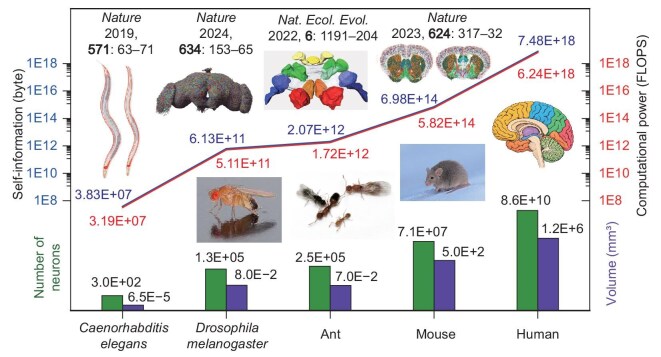
Evaluation of the maximum storage capacity and computational power for *Caenorhabditis elegans* [37], *Drosophila melanogaster* [18], ant [38], mouse [39] and human by an NS. The storage capacity is measured by self-information. Computational power is measured by double-precision floating-point operations per second (FLOPS). In the bar graph, green represents the number of neurons and purple represents the volume of the brain.

Due to the universality of NSs, the storage capacity and computational power of the brain of other organisms can be estimated as shown in Fig. 6. The brain of the same organism has the same order of magnitude of storage capacity and computational power. In the brains of *Caenorhabditis elegans* [37], *Drosophila melanogaster* [18], ant [38], mouse [39] and human, the human brain has an absolute lead, beating the mouse by 4 orders of magnitude.

Besides this, based on Landauer’s principle [40,41], the physical limit of the power for NSs can be analyzed. Once a new set of inputs is received, the NS will generate a new segment of potential waveform, which is equivalent to erasing the information of the original waveform in the physical statistics system of the NS by generating new information. This information processing process will inevitably produce a minimum energy, the Landauer limit. According to the maximum estimate in Fig. 5, the Landauer limit of the human brain modeled by an NS is about 2.37 W. This estimation is merely a rough reference for assessing the ideal physical limit. The calculation method of this limit is presented in Methods S7. Based on the existing experimental data, the power of the human brain for computing and communication is about 3 W [42], which is only 1.26 times the Landauer limit. In other words, its energy efficiency has reached 79% (Fig. 5c). However, modern computers are a billion times beyond their Landauer limit, far less energy-efficient than biological systems such as the human brain. Even the most advanced chips at present have an energy efficiency of only about 1 × 10^−7^%. It means that the energy efficiency of the human brain has almost reached its limit after a long period of evolution. It also verifies the rationality of NSs in the estimated storage capacity and computing power.

## DISCUSSION

In summary, this work reconstructed the electrophysiological activity by developing an energy-optimal NS to reveal possible information-handling dynamics of the brain. Existing theories based on binary or mixed-radix logic simply assume that the brain memorizes information linearly in synapses or neurons [23]. According to the theoretical derivation and simulation results of NSs, we found that these information-handling dynamics can non-linearly memorize information in periodic potential waveforms that depend on both the temporal dimension and the spatial structure of neurons. By integrating memories from these waveforms to accumulate experience, the NS could generate reflex, cognition and creativity (Note S10). In other words, the NS can maximally exploit the spatiotemporal dimension to establish clustering effects among neurons or NEs, enabling the storage of vast amounts of information while consuming a small amount of neuronal energy. However, the microphysical mechanism of the brain to achieve such extremely low-energy information handling still needs further exploration.

Furthermore, although NSs can well explain the operating mechanism of brain intelligence and most biological behaviors, the realization of biological intelligence still requires the verification of more comprehensive experiments and in-depth theories. In revealing the complex structure and function of the NE or even the whole brain, the NS system still needs more improvement. In the anatomical structure of the NE, the NS only considers the skeleton structures of the neuron. More microscopic organelle distributions and more detailed 3D structures of the neuron will make the results more accurate. In the connection network of the NE, this work only considers several common connection patterns to obtain the overall neural activity characteristics of NSs. In fact, the personalized adjustment of the connection matrix and the parameters of each neuron can precisely control the variation of all potential waveforms. However, due to the huge computational cost and the black-box structure of the neural network, the simulation of such precise control is difficult to achieve currently. In restoring the complex functions of the NE, the NS only takes into account the electrophysiological activities of normal neurons under sufficient energy supply. In biological neurons, the complex chemical activities and other unclear mechanical mechanisms can also have limited impact on the simulation results.

To sum up, we hope that this article will serve as an important reference for explaining the efficient working principle of the brain to facilitate detailed investigation of information mechanisms in biological intelligence, and provide a possible path to achieving artificial general intelligence.

## Supplementary Material

nwag373_Supplemental_Files

## Data Availability

The neuron morphology data were obtained from Digitally Reconstructed Neurons and Glia, https://neuromorpho.org/. The data of the latest generation high-performance CPU and GPU and the top 10 supercomputer were obtained from various institutions or manufacturers, including https://www.intel.com/content/www/us/en/ark.html#@Processors, https://www.amd.com/en/products/processors/server/epyc/9005-series.html, https://www.amd.com/en/products/graphics/workstations.html, https://www.nvidia.com/en-us/geforce/graphics-cards/40-series/rtx-4090/ and https://www.top500.org/lists/top500/2024/11/. Other datasets collected and/or analyzed during the current study are available from the corresponding author upon request.

## References

[1] Silver D, Huang A, Maddison CJ et al. Mastering the game of Go with deep neural networks and tree search. Nature 2016; 529: 484–9. 10.1038/nature16961 26819042

[2] Howarth C, Gleeson P, Attwell D. Updated energy budgets for neural computation in the neocortex and cerebellum. J Cereb Blood Flow Metab 2012; 32: 1222–32. 10.1038/jcbfm.2012.35 22434069 PMC3390818

[3] Cajal S . Estructura de los centros nerviosos de las aves (in Spain). Rev Trim Histol Norm Patol 1888; 1: 1–10.

[4] Hodgkin AL, Huxley AF, Katz B. Measurement of current-voltage relations in the membrane of the giant axon of *Loligo*. J Physiol 1952; 116: 24–48. 10.1113/jphysiol.1952.sp004717 PMC1392219 14946712

[5] Hodgkin AL, Huxley AF. Currents carried by sodium and potassium ions through the membrane of the giant axon of *Loligo*. J Physiol 1952; 116: 449–72. 10.1113/jphysiol.1952.sp004717 14946713 PMC1392213

[6] Hodgkin AL, Huxley AF. The components of membrane conductance in the giant axon of *Loligo*. J Physiol 1952; 116: 473–96. 10.1113/jphysiol.1952.sp004718 14946714 PMC1392209

[7] Hodgkin AL, Huxley AF. The dual effect of membrane potential on sodium conductance in the giant axon of *Loligo*. J Physiol 1952; 116: 497–506. 10.1113/jphysiol.1952.sp004719 14946715 PMC1392212

[8] Hodgkin AL, Huxley AF. A quantitative description of membrane current and its application to conduction and excitation in nerve. J Physiol 1952; 116: 500–44. 10.1113/jphysiol.1952.sp004764 PMC1392413 12991237

[9] Traub RD, Wong RK, Miles R et al. A model of a CA3 hippocampal pyramidal neuron incorporating voltage-clamp data on intrinsic conductances. J Neurophysiol 1991; 66: 635–50. 10.1152/jn.1991.66.2.635 1663538

[10] Wang XJ, Buzsáki G. Gamma oscillation by synaptic inhibition in a hippocampal interneuronal network model. J Neurosci 1996; 16: 6402–13. 10.1523/JNEUROSCI.16-20-06402.1996 8815919 PMC6578902

[11] Basser PJ, Mattiello J, LeBihan D. MRI of neuronal network structure, function, and plasticity. Biophysical J 1994; 66: 259–67. 10.1016/S0006-3495(94)80775-1 PMC1275686 8130344

[12] Steinmetz NA, Aydin C, Lebedeva A et al. Neuropixels 2.0: a miniaturized high-density probe for stable, long-term brain recordings. Science 2021; 372: eabf4588. 10.1126/science.abf4588 33859006 PMC8244810

[13] Sofroniew NJ, Flickinger D, King J et al. A large field of view two-photon mesoscope with subcellular resolution for in vivo imaging. eLife 2016; 5: e14472. 10.7554/eLife.14472 27300105 PMC4951199

[14] Stringer C, Pachitariu M. Analysis methods for large-scale neuronal recordings. Science 2024; 386: eadp7429. 10.1126/science.adp7429 39509504

[15] Buzsáki G, Draguhn A. Neuronal oscillations in cortical networks. Science 2004; 304: 1926–9. 10.1126/science.1099745 15218136

[16] Ascoli GA, Donohue DE, Halavi M. NeuroMorpho.Org: a central resource for neuronal morphologies. J Neurosci 2007; 27: 9247–51. 10.1523/JNEUROSCI.2055-07.2007 17728438 PMC6673130

[17] White JG, Southgate E, Thomson JN et al. The structure of the nervous system of the nematode *Caenorhabditis elegans*. Philos Trans R Soc B 1986; 314: 1–340. 10.1098/rstb.1986.0056 22462104

[18] Lin A, Yang R, Dorkenwald S et al. Network statistics of the whole-brain connectome of *Drosophila*. Nature 2024; 634: 153–65. 10.1038/s41586-024-07968-y 39358527 PMC11446825

[19] Turner NL, Macrina T, Bae JA et al. Reconstruction of neocortex: organelles, compartments, cells, circuits, and activity. Cell 2022; 185: 1082–100. 10.1016/j.cell.2022.01.023 35216674 PMC9337909

[20] Gamlin CR, Schneider-Mizell CM, Mallory M et al. Connectomics of predicted *Sst* transcriptomic types in mouse visual cortex. Nature 2025; 640: 497–505. 10.1038/s41586-025-08805-6 40205210 PMC11981948

[21] Sporns O, Tononi G, Kötter R. The human connectome: a structural description of the human brain. PLoS Comput Biol 2005; 1: e42. 10.1371/journal.pcbi.0010042 16201007 PMC1239902

[22] Naddaf M . Europe spent €600 million to recreate the human brain in a computer. How did it go? Nature 2023; 620: 718–20. 10.1038/d41586-023-02600-x

[23] Bartol TM Jr, Bromer C, Kinney J et al. Nanoconnectomic upper bound on the variability of synaptic plasticity. eLife 2015; 4: e10778. 10.7554/eLife.10778 26618907 PMC4737657

[24] Sporns O, Kötter R. Motifs in brain networks. PLOS Biol 2004; 2: e369. 10.1371/journal.pbio.0020369 15510229 PMC524253

[25] Luo L . Architectures of neuronal circuits. Science 2021; 373: eabg7285. 10.1126/science.abg7285 34516844 PMC8916593

[26] Rall W . Branching dendritic trees and motoneuron membrane resistivity. Exp Neurol 1959; 1: 491–527. 10.1016/0014-4886(59)90046-9 14435979

[27] Ding SL, Royall JJ, Sunkin SM. Comprehensive cellular-resolution atlas of the adult human brain. J Comp Neurol 2016; 524: 3127–481. 10.1002/cne.24080 27418273 PMC5054943

[28] Laughlin SB, Sejnowski TJ. Communication in neuronal networks. Science 2003; 301: 1870–4. 10.1126/science.1089662 14512617 PMC2930149

[29] Gonzalez A . Measurement of areas on a sphere using Fibonacci and latitude–longitude lattices. Math Geosci 2010; 42: 49–64. 10.1007/s11004-009-9257-x

[30] Sporns O, Honey CJ. Small worlds inside big brains. Proc Natl Acad Sci USA 2006; 103: 19219–20. 10.1073/pnas.0609523103 17159140 PMC1748207

[31] Park KI, Park M. Parameters of a Stochastic Process. Fundamentals of Probability and Stochastic Processes with Applications to Communications. Cham: Springer, 2018, 165–8.

[32] Kosinski RJ . A literature review on reaction time. Clemson Univ 2008; 10: 337–44.

[33] Shannon CE . A mathematical theory of communication. Bell Syst Tech J 1948; 27: 379–423. 10.1002/j.1538-7305.1948.tb01338.x

[34] Zheng Z, Ma Q, Bi Z et al. Unconventional ferroelectricity in moiré heterostructures. Nature 2020; 588: 71–6. 10.1038/s41586-020-2970-9 33230334

[35] Yasuda XWK, Watanabe K, Taniguchi T et al. Stacking-engineered ferroelectricity in bilayer boron nitride. Science 2021; 372: 1458–62. 10.1126/science.abd3230 34045323

[36] Sharma D, Rath SP, Kundu B et al. Linear symmetric self-selecting 14-bit kinetic molecular memristors. Nature 2024; 633: 560–6. 10.1038/s41586-024-07902-2 39261726

[37] Cook SJ, Jarrell TA, Brittin CA et al. Whole-animal connectomes of both *Caenorhabditis elegans* sexes. Nature 2019; 571: 63–71. 10.1038/s41586-019-1352-7 31270481 PMC6889226

[38] Li Q, Wang M, Zhang P et al. A single-cell transcriptomic atlas tracking the neural basis of division of labour in an ant superorganism. Nat Ecol Evol 2022; 6: 1191–204. 10.1038/s41559-022-01784-1 35711063 PMC9349048

[39] Yao Z, Velthoven CTJ, Kunst M et al. A high-resolution transcriptomic and spatial atlas of cell types in the whole mouse brain. Nature 2023; 624: 317–32. 10.1038/s41586-023-06812-z 38092916 PMC10719114

[40] Bérut A, Arakelyan A, Petrosyan A et al. Experimental verification of Landauer’s principle linking information and thermodynamics. Nature 2012; 483: 187–9. 10.1038/nature10872 22398556

[41] Jun Y, Gavrilov M, Bechhoefer J. High-precision test of Landauer’s principle in a feedback trap. Phys Rev Lett 2014; 113: 190601. 10.1103/PhysRevLett.113.190601 25415891

[42] Lennie P . The cost of cortical computation. Curr Biol 2003; 13: 493–7. 10.1016/S0960-9822(03)00135-0 12646132

