# Supplementary material for: Highly energy-efficient information-handling dynamics of the brain
Source: Natl Sci Rev. 2026 Jun 15;13(15):nwag373. doi: 10.1093/nsr/nwag373 (PMC13455624; doi:10.1093/nsr/nwag373)
Supplement: Supplementary file 1 [file nwag373_supplemental_files.zip › Supplemental data.pdf]

Supplemental Material for

Highly energy efficient information handling dynamics of brain

Jinxuan Ma (马锦晅, 0009-0007-5172-1640)[1], Wanlin Guo (郭万林, 0000-0002-2302-8044)[1,2,*]

[1]State Key Laboratory of Mechanics and Control for Aerospace Structures, Nanjing University of Aeronautics and Astronautics, Nanjing 210016, China
[2]Institute for Frontier Science of Nanjing University of Aeronautics and Astronautics, Nanjing University of Aeronautics and Astronautics, Nanjing 210016, China.

Corresponding author: Wanlin Guo, wlguo@nuaa.edu.cn

The Supplementary information for this manuscript includes the following:

Methods S1 to S7
Notes S1 to S10
Figs. S1 to S41
Tables S1 to S5
Movies S1 to S2
Data S1
References

Methods

1. Construction of the neural sphere (NS)

This section provides the detailed method for the construction of the neural sphere (NS), including the construction of digital neuron to describe the structure and function at the microcosmic scale (Fig. S1 and Fig. S2a-g), the optimization of the sphere distribution for the neural ensemble (NE) (Fig. S2i-j), and the sphere connection structure to describe the synaptic connections of neural networks at the macroscopic scale (Fig. S2h).

1.1 Digital neuron

When simulating the membrane potential activity, the design of a single neuron model is essential. The digital neuron is constructed to describe the characteristics of neurons and applies it to neural network simulations (Fig. S1). The digital neuron takes into account both the functionality and morphology of individual neurons, enabling efficient simulation of interactions between neurons. Figure S2a is an example of NMO_00927 excitatory neuron from Digitally Reconstructed Neurons and Glia [1] plotted through digital neuron.

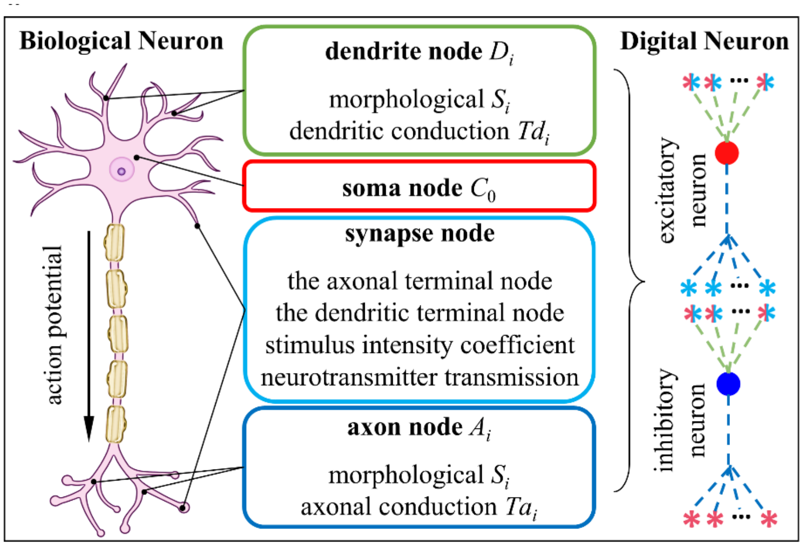

Fig. S1. Schematic of the digital neuron. Construction of the digital neuron. It consists of four types of nodes, including dendrite node, soma node, axon node and synapse node. The right side of this subfigure shows the schematic representation of the excitatory and inhibitory digital neurons. The red dot indicates the soma of excitatory neuron. The blue dot indicates the soma of inhibitory neuron. The green dashed lines represent the dendrites. The dashed blue lines represent the axons. The cyan asterisks indicate the excitatory synapses. The red asterisks indicate the inhibitory synapses.

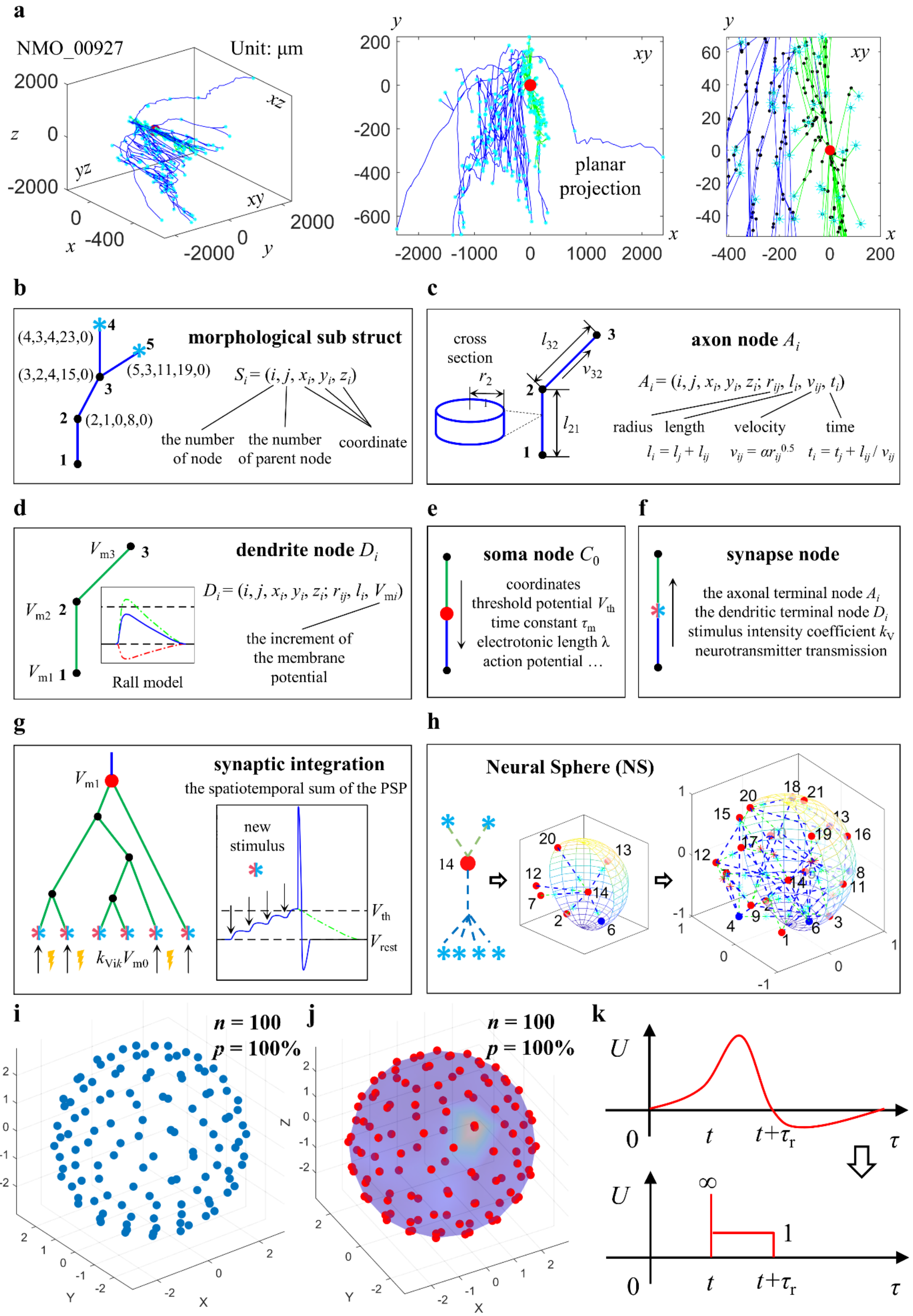
a
NMO_00927
Unit: μm
planar projection
b
morphological sub struct
$S_i = (i, j, x_i, y_i, z_i)$
the number of node
the number of parent node
coordinate
(4,3,4,23,0)
(3,2,4,15,0)
(5,3,11,19,0)
(2,1,0,8,0)
c
cross section
axon node A_i
$A_i = (i, j, x_i, y_i, z_i; r_{ij}, l_i, v_{ij}, t_i)$
radius
length
velocity
time
$l_i = l_j + l_{ij}$
$v_{ij} = \alpha r_{ij}^{0.5}$
$t_i = t_j + l_{ij} / v_{ij}$
d
dendrite node D_i
$D_i = (i, j, x_i, y_i, z_i; r_{ij}, l_i, V_{mi})$
the increment of the membrane potential
Rall model
e
soma node C_0
coordinates
threshold potential V_{th}
time constant τ_m
electrotonic length λ
action potential …
f
synapse node
the axonal terminal node A_i
the dendritic terminal node D_i
stimulus intensity coefficient k_V
neurotransmitter transmission
g
synaptic integration
the spatiotemporal sum of the PSP
new stimulus
V_{m1}
$k_{Vik} V_{m0}$
V_{th}
V_{rest}
h
Neural Sphere (NS)
i
$n = 100$
$p = 100\%$
j
$n = 100$
$p = 100\%$
k
U
0
t
$t+\tau_r$
τ
∞
1

Fig. S2. The supplementary schematic diagram of the NS. (**b**-**g**) complement the composition of digital neuron. (**a**) Example of NMO_00927 excitatory neuron plotted through digital neuron. Morphological data for this neuron are obtained from Digitally Reconstructed Neurons and Glia[1]. The blue lines represent the axon. The green lines represent the dendrites. The cyan asterisks indicate the synapse. The red dot indicates the soma. (left) Three-dimensional view of the NMO_00927 neuron. (middle) Projection of the NMO_00927 neuron on the *xy*-plane. (right) Local magnification near the soma. The black dots represent the nodes. (**b**) The schematic diagram of the morphological sub struct. (**c**) The schematic diagram of the axon node A_i. (**d**) The schematic diagram of the dendrite node D_i. It uses the Rall model [2] to describe the membrane potential on the dendrites. Green dotted line, red dotted line and solid blue line represent excitatory, inhibitory and sum postsynaptic potential (PSP), respectively. (**e**) The schematic diagram of the soma node C_0. The arrow indicates the direction of conduction. (**f**) The schematic diagram of the synapse node. (**g**) The schematic diagram of the synaptic integration. During synaptic continuously receives the stimulation, neuron determines whether to trigger an action potential by monitoring the spatiotemporal sum of the PSP. (**h**) The composition of the NS. It is constructed from projections and connections of digital neuron in the unit sphere. An example of the connection of neuron 14 in the sphere is presented here. (**i**) Optimal distribution of spatial coordinates of neurons with n = 100, p = 100%. The blue scatter is soma. (**j**) Sphere structure of the coordinate distribution for the NE. The detailed visualization of the optimization process is presented in Movie S1. (**k**) Simplified schematic representation of the action potential for the neuron.

The dendrites and axons of multi-level neurons often exhibit numerous branching structures, forming intricate spatial trees. Neurons can be equivalently represented as a collection of branching or turning points within the spatial trees of dendrites and axons

$$N = \{C_0, D_i, A_j\},\ i = -1, -2,\ \dots, -n_{\mathrm{d}}, j = 1, 2,\ \dots, n_{\mathrm{a}}, \tag{1}$$

where C_0 represents the soma node, D_i represents the node $-i$ on the dendrite (the numbering of dendritic node takes negative values), A_j represents the node j on the axon (the numbering of axonal node takes positive values), n_{d} represents the number of nodes on the dendrite, and n_{a} represents the number of nodes on the axon. The terminal nodes of the axon and dendrite are synapse nodes. Each node is a struct that describes the characteristics of neurons at the current location. The following will provide a detailed explanation of how different types of nodes represent the morphology and functionality of neurons, in order the morphological sub struct, axon node, dendrite node, soma node and synapse node.

The morphological sub structs are included in all nodes, describing the position and connection of the current node (Fig. S2b)

$$S_i = (i, j, x_i, y_i, z_i), \tag{2}$$

where i is the number of the node, j is the number of the parent node ($|j| < |i|$), and (x_i, y_i, z_i) is the coordinate of the node. In particular, the soma node is represented by (0, -1, 0, 0, 0), and the axon hillock node is represented by (1, 0, 0, 0, 0).

The function of the axon node is to transmit information by action potentials (Fig. S2c). Once an action potential is triggered, it maintains a consistent amplitude as it conducts along the axon. The conduction properties of the axon are represented by quaternion sub structs, referred to as the axonal conduction sub structs

$$Ta_i = (r_{ij}, l_i, v_{ij}, t_i), \tag{3}$$

where r_{ij} is the axonal radius between nodes i and j. l_i is the path length from node i to the axon hillock node, which is the sum of the path length from node j to the axon hillock node and the path length between nodes i and j

$$l_i = l_j + l_{ij} = l_j + \sqrt{(x_i - x_j)^2 + (y_i - y_j)^2 + (z_i - z_j)^2}. \tag{4}$$

v_{ij} is the action potential conduction velocity between nodes i and j, which is proportional to $r_{ij}^{0.5}$. The conduction velocity of action potentials along the axon can be roughly estimated by $v_{ij} = \alpha r_{ij}^{0.5}$. The velocity coefficient α varies depending on the morphology and function of the neuron and can be determined through simple electrophysiological experiments. t_i represents the time it takes for the action potential to propagate from the axon hillock to node i. The calculation formula is given by

$$t_i = t_j + \frac{l_{ij}}{v_{ij}} = t_j + \frac{\sqrt{(x_i - x_j)^2 + (y_i - y_j)^2 + (z_i - z_j)^2}}{v_{ij}}. \tag{5}$$

Therefore, the axon node is a nine-dimensional set composed of two sub structs

$$A_i = \{S_i, Ta_i\} = (i, j, x_i, y_i, z_i; r_{ij}, l_i, v_{ij}, t_i),\ i = 1, 2, \cdots, n_{\mathrm{a}}, j = 0, 1, \cdots, n_{\mathrm{a}} - 1. \tag{6}$$

The function of dendrite node is to receive stimuli and aggregate them into the soma (Fig. S2d). The majority of neuronal dendrites are passively conducted. The intensity of the electrical signal on the dendrites gradually decays as the conduction distance increases. After the input of a transient pulse current I_{e} into the dendritic terminal, the increment of the membrane potential (IMP) [2] is

$$V_{\mathrm{m}}(I_{\mathrm{e}},l,t)=\frac{I_{\mathrm{e}}R_{\lambda}}{\sqrt{4\pi\lambda^2 t/\tau_{\mathrm{m}}}}\exp(-\frac{\tau_{\mathrm{m}}l^2}{4\lambda^2 t})\exp(-\frac{t}{\tau_{\mathrm{m}}}), \tag{7}$$

where t is time, l is the distance from the synaptic terminal and $R_\lambda = R_{\mathrm{L}}\lambda / \pi r^2$. The time constant $\tau_{\mathrm{m}} = R_{\mathrm{m}}C_{\mathrm{m}}$ and the electrotonic length $\lambda = (rR_{\mathrm{m}} / 2R_{\mathrm{L}})^{1/2}$ set the scale of the temporal and spatial variation of the membrane potential, where r is radius, R_{m} is resistance across a unit area of membrane, R_{L} is specific resistance of the internal medium and C_{m} is capacitance of membrane.

The conduction properties of dendrites are represented by a ternary sub structs, referred to as the dendritic conduction sub structs

$$Td_i=(r_{ij},l_i,V_{\mathrm{m}i}), \tag{8}$$

where r_{ij} and l_i are defined the same as in Equation (3). $V_{\mathrm{m}i}$ is the IMP at the node i

$$V_{\mathrm{m}i}=\sum_{ik}V_{\mathrm{m}ik}(I_{\mathrm{e}ik}(t),l_{ik}-l_i,t), \tag{9}$$

where the subscript ik denotes the k-th dendrite ending connected to node i, $I_{eik}(t)$ represents the current spectrum equivalent to the external stimulus. The IMP at the dendritic root node is represented as

$$V_{\mathrm{m}1}(t)=\sum_{1k}V_{\mathrm{m}1k}(I_{\mathrm{e}1k}(t),l_{1k},t). \tag{10}$$

Therefore, the dendrite node is an eight-dimensional set composed of two sub structs

$$D_i=\{S_i,Td_i\}=(i,j,x_i,y_i,z_i;r_{ij},l_i,V_{\mathrm{m}i}),\ i=-1,-2,\ \ldots,-n_{\mathrm{d}}, j=0,-1,\ \ldots,-(n_{\mathrm{d}}-1). \tag{11}$$

The soma node contains the global eigen parameters of the current neuron, including the coordinates of the soma, threshold potential, the time constant, the electrotonic length, the waveform of the action potential and so on (Fig. S2e).

The synapse node includes the coordinates of the synapse, the axonal terminal node of the presynaptic neuron, the dendritic terminal node of the postsynaptic neuron, the stimulus intensity coefficient, the time of neurotransmitter transmission and so on (Fig. S2f).

Through the cooperation among the above nodes, the neuron also needs to realize the function of receiving and integrating information by synaptic integration. Neurons have

numerous connections through synapses to receive input information from other neurons. A chemical synapse transmission requires approximately 0.3 to 0.5 ms. The amplitude of a single postsynaptic potential (PSP) is typically insufficient to trigger an action potential. Multiple synapses must undergo spatial or temporal integration to reach the threshold potential of the neuron (Fig. S2g).

The unit of neurotransmitter release from a synapse is the total amount of neurotransmitter contained in a single vesicle. The PSP is an integer multiple of the unit PSP generated by a single vesicle, meaning that the PSP is quantized. For the sake of analytical simplicity, the peak value of the unit IMP can be set to 0.1 mV. The unit IMP can be represented as

$$V_{\mathrm{m0}}(l,t) = V_{\mathrm{m}}(l,t) = 0.1\exp(-\frac{\tau_{\mathrm{m}} l^2}{4\lambda^2 t})\exp(-\frac{t}{\tau_{\mathrm{m}}})\ \mathrm{mV}. \tag{12}$$

When receiving a single stimulus, the IMP can be represented as

$$V_{\mathrm{m}}(t) = k_{\mathrm{V}} V_{\mathrm{m0}}(l,t),\ k_{\mathrm{V}} \in Z, \tag{13}$$

where k_{V} is stimulus intensity coefficient (an integer), representing that the IMP is k_{V} times the unit IMP. When k_{V} is positive, it represents an excitatory postsynaptic potential (EPSP), and when k_{V} is negative, it represents an inhibitory postsynaptic potential (IPSP).

The IMP of a neuron can be considered as the spatiotemporal sum of EPSP and IPSP. Assume that neuron i is connected to j axons and continuously receives input from these axons. At time t_{n}, the spatial sum of the external inputs received by the dendritic root node can be represented as

$$V_{\mathrm{m1}}(t) = \sum_{1k} k_{\mathrm{V}1k} V_{\mathrm{m0}}(l_{1k}, t - t_{\mathrm{n}}),\ 1k = 1, 2,\ \ldots, j, \tag{14}$$

where $k_{\mathrm{V}1k}$ is the stimulus intensity coefficient of synapse $1k$. When the dendritic root node receives input at time t_{n}, the IMP will be updated as follows to describe the temporal sum of the external inputs

$$V_{\mathrm{m1\_new}}(t)=\begin{cases} V_{\mathrm{m1}}(t), & t<t_{\mathrm{n}}, \\ V_{\mathrm{m1}}(t_{\mathrm{n}})+\sum_{1k}k_{\mathrm{V1}k}V_{\mathrm{m0}}(l_{1k},t-t_{\mathrm{n}}), & t_{\mathrm{n}}\le t\le t_{\mathrm{n}}+\dfrac{\tau_{\mathrm{m}}l_0}{2\lambda}, \\ (V_{\mathrm{m1}}(t_{\mathrm{n}})+\sum_{1k}k_{\mathrm{V1}k}V_{\mathrm{m0}}(l_{1k},\dfrac{\tau_{\mathrm{m}}l_0}{2\lambda}))\dfrac{V_{\mathrm{m0}}(l_0,t-t_{\mathrm{n}})}{V_{\mathrm{m0}}(l_0,\dfrac{\tau_{\mathrm{m}}l_0}{2\lambda})}, & t>t_{\mathrm{n}}+\dfrac{\tau_{\mathrm{m}}l_0}{2\lambda}, \end{cases} \tag{15}$$

$$V_{\mathrm{m1\_new}}(t)=\begin{cases} V_{\mathrm{m1}}(t), & t<t_{\mathrm{n}}, \\ V_{\mathrm{m1}}(t)+\sum_{1k}k_{\mathrm{V1}k}V_{\mathrm{m0}}(l_{1k},t-t_{\mathrm{n}}), & t_{\mathrm{n}}\le t<t_{\mathrm{n0}} \\ V_{\mathrm{m1}}(t_{n0})+\sum_{1k}k_{\mathrm{V1}k}V_{\mathrm{m0}}(l_{1k},t-t_{\mathrm{n}}), & t_{\mathrm{n0}}\le t\le t_{\mathrm{n}}+\dfrac{\tau_{\mathrm{m}}l_0}{2\lambda}, \\ (V_{\mathrm{m1}}(t_{\mathrm{n}})+\sum_{1k}k_{\mathrm{V1}k}V_{\mathrm{m0}}(l_{1k},\dfrac{\tau_{\mathrm{m}}l_0}{2\lambda}))\dfrac{V_{\mathrm{m0}}(l_0,t-t_{\mathrm{n}})}{V_{\mathrm{m0}}(l_0,\dfrac{\tau_{\mathrm{m}}l_0}{2\lambda})}, & t>t_{\mathrm{n}}+\dfrac{\tau_{\mathrm{m}}l_0}{2\lambda}, \end{cases} \tag{16}$$

or

$$V_{\mathrm{m1\_new}}(t)=\begin{cases} V_{\mathrm{m1}}(t), & t<t_{\mathrm{n}}, \\ V_{\mathrm{m1}}(t)+\sum_{1k}k_{\mathrm{V1}k}V_{\mathrm{m0}}(l_{1k},t-t_{\mathrm{n}}), & t_{\mathrm{n}}\le t<t_{\mathrm{n}}+\dfrac{\tau_{\mathrm{m}}l_0}{2\lambda} \\ V_{\mathrm{m1}}(t)+\sum_{1k}k_{\mathrm{V1}k}V_{\mathrm{m0}}(l_{1k},\dfrac{\tau_{\mathrm{m}}l_0}{2\lambda}), & t_{\mathrm{n}}+\dfrac{\tau_{\mathrm{m}}l_0}{2\lambda}\le t\le t_{\mathrm{n0}}, \\ (V_{\mathrm{m1}}(t_{\mathrm{n}})+\sum_{1k}k_{\mathrm{V1}k}V_{\mathrm{m0}}(l_{1k},\dfrac{\tau_{\mathrm{m}}l_0}{2\lambda}))\dfrac{V_{\mathrm{m0}}(l_0,t-t_{\mathrm{n}})}{V_{\mathrm{m0}}(l_0,\dfrac{\tau_{\mathrm{m}}l_0}{2\lambda})}, & t>t_{\mathrm{n}}+\dfrac{\tau_{\mathrm{m}}l_0}{2\lambda}, \end{cases} \tag{17}$$

where t_{n0} is the time at which the neuron's membrane potential reached its peak prior to the update. When the neuron's membrane potential has already reached its peak before the update, the IMP should be updated using Equation (15) to reflect the cumulative effect of incoming inputs. If the neuron's membrane potential has not reached its peak before the update, the IMP should be updated using Equation (16) or (17) according to the magnitude of t_{n0} and $t_{\mathrm{n}}+\tau_{\mathrm{m}}l_0 / 2\lambda$.

During continuous synaptic integration, the conditions that trigger the action potential also need to be considered. In the digital neuron, the excitability of the neuron should be considered during three periods (quiescence, refractory and subnormal period) with relatively long

durations. During the quiescence period, the action potential will be triggered when the IMP satisfies the following condition

$$\max(V_{\mathrm{m1}}(t)) \geq V_{\mathrm{th}} - V_{\mathrm{rest}}, \tag{18}$$

where V_{th} is the threshold of membrane potential and V_{rest} is resting membrane potential. V_{th} is typically 15 to 30 mV higher than V_{rest}. During the refractory period, the neuron will not trigger a new action potential regardless of the stimulus intensity. This period typically lasts for 0.5 to 1 ms. During the subnormal period, the neuron requires a stronger stimulus to reach the threshold potential since the membrane potential is below the resting potential. This period typically lasts for about 10 ms depending on the different neurons.

On the basis of synaptic integration, synaptic plasticity can be considered on a longer spatiotemporal scale. For each synapse, the action potential frequency f from the axon can be linked to stimulus intensity coefficient k_{V} to simulate synaptic plasticity

$$\begin{aligned} k_{\mathrm{V}}(f(t)) &= k_{\mathrm{V0}} + k_{\mathrm{VSTP}}(t) + k_{\mathrm{VLTP}}(t) - k_{\mathrm{VLTD}}(t) \\ &= k_{\mathrm{V0}} + A_{\mathrm{s}}\int_0^t e^{-\frac{t-\tau}{\tau_{\mathrm{s}}}} f(\tau)\mathrm{d}\tau + A_{\mathrm{p}}\int_0^t e^{-\frac{t-\tau}{\tau_{\mathrm{p}}}} \sigma\left(\frac{\bar{f}(\tau) - f_{\mathrm{LTP}}}{\Delta f}\right)\mathrm{d}\tau - A_{\mathrm{d}}\int_0^t e^{-\frac{t-\tau}{\tau_{\mathrm{d}}}} \sigma\left(\frac{f_{\mathrm{LTD}} - \bar{f}(\tau)}{\Delta f}\right)\mathrm{d}\tau \end{aligned} \tag{19}$$

where the average stimulation frequency over a certain period of time τ_f is

$$\bar{f}(t) = \frac{1}{\tau_f}\int_0^t e^{-\frac{t-\tau}{\tau_f}} f(\tau)\mathrm{d}\tau \tag{20}$$

σ is sigmoid function. k_{V0} is the basic stimulus intensity. k_{VSTP} represents short-term synaptic plasticity, indicating short-term enhancement or short-term inhibition. It rises rapidly with f, but will quickly decay due to the small value of τ_{s}. k_{VLTP} represents long-term potentiation. When the average stimulation frequency exceeds the threshold f_{LTP}, this item gradually increases. k_{VLTD} represents long-term depression. When the average stimulation frequency is lower than the threshold f_{LTD}, this item gradually increases. Short-term synaptic plasticity occurs on the millisecond to second scale, while long-term potentiation and long-term depression occur on the minute to hour scale, i.e., $\tau_{\mathrm{s}} \ll \tau_{\mathrm{s}}, \tau_{\mathrm{s}}$.

1.2 Fitting of the action potential

In later simulations, only a few parameters of the action potentials triggered by the digital neuron would affect the simulation of electrophysiological activities, including threshold potential, resting potential, absolutely refractory period and subnormal period. The above parameters can be obtained from the experimental data of neuron electrophysiology. The details of the action potential waveform will not affect the simulation results. However, to visualize the final electrophysiological activity, the general membrane voltage dynamics can be adopted to fit the action potential.

At present, the idea of Hodgkin-Huxley model [3-7] is mostly used for the fitting of the action potential. For neurons with different properties in different species, it is necessary to change the description of ion channels in the model and fit their parameters. The neuronal membrane can be equivalent to a circuit

$$I_{\mathrm{m}} = C_{\mathrm{m}} \frac{\mathrm{d}V}{\mathrm{d}t} + \sum_{k} I_k \, , \tag{21}$$

Where I_{m} is the membrane current, C_{m} is the membrane capacitance, V is the membrane voltage, and I_k is the k-channel current. Common ion currents in mammalian hippocampal pyramidal cells include dast transient sodium current (I_{Na}), delayed rectifier potassium current (I_{KDR}), A-type potassium current (I_{A}), voltage-gated calcium current (I_{Ca}), leak current (I_{L}) and so on. The ion current is expressed as

$$I_k = \bar{g}_k m^p h^q (V - E_k) \, , \tag{22}$$

where $\bar{g}_k$ is maximum conductance, m or h is the open probabilities of ion channel subunits, p or q is the number of individual subunits, E_k is the equilibrium voltage of the k-ion channel. According to the gated variable dynamics, there is

$$\frac{\mathrm{d}x}{\mathrm{d}t} = \alpha_x(V)(1-x) - \beta_x(V)x, \; x \in \{m, h\} \, , \tag{23}$$

where α_x is open rate constant, β_x is inactivation rate constant. This equation is equivalent to

$$\frac{\mathrm{d}x}{\mathrm{d}t} = \frac{x_\infty(V) - x}{\tau_x(V)}, \; x \in \{m, h\} \, , \tag{24}$$

where $\alpha_x = x_\infty/\tau_x$, $\beta_x = (1-x_\infty)/\tau_x$. Different neurons have different electrophysiological characteristics. The membrane dynamics model described above can be used to fit the waveform

of a single action potential. The fitting for different action potentials can be directly implemented by python or other neuron software.

1.3 Optimization of sphere distribution for NE

Determining the spatial distribution of neurons in the NE is crucial to achieve energy efficient communication. The three-dimensional distribution of densely connected small-scale NEs will be analyzed by mathematical optimization. Based on basic physical principles and biological phenomena, the arrangement of neurons can be parameterized into the following four criteria.

Firstly, neurons must not overlap with one another and require sufficient space to fulfill their normal metabolic requirements. Therefore, there is a minimum distance $l_{\min}$ between somas

$$(x_i - x_j)^2 + (y_i - y_j)^2 + (z_i - z_j)^2 \geq l_{\min}{}^2,\ 1 \leq i, j \leq n, i \neq j\ , \tag{25}$$

where x_i, y_i, z_i are the coordinates of the soma of neuron i ($1 \leq i \leq n$). The axons (or dendrites) are disjoint and at least $d_{\min}$ away from each other

$$(x'_{ip} - x'_{jq})^2 + (y'_{ip} - y'_{jq})^2 + (z'_{ip} - z'_{jq})^2 \geq d_{\min}{}^2,\ 1 \leq i, j \leq n, i \neq j\ , \tag{26}$$

where x_{ip}, y_{ip}, z_{ip} are the coordinates of the point p on neuron i.

Secondly, due to the limited space of the brain, the NE cannot be spread out indefinitely. Therefore, the NE is confined to a limited space

$$x_i^2 + y_i^2 + z_i^2 \leq R^2, \tag{27}$$

where R is the radius of the restricted space. To make the optimization case unique, the centroid of the NE is translated to the origin

$$\sum x_i = 0, \sum y_i = 0, \sum z_i = 0\,. \tag{28}$$

Thirdly, the topological connection of NE should remain unchanged during optimization. The probability of connections between neurons should follow the observed data. The probability of connecting adjacent neurons in the hippocampus and other brain regions used for memory analysis is about 30% to 50% [8]. Depending on the local small-scale NE, the

probability of connection is about 5% to 80%, which is higher than the total probability of connection for the whole cortex.

Fourthly, to ensure lower energy consumption, the sum of the length of axons (or dendrites) should be as short as possible. To ensure faster communication, the total path between neurons (where the common axons (or dendrites) of different connections are counted repeatedly) should be as short as possible. However, in the brain, energy consumption and communication are considered simultaneously. The connections from a single neuron to multiple sites generally share a path first and bifurcate at the tail to form a tree of axon and ensure low metabolic energy consumption and fast communication. Here, the connection that guarantees energy efficiency is considered first. Suppose that the axon of neuron i is linked to the dendrites of x neurons. It will form a set of endpoints

$$T_i = \{N_i, N_{j1}, N_{j2}, \dots, N_{jx}\}, \tag{29}$$

where N_i represents neuron i as the root node of the tree of axon, N_j ($j = j_1, j_2, \dots, j_x$) represents the leaf nodes of the tree of axon. When the coordinates (x_i, y_i, z_i) of the root and leaf nodes are known, the coordinates of the intermediate nodes of the binary tree of axon should be optimized to find the shortest path. Suppose that the set of intermediate nodes is

$$S_i = \{S_{i1}, S_{i2}, \dots, S_{ik}\}. \tag{30}$$

Then the set of nodes of the tree of axon is

$$V = T \cup S, \tag{31}$$

The set of edges of the tree is

$$E = \{(p,q) \mid p,q \in V\}. \tag{32}$$

The length of each edge is

$$L_{pq} = \sqrt{(x_p - x_q)^2 + (y_p - y_q)^2 + (z_p - z_q)^2}. \tag{33}$$

The objective function is to minimize the sum of the length of the edge

$$\min_{S(k)} L_{\text{total}} = \min_{S(k)} \sum_{(p,q)\in E} \sqrt{(x_p - x_q)^2 + (y_p - y_q)^2 + (z_p - z_q)^2}. \tag{34}$$

An important property of the optimal path is that the sum of the three edge vectors at each intermediate node is zero. This constraint can be written as

$$\sum_{S_h \in S, X_{hl} \in V} \frac{S_h - X_{hl}}{\| S_h - X_{hl} \|} = 0, \tag{35}$$

where X_{hl} is the node directly connected to S_h. Then the optimization equation can be expressed as

$$\begin{aligned} &\min_{S(k)} L_{\text{total}} = \min_{S(k)} \sum_{(p,q) \in E} \sqrt{(x_p - x_q)^2 + (y_p - y_q)^2 + (z_p - z_q)^2}, \\ &\text{subject to } \sum_{S_h \in S, X_l \in V} \frac{S_h - X_{hl}}{\| S_h - X_{hl} \|} = 0, \end{aligned} \tag{36}$$

According to the solution of the optimization equation (36), the shortest path for the axon of neuron i to connect to the dendrites of x neurons is defined as $L_{\min}(i, T_i)$.

Based on the above criterion, Spatial distribution of neurons in the NE of n neurons with minimum energy consumption can be given by optimization. The objective function is to minimize the sum of the connection paths of all neurons. This optimization process is an NP-hard problem with high computational complexity. Using fast or conventional algorithms generally only converges to suboptimal solutions. When the topological connection is determined, the optimization equation is

$$\begin{aligned} &\min \sum_i L_{\min}(i, T_i) \\ &\text{subject to } (x_i - x_j)^2 + (y_i - y_j)^2 + (z_i - z_j)^2 \ge l_{\min}{}^2,\ 1 \le i, j \le n, i \ne j, \\ &\qquad (x'_{ip} - x'_{jq})^2 + (y'_{ip} - y'_{jq})^2 + (z'_{ip} - z'_{jq})^2 \ge d_{\min}{}^2,\ 1 \le i, j \le n, i \ne j, \\ &\qquad \sum x_i = 0, \sum y_i = 0, \sum z_i = 0, \\ &\qquad x_i^2 + y_i^2 + z_i^2 \le R^2. \end{aligned} \tag{37}$$

In the case of full connection (p = 100%), optimization is performed by Equation (37). The optimal spatial distribution of neurons will constitute a sphere structure as shown in Fig. S2i-j. The soma nodes are evenly distributed on the surface of the sphere following the Fibonacci grid [9]. It is not only more consistent with the actual situation, but also beneficial to numerical calculation. In other topologically connected NEs, sphere structures are at least a suboptimal

case. Therefore, when NS is applied to small-scale NEs with high connection probability, the single-layer sphere structure can ensure high communication efficiency while taking into account the structural order.

1.4 Connection structure for NS

Based on the digital neurons and optimization conclusions, connecting multiple digital neurons into a structured spherical network can maximally simulate the energy efficient electrophysiological activity of NE. This model can be named figuratively as the neural sphere (NS). The primary structure of the NS is a unit sphere. The soma nodes of each digital neuron are distributed on the surface of the sphere, while the connections between neurons (axons → synapses → dendrites) are distributed within the sphere as shown in Fig. S2h.

The NS can be used to simulate real neural networks, such as a small ensemble of neurons in the cerebral cortex. In the NS, the sphere surface is equivalent to an unfolded cerebral cortex. Using topologically equivalent rules, the positions and connections of neurons are mapped onto the surface and inside of the sphere.

In addition, NS can also be used to design specialized ideal networks to study the principles of bio-intelligence. In a uniform ideal NS, the soma nodes are evenly distributed on the surface of the sphere following the Fibonacci grid [9]. Then the coordinates of n neurons on the sphere can be expressed as

$$\begin{cases} z_i = \dfrac{2i-1}{n} - 1, \\ x_i = \sqrt{1-z_i^2}\cos(2\pi i\phi), \\ y_i = \sqrt{1-z_i^2}\sin(2\pi i\phi), \end{cases} \tag{38}$$

where $i = 1, 2, \ldots, n$ and constant $\phi = (5^{1/2}-1)/2$. By setting the parameters of the digital neurons and the neuron connection matrix, NSs with different functions and the characteristics of electrical activity can be obtained.

1.5 Application range and scalability of NS

This NS model focuses on the structural and electrophysiological basis of neuronal information processing, including dendritic signal reception, synaptic integration, membrane-potential evolution, and axonal firing. At the mesoscopic spatiotemporal scale, it has restored the core electrical signal processing process of the neural ensemble as accurately as possible. Furthermore, several important mechanisms can be naturally incorporated as extended modules. For example, neural noise can be achieved through synaptic input or random terms in the dynamic process of membrane potential. The regulation of glial cells can be achieved through additional variables such as extracellular ion homeostasis, neurotransmitter clearance, and metabolic regulation.

In this work, the NS model focuses on considering the electrophysiological characteristics of NE on a short-timescale. The waveforms of the electrophysiological activities of specific neural systems at a specific moment under different initial conditions can be determined by controlling the variables. Therefore, it is necessary to fix the connection matrix of NE, as well as a series of characteristic parameters such as the action potential of each neuron, the stimulation intensity coefficient of each synapse, etc. Besides, the current framework should have clear scalability to enable further development towards more detailed multi-scale neural models in the future.

2. Implementation of numerical experiments

Based on NS, the numerical simulation of neural network action potential activity can be implemented by C++. This section will briefly describe the implementation process of the simulation program.

The program inputs include a basic parameter table, initial conditions, digital neuron files, and a neuron synaptic connection file. The basic parameter table is used to set global parameters such as the number of neurons, the maximum simulation time, and the time step. The initial conditions specify the excitation state of each neuron at the beginning, including states such as resting, pre-action potential, during action potential, post-action potential and so on. Each neuron in the network has an independent digital neuron csv file.

After receiving input, the state of each node in every neuron will be simulated at each time step. The simulation process proceeds as follows: update the dendritic input (update dendrite nodes), update the membrane potential (synaptic integration), and update the axonal output (output action potentials). After reaching the maximum simulation time, the program will output the membrane potential of each neuron over time.

In all numerical experiments, the following morphological parameters of all neurons are assigned the same values, including soma radius, dendrite radius, axon radius, average dendrite length, and average axon length. These parameters are obtained through statistical analysis of the morphological data of pyramidal neurons in the monkey cerebral cortex from the digitally reconstructed neurons and glia database, neuromorpho.org [1] and pyramidal neurons in the human hippocampus [10]. The fit of action potentials is used for visual analysis of electrophysiological activities. In some of the numerical experiments, randomly connected neural networks are employed. The random parameters in the randomly connected network include synaptic connection strength, neuron connection probability p, etc. Unless otherwise specified, the initial condition is that the first neuron triggers an action potential at 0.00 ms, the maximum simulation time is 1000 ms, and the time step is 0.01 ms.

In order to determine the correlation between the membrane potential time series of two neurons, it is necessary to design a reasonable correlation analysis method. Suppose that the membrane potential time series of two neurons i and j with the same time length are $V_i\ (t)$ and $V_j\ (t)$, respectively. Due to the different initial conditions, even the same two membrane potential time series will have a time difference. Therefore, it is necessary to translate one of the time series by time t_0, and then calculate the Pearson correlation coefficient ρ of the overlap part of the two time series. The correlation coefficient ρ_{m} between $V_i\ (t)$ and $V_j\ (t)$ can be expressed as

$$\rho_{\mathrm{m}}(V_i(t),V_j(t)) = \max_{t_0}\{\rho(V_i(t-t_0),V_j(t))\},\ 0 \le t_0 \le T\ , \tag{39}$$

where T is the period of the membrane potential time series.

3. Hysteresis complex iteration theory of simplified neural sphere (NS) dynamical system

It is necessary to characterize the dynamical principles of NS to drive different potential activities. Depending on the initial conditions, NS can generate different types of potential

activity to simulate the electrophysiological activity of the NE. Since the full initial condition simulation through the original NS requires unacceptable computing power and time, a simplified NS dynamical system can be constructed to restore the main biological links of electrophysiological activities in NE. The simplified NS dynamical system consists of the simplified action potential, the simplified synaptic connection, and the hysteretic complex iterative process.

The simplified action potentials can be reduced to the basic feature of all-or-nothing by a step function ε and a Dirac delta function δ, as shown in Fig. S2k. The simplified action potential function of a neuron can be expressed as

$$U(\tau) = \varepsilon(\tau_{\mathrm{r}} + t - \tau)\delta(\tau - t), \tag{40}$$

where U is the equivalent state of the membrane potential of the neuron, τ_{r} is absolute refractory period and t is the moment when the action potential is triggered.

The simplified synaptic connections can be represented by the connection matrix $\boldsymbol{K}_{\mathrm{V}}$

$$\boldsymbol{K}_{\mathrm{V}} = \begin{bmatrix} \boldsymbol{K}_{\mathrm{V},1} \\ \boldsymbol{K}_{\mathrm{V},2} \\ \cdots \\ \boldsymbol{K}_{\mathrm{V},n} \end{bmatrix} = \begin{bmatrix} K_{11} & K_{12} & \cdots & K_{1n} \\ K_{21} & K_{22} & \cdots & K_{2n} \\ \cdots & \cdots & \cdots & \cdots \\ K_{n1} & K_{n2} & \cdots & K_{nn} \end{bmatrix}, \tag{41}$$

where the real part of K_{ij} represents the sum of connection strengths of all synapses from the axon of neuron i to the dendrite of neuron j, and the imaginary part of K_{ij} represents the hysteretic time of signal transmission from the axon to the dendrite. In particular, K_{ij} is 0 when there is no connection.

According to the above simplification, the simplified NS dynamical equation can be constructed to essentially describe the dynamic process of continuous hysteretic iterative interaction among neurons. The state variable of each neuron can be described by a complex number

$$z = V(\cos\tau + i\sin\tau), \tag{42}$$

where V is the equivalent potential state of the neuron and τ is time. Since τ is a small quantity compared to V, the following approximation can be made when plotting the phase portrait

$$z \approx V + i\tau \,. \tag{43}$$

Therefore, the simplified NS dynamical equation can be expressed as

$$z_{k+1,i}(\tau) = (a_i - z_{k,i}(\tau))g(K_{ij} z_{k,j}(\tau) - \Phi_i)\varepsilon(\tau_{\mathrm{r}} + kt_{\mathrm{min}} - \tau)\delta(\tau - kt_{\mathrm{min}}) \,, \tag{44}$$

where i is the index of the neuron and g is the sigmoid function. This tensor expression consists of a control term $(a_i - z_{k,i})\ g(K_{ij}z_{k,j} - \Phi_i)$ and an action potential term $\varepsilon(\tau_{\mathrm{r}} + kt_{\mathrm{min}} - \tau)\ \delta(\tau - kt_{\mathrm{min}})$, where k is the iteration step and t_{min} is the time resolution. The real part of a_i, Re (a_i), is used to regulate whether the neuron triggers an action potential. Depending on the change of Re(z), Re (a_i) made the control term is close to 1 or 0. The imaginary part of a_i, Im (a_i), is used to set the hysteretic time of synaptic integration for neuron i. The real number Φ_i is the threshold of neuron i. Each complex iteration of the dynamical system is equivalent to the synaptic integration of each neuron for several hysteresis inputs from the remaining connected neurons. The dynamics of the simplified NS dynamical system is regulated by three groups of control parameters, including a_i, K_{ij} and Φ_i. If only the simplest interaction of neurons in NS is considered (that is, each neuron is only subjected to the hysteresis effect of the connected neurons), the hysteresis dynamical equation of NS can be further simplified as

$$z_{k+1,i}(\tau) = (a_i' - z_{k,i}(\tau))(K_{ij}' z_{k,j}(\tau) - \Phi_i') \,, \tag{45}$$

where a_i', K_{ij}' and Φ_i' are the equivalent control parameters of a_i, K_{ij} and Φ_i after affine transformation, respectively.

4. High-performance neuromorphic computing architecture of brain

According to the information features of the NS, a high-performance neuromorphic computing architecture of brain is constructed to describe a possible working mode of the brain (Fig. S3). The architecture is mainly composed of two kinds of NS, including processing NS of memory NS. The processing NS exhibits the characteristic of random potential activity (box II in Fig. S3). It can directly receive a string of multisensory stimulus or processed external information and generate self-organized information through aperiodic action potential activity. The memory NS exhibit the characteristic of long period potential activity (box III in Fig. S3). Through the regulation of short-term synaptic plasticity from a group of neurons, the input to the

memory NS is a group of action potentials that trigger sequentially at specific moments (box I in Fig. S3). It is equivalent to the group of membrane potential states of all neurons at a particular instant in the periodic potential waveform (the red dashed line of Fig. S4). Through the set of input information from outside the NS forms a one-to-one mapping with the set of initial conditions, the input information can be stored in the NS.

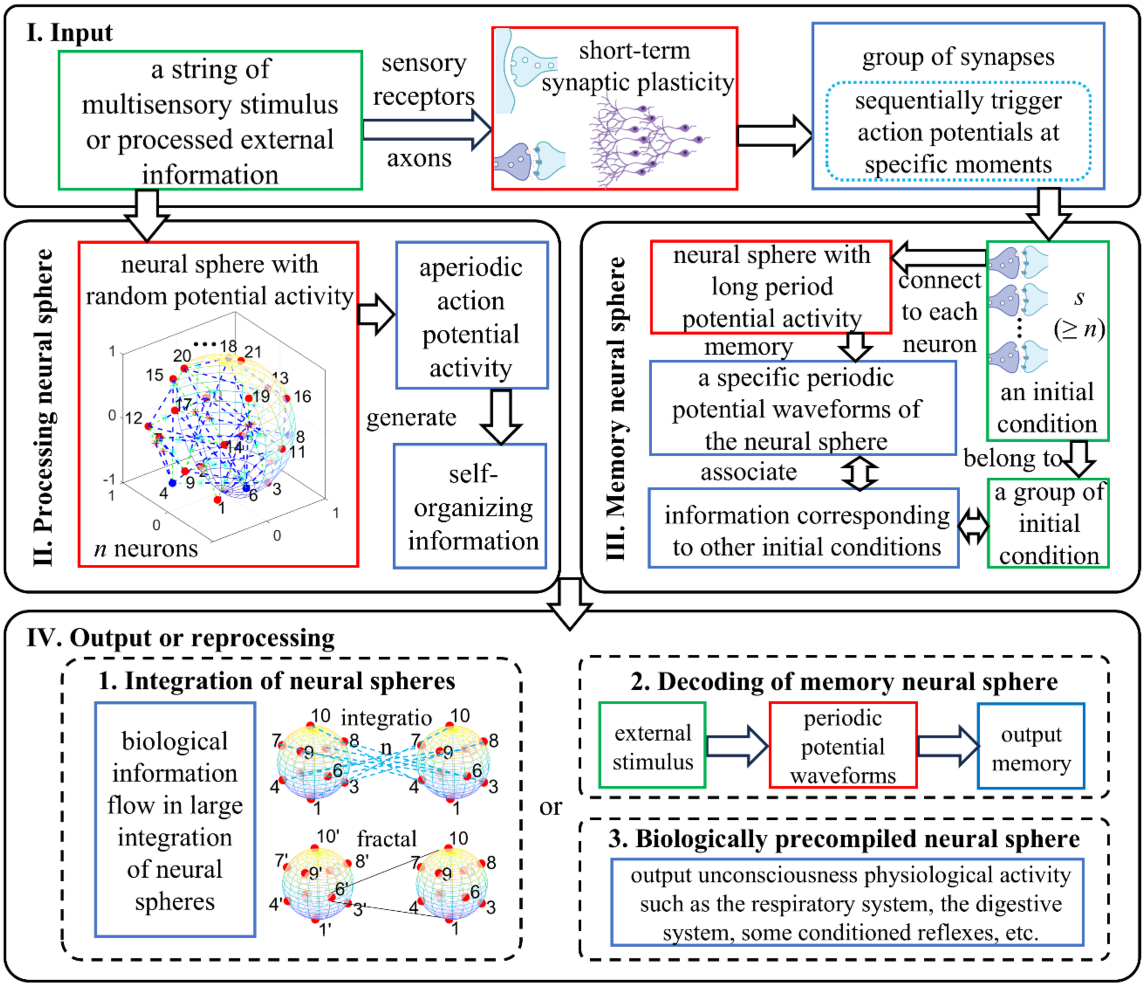

Fig. S3. The operation mechanism of high-performance neuromorphic computing architecture of brain. The black rounded boxes show the four modules. Each module can be independent or integrated with each other. The green, red and blue boxes represent the input driver, processing logic and output driver, respectively. In box III, *s* is the number of synapses. In box IV.1, the cyan dashed lines represent left-to-right or right-to-left synaptic connections between neurons.

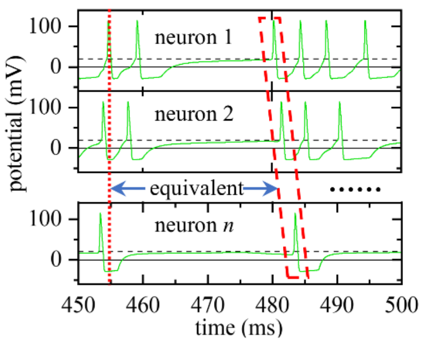

Fig. S4. Schematic diagram of two equivalent initial conditions.

The information processed or stored by NS can be directly output or reprocessed (Box IV in Fig. S3). (1) Through the integration of small-scale NS with different structures and functions, the biological information flow will be iteratively processed and memorized across different NS. In particular, the integration of NSs can be designed as a multi-layer fractal structure. The integration comprising one layer of fractal structures, which includes *m*' sub NSs each containing *m* neurons, is defined as fractal of $m' \times m$. And so on, the integration comprising *n* layer of fractal structures can be represented as fractal of $m^{(n)} \times m^{(n\text{-}1)} \times \ldots \times m'' \times m' \times m$. The box IV.1 in Fig. S3 shows that 10 sub NSs of 10 neurons form a NS of 100 neurons through one-layer of fractals, i.e., fractal of 10'×10. (2) By re-inputting the external stimulus, the memory NS will produce a periodic potential waveform to output the memory. (3) The NS can be precompiled to have specific physiological functions through long-term evolution. A group of effectors connected to it can output unconsciousness physiological activity such as the respiratory system, the digestive system, some conditioned reflexes, etc.

5. Quantification of information in the neural sphere (NS) and the artificial systems

Using the principles of information theory, the data storage capacity of the NS and the artificial system are quantified. Detailed estimations are presented in Supplementary information.

In 1948, Shannon used the concept of entropy to quantify information and named it self-information [11]. The self-information *I* is represented as

$$I = -\sum_i p_i \log_2 p_i,\ i = 1, 2,\ \ldots\ , q\ , \tag{46}$$

where p_i is the probability that event *i* occurs and *q* is the total number of events.

In current computing architectures, manual computing systems for large-scale applications are all binary encoded. Each device has only two states. In this case, the self-information of these artificial systems can be represented as

$$I = -\sum_{i} (p_i \log_2 p_i + (1-p_i)\log_2(1-p_i)),\ i = 1, 2,\ \ldots\ , n, \tag{47}$$

where p_i is the probability that the device i is in one of the binary states. The value of I is from 0 to n bit.

In the NS, the number of events in the self-information corresponds to the number of periodic potential waveforms. And each periodic potential waveform corresponds to an initial condition group. If the state of a neuron at a certain time is intercepted as an initial condition, the number of initial states of a neuron consists of four terms

$$\begin{aligned} &S_{\text{neuron}} = S_{\text{Q}} + S_{\text{I}} + S_{\text{R}} + S_{\text{S}}, \\ &S_{\text{Q}} = 1,\ S_{\text{I}} = \frac{V_{\text{th}} - V_{\text{rest}}}{\max(V_{\text{m0}})} \frac{\tau}{t_{\min}},\ S_{\text{R}} = \frac{t_{\text{R}}}{t_{\min}},\ S_{\text{S}} = \frac{t_{\text{S}}}{t_{\min}}, \end{aligned} \tag{48}$$

where S_{Q}, S_{I}, S_{R} and S_{S} are the number of states of the neuron during quiescence, incubation, refractory, and subnormal periods, respectively. V_{th} is the threshold of membrane potential and V_{rest} is resting membrane potential. $\max(V_{\text{m0}})$ is the peak of the membrane potential generated by one vesicle. τ is the time when the membrane potential reaches its peak from 0. t_{R} and t_{S} are the durations of the refractory and subnormal periods, respectively. $t_{\min}$ is the minimum temporal resolution of the neuron. If the minimum temporal resolution of the neurons can reach the physical limit, $t_{\min}$ will take Planck time, $5.39116(13)\times10^{-44}$ s. Then the total number of initial conditions of the NS consisting of n neurons is

$$S_{\text{ensemble}} = (S_{\text{neuron}})^{s}, \tag{49}$$

where s ($\geq$ n) represents the number of external synapses that trigger the action potential activity of the ensemble. The number of initial conditions corresponding to different periodic potential waveforms is also different. The average number of initial conditions of the periodic potential waveform can be expressed as

$$S_{\mathrm{T}} = \frac{\sum_{j} T_j / t_{\min}}{S_{\mathrm{ensemble}}}, \ j = 1, 2, \ \dots, S_{\mathrm{ensemble}}, \tag{50}$$

where T_j is the period of action potential activity at initial condition j. Besides, the synaptic plasticity in the NS will multiply the number of initial conditions. The total number of states of external synapses can be defined as

$$S_{\mathrm{synapse}} = (n_{\mathrm{v}} + 1)^s, \tag{51}$$

where n_{v} represents the total number of states for synaptic release vesicles. "+1" represents a state in which no vesicles are released. Then the number of periodic potential waveforms contained by the NS can be estimated as

$$S_{\mathrm{total}} = \frac{S_{\mathrm{ensemble}} S_{\mathrm{synapse}}}{S_{\mathrm{T}}}, \tag{52}$$

In a set of NS, the number of states of the NS can be estimated as

$$S_{\mathrm{total}} = \prod_{j=1}^{m} \frac{S_{\mathrm{ensemble}}(j) S_{\mathrm{synapse}}(j)}{S_{\mathrm{T}}(j)}, \tag{53}$$

where m is the number of sub NS. The $S(j)$ with different subscripts represent the corresponding number of states of the sub NS j.

As for the NS of the fractal of $m' \times m$ ((Box IV.1 in Fig. S3), the number of states of the NS can be estimated as the product of three terms

$$S_{\mathrm{total}} = \left(\prod_{j=1}^{m'} S_{\mathrm{total}}(j)\right)\left(S_{\mathrm{synapse}} - \prod_{j=1}^{m'} S_{\mathrm{synapse}}(j)\right)\left(\prod_{j=1}^{m'} \frac{S_{\mathrm{T}}(j)}{t_{\min}}\right), \tag{54}$$

where the first term represents the sum of the number of sub NS states, the second term represents the synaptic plasticity of the connections among sub NSs, and the third term represents the number of states of the periodic potential waveform of the sub NS. Therefore, the self-information of the NS of n neurons is

$$I' = -\sum_{i} (p_i' \log_2 p_i'), \ i = 1, 2, \ \dots, S_{\mathrm{total}}, \tag{55}$$

where p_i' is the probability of generating waveform i.

For fractal structures with more layers (>2), Equation (49) can be rewritten as

$$S_{\text{ensemble}}{}^{(p+1)} = (S_{\text{total}}{}^{(p)})^{s^{(p+2)}}, \tag{56}$$

where $S_{\text{ensemble}}{}^{(p+1)}$ is the number of states of the ensemble of fractal at level p+1. $S_{\text{total}}{}^{(p)}$ is the total number of states of fractal at level p. $s^{(p+2)}$ is the number of synaptic connections between sub ensembles in the fractal at level p+2.

Besides, according to the analysis of the NS with periodic potential activity, the number (or self-information) of initial conditions will be greater than the number (or self-information) of periodic potential waveforms in NS. The number of distinct initial condition matrices as shown in Fig. 2b is equivalent to the number of states of the initial condition. Its limitations include the following four aspects. Firstly, the time at which the next action potential triggers will be after the last one in the same neuron, i.e., $t_{ji} > t_{ki}, j > k$. Secondly, the time difference between two adjacent triggered action potentials is greater than the refractory period in the same neuron, i.e., $t_{ji} > t_{\text{R}}$. Thirdly, the final t_{ji} is supplemented with "/" when the action potential is fired less than q times. Fourthly, the final non-"/" t_{ji} should be less than the period of the periodic potential waveform of NS, i.e., $t_{ji} < T$. Based on the above limitations, the number of the initial condition matrices is

$$S_{\text{matrix}} = \left(\sum_{j=1}^{q} \frac{([\frac{T}{t_{\min}}] - (j-1)[\frac{t_{\text{R}}}{t_{\min}}])!}{j!([\frac{T}{t_{\min}}] - (j-1)[\frac{t_{\text{R}}}{t_{\min}}] - j)!}\right)^{n}, \tag{57}$$

where

$$q = [[\frac{T}{t_{\min}}] \Big/ [\frac{t_{\text{R}}}{t_{\min}}] + 1], \tag{58}$$

and “[ ]” represents the rounding operation. Then the self-information of the initial condition is

$$I'' = -\sum_{i} (p_i'' \log_2 p_i''),\ i = 1, 2,\ \ldots, S_{\text{matrix}}. \tag{59}$$

Then the ratio of I'' and I' can measure the information compression rate or feature extraction amount of NS.

6. Evaluation of the computational power for neural sphere (NS)

The computational power for floating-point operation of NS can be estimated by two equivalent methods. Detailed estimations are presented in Supplementary information.

In NSs with random action potential activity, the action potential output of one neuron can be equivalent to several floating-point operations. The computational power of NS can be expressed as

$$\mathrm{FLOPS}_{\mathrm{HNCA}} = n \times \mathrm{FLOP}_{\mathrm{HNCA}} \times f\,, \tag{60}$$

where $\mathrm{FLOP}_{\mathrm{NS}}$ is the number of floating point operations equivalent to a single action potential. n is the number of the neuron. f is the frequency of the action potential. An action potential spike is the result of dendritic synaptic integration of that neuron. Suppose that the input to each synapse is equivalent to a floating point number. Then an action potential is equivalent to (the number of synapses -1) floating-point operations. in this equivalent procedure, the precision of one floating-point operation is $\log_2(\tau_m / t_{min})$.

In NSs with periodic action potential activity, the total amount of memory operated per unit time can be equivalent to several floating-point operations. The computational power of NS can be expressed as

$$\mathrm{FLOPS}_{\mathrm{HNCA}} = \frac{I}{3\mu T}\,, \tag{61}$$

where I is the self-information or storage capacity of the NS. "3" represents the occupancy that requires two inputs and one output for one floating-point operation. μ is the precision of a floating-point number. A double precision floating-point number has 64 bit of precision. T is the period of action potential activity of the NS.

7. Physical limit of the power for human brain by neural sphere (NS)

Based on Landauer's principle [12, 13], the power physical limit of human brain simulated by NS can be analyzed. In Landauer's principle, the energy required to erase a bit has a minimum value, which is called Landauer limit

$$E_{\min} = k_B T \ln 2\,, \tag{62}$$

where k_B is the Boltzmann constant and T is the temperature of system in Kelvin. Based on the estimation of the maximum computational power of the human brain P_{max} by NS, the theoretical minimum power of the human brain can be obtained by the Landauer limit

$$\begin{aligned} E_{\text{human brain}} &= k_B T \ln 2 \times P_{\max} \times 2\varepsilon_P \\ &= 1.38\times10^{-23} \times (273.15+37) \times \ln 2 \times 6.24\times10^{18} \times 2\times 64 = 2.37 \text{ W}, \end{aligned} \tag{63}$$

where T takes the body temperature 37 °C, $2\varepsilon_P$ is the number of bits occupied by a double-precision floating-point operation.

Based on the existing experimental data, the energy composition of the human brain (15~20 W) can be divided into three parts [14], including computing and communication (≈ 3 W), maintaining body temperature (≈ 9 W), and energy consumption for other biological processes such as metabolism (≈ 3~8 W). Among them, the computing and communication power of the human brain is only 1.26 times that of the Landauer limit.

The energy-efficiency estimation in this work is based on a coarse-grained mapping from neural electrophysiological activity to equivalent information-processing events. Specifically, the number of periodic electrophysiological activities is used to estimate the effective information capacity of the brain, while the characteristic electrophysiological period is used to estimate the corresponding computational rate. It provides an order-of-magnitude equivalent computational measure under explicitly defined assumptions. The real neural computation is continuous, nonlinear, and multiscale. Therefore, the calculated Landauer-limit power represents a theoretical lower-bound reference for the equivalent amount of irreversible information processing, rather than an exact physical limit of brain energy dissipation. The comparison between this theoretical lower-bound power and the actual metabolic power of the brain should therefore be interpreted as a coarse-grained energy-efficiency indicator, not as a precise thermodynamic efficiency of the human brain.

Notes

Note 1 The parameter selection of digital neurons

The parameter settings for digital neurons are presented in Table S1. All values are estimated from statistical analyses of experimental observations [1] [10] of real neurons. Morphological data of some neurons are presented in Table S2. According to Method 1.2, the parameters of the action potentials were derived from human hippocampal neurons [10] as shown in Fig. S5. Different neurons have different fitting parameters.

Type	Parameter	Description	Value
Neural ensemble parameters (the monkey cerebral cortex [1] and the human hippocampus [10] digital neuron database)	n	The number of neurons	/
	s	The number of synapses	/
	p	The probability of connection	0-100%
	η_i	The proportion of inhibitory neurons	5%-15%
Parameters of neurons (the monkey cerebral cortex [1] and the human hippocampus [10] digital neuron database)	V_{th}	The threshold potential that triggers an action potential	-50 mV
	V_{rest}	Resting potential	-70 mV
	τ_m	Membrane time constant	20 ms
	λ	Membrane length constant	1000 μm
	t_s	Synaptic response time	0.5 ms
	l_a	Average axon length based on database statistics	700 μm
	l_d	Average dendrite length based on database statistics	300 μm
	r_a	Average axon radius based on database statistics	0.2 μm
	r_d	Average dendrite radius based on database statistics	0.55 μm

Action potential parameters [10]	t_r	Absolutely refractory period	0.61 ms
	t_s	Subnormal period	11.73 ms

Table S1. The parameter settings of digital neurons for NS numerical simulations.

NMO index for monkey	Radius of dendrite (μm)	Length of dendrite (μm)	Radius of axon (μm)	Length of axon (μm)
1 (01911)	0.527328	264.965	0.37918	621.087
2 (35129)	0.557319	301.176	0.254106	1256.24
3 (35130)	0.427115	330.985	0.219363	473.237
4 (149530)	0.528261	226.785	0.144715	491.611
5 (149531)	0.728944	318.901	0.211443	804.431
6 (149532)	0.700106	370.764	0.261524	1043.58
7 (149535)	0.506562	255.191	0.18006	972.19
8 (149537)	0.614571	310.844	0.256278	549.454
9 (149538)	0.522000	184.208	0.213849	414.821
average	0.568023	284.8688	0.235613	736.2946
median	0.528261	301.176	0.219363	621.087

NMO index for human	Radius of dendrite (μm)	Length of dendrite (μm)	Radius of axon (μm)	Length of axon (μm)
1 (276157)	0.4576	308.538	0.2542	632.151
2 (277047)	0.3813	386.528	0.2288	432.525
3 (276044)	0.6147	382.840	0.2431	364.121
4 (276020)	0.5147	389.200	0.2648	706.393
5 (276156)	0.7181	368.508	0.1941	744.817
average	0.5147	382.840	0.2542	632.151
median	0.5372	367.123	0.2370	576.001

Table S2. Morphological data of some of monkey cerebral cortex neurons [1] and the human hippocampal neurons [10].

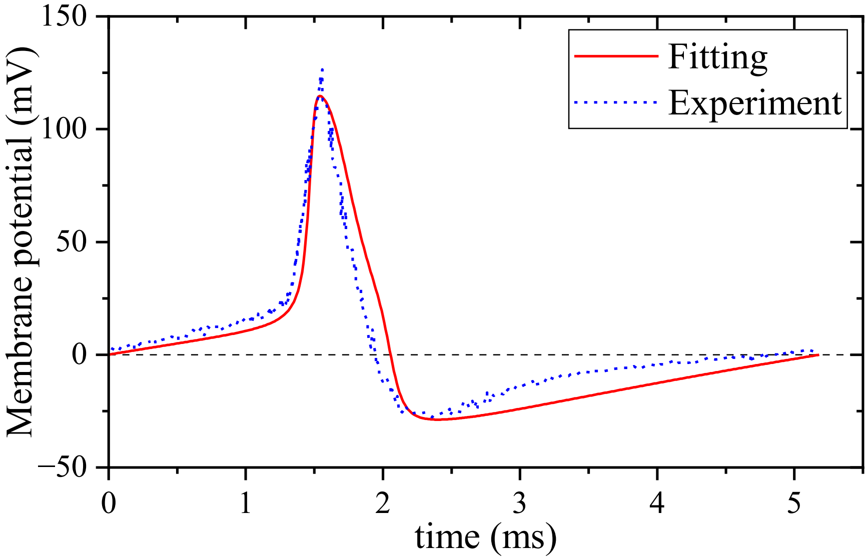

Fig. S5. An example of the fitting of action potential based on the human hippocampal neurons [10]. The resting potential (-70 mV) is parallel to 0 mV.

Note 2 Optimization results of sphere distribution for neural ensemble (NE)

According to the optimization equation (37), the optimization process is an NP-hard problem with high computational complexity. The exact optimal solution can be obtained by exhaustive search. In an NE with n neurons, m synapses, and k intermediate nodes of axons or dendrites, the time complexity is $O([2R/\Delta]^{3(n+m+k)})$. Δ is the minimum error of the coordinates. It is an unacceptable time complexity. Therefore, by combining dynamic programming and genetic algorithms, a suboptimal solution with a small error from the optimal solution can be obtained within a limited time. This optimization strategy is detailed in the following.

Firstly, the optimization problem (37) can be split into two steps: (1) Fix the coordinates of the soma of neurons (x, y, z), and optimize the coordinates of all nodes of axons and dendrites (x', y', z'); (2) Fix the coordinates of all nodes of axons and dendrites (x', y', z'), and optimize the coordinates of the soma of neurons (x, y, z). These two steps are alternated in turn until the difference between adjacent objective functions is less than 10^{-5} for 10 consecutive steps. Secondly, according to the principle of dynamic programming, each step can be further divided into optimization of several sub-problems. The optimization result of i-node problem is used as the initial condition to further optimize the i+1-node problem. The optimization results of all i-node sub problems being stored can greatly reduce the optimization time. The optimization will be from i = 2 to i = the number of nodes that need to be optimized. Thirdly, for the optimization of each i-node sub-problem, genetic algorithm or other intelligent algorithms can be used to quickly converge. The optimization of genetic algorithm can directly use toolbox “GADS” in MATLAB or library “DEAP” in Python. The maximum iteration step is set to be greater than 1000 and the cutoff error is set to be less than 1e-5. Besides, for the local optimization problem (36), since the number of nodes involved is not large, it can be obtained by exhaustive search. Due to the setting of the maximum iteration steps, the above optimizations can be done in polynomial time.

According to the above optimization method, it is found the sphere distribution of neuronal soma positions will make the NE of symmetric topology connection consumes the least energy. Take the topologically symmetrical fully connected NE as an example (Fig. S6a), the nodes of the neuron soma are symmetrically distributed on the sphere surface. The other nodes are

symmetrically distributed in the sphere. The optimization process is shown dynamically in Movie S1.

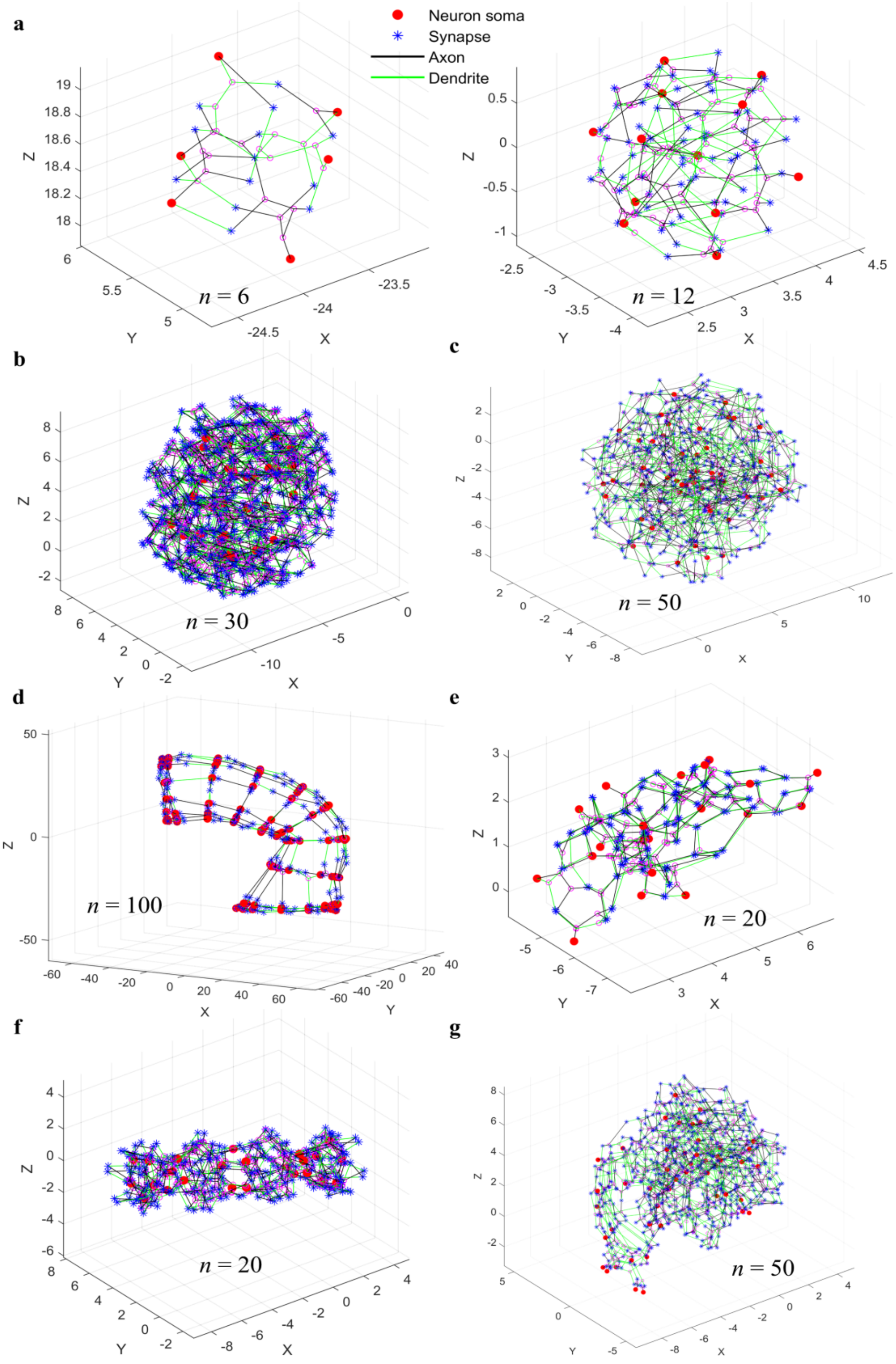

Fig. S6. Optimal structure of NE with different topological connections. (a) Fully connected structure. (b) “Small-world” network connected structure. (c) Random connected structure. (d) Single-layer connection structure. (e) Two-layer connection structure. (f) Multi-layer connection structure. (g) Fully bias connection structure.

Besides, some optimized structures of asymmetric topological connections will present other structures that retain partial spherical features. For example, the optimal distributions of the asymmetric NE of “small-world” network structure (Fig. S6b) and the random connection structure (Fig. S6c) all present a nearly sphere structure. The optimal distribution of the single-layer structure NE (Fig. S6d) can show the folds on the surface of the cerebral cortex. The optimal distribution of the two-layer structure NE (Fig. S6e) shows the three-dimensional spoon-shaped structure of the layered neural connections in the local cerebral cortex. The optimal distribution of multi-layer connected NE (Fig. S6f) shows the most common cortical column structures in the brain. The optimal distribution of the fully biased connection structure (Fig. S6g), which is completely opposite to the symmetrical structure, presents a cap-shaped structure. In summary, in the optimal neural distribution of NE, the weights of the local energy constraints in the three coordinate directions depend on the local topological connections. For instance, the optimal distribution of the column structure (Fig. S6f) indicates that the concentrated connections within the layers and the coefficient connections between layers result in the energy constraints within the layers dominating the optimal distribution. It causes the inter-layer connections to stretch the nearly spherical structures within the layers, thereby forming an overall column structure.

When the number of neurons is small, the optimal solution can be obtained through exhaustive search. According to the error analysis in Table S3, the error between the suboptimal solution and the optimal solution is all less than 0.5%. The average error is 0.1785%.

Connection type	The number of neurons	Optimal solution	Suboptimal solution	Relative error
Fully connected structure	5	9.1536	9.1656	0.1309%
	6	12.9551	12.9713	0.1249%
	7	19.7149	19.7554	0.2054%
	8	25.4622	25.4967	0.1353%
	9	32.1250	32.1895	0.2005%
	10	46.6047	46.7059	0.2170%
	5	10.6151	10.6330	0.1684%

Random connected structure	6	16.2090	16.2426	0.2067%
	7	24.8560	24.8992	0.1734%
	8	33.4519	33.5108	0.1759%
	9	45.0257	45.1132	0.1942%
	10	59.6897	59.8148	0.2094%

Table S3. Error analysis of suboptimal and optimal solutions in NE optimization. The solution is represented by the result of the sum of the shortest length of axons and dendrites. The value of length is relative length, which can be scaled up equally according to the real neuron morphology.

This study focuses on the waveform characteristics of electrophysiological activities. The optimized structures of NEs are designed to be as close as possible to the actual situation in terms of setting the length ratio of the nerve projections. Different neural distributions have only a limited effect on specific electrophysiological waveforms, and have almost no impact on the overall characteristics of the large collection of electrophysiological activities under various initial conditions. Therefore, in the subsequent numerical experiments, a symmetric structure NE has been used to construct NS model.

Note 3 Some fragmented near-neural-sphere (near-NS) structures in biological systems

In order to save energy and space as much as possible and adapt to complex functional requirements, some distributions of neurons are a large number of nesting and stacking of incomplete near-NS structures. Based on morphological data, certain near-NS structures can be initially decoupled. For example, the neural ring of Caenorhabditis elegans is composed of multiple layers of torus (Fig. S7a). Due to the limitations of the pharynx, the distribution of neurons needs to make compromises and cannot be a perfect sphere. Some neural distributions in the visual cortex of mouse are a nearly sphere surface without upper and lower spherical crowns (Fig. S7b). Some nearly spherical neuronal clusters can also be isolated from the hippocampus of mouse (Fig. S7c). Moreover, depending on the different topological connections, there are numerous column structures in the cerebral cortex [15], ranging from tens of micrometers to even larger, as well as nearly spherical structures that are a few micrometers or even smaller [16].

A meaningful NS structure requires that the corresponding neuronal cluster has relatively independent functions. However, the morphology data of neurons cannot be used to determine the functions of neuron clusters. Datasets that can integrate structure and function are extremely rare at present. In the above example, the neural ring, which is equivalent to the brain of the Caenorhabditis elegans, has clearly defined and independent functions. Therefore, in order to achieve the decoupling of neural structures, a large amount of experiments and analyses are still required.

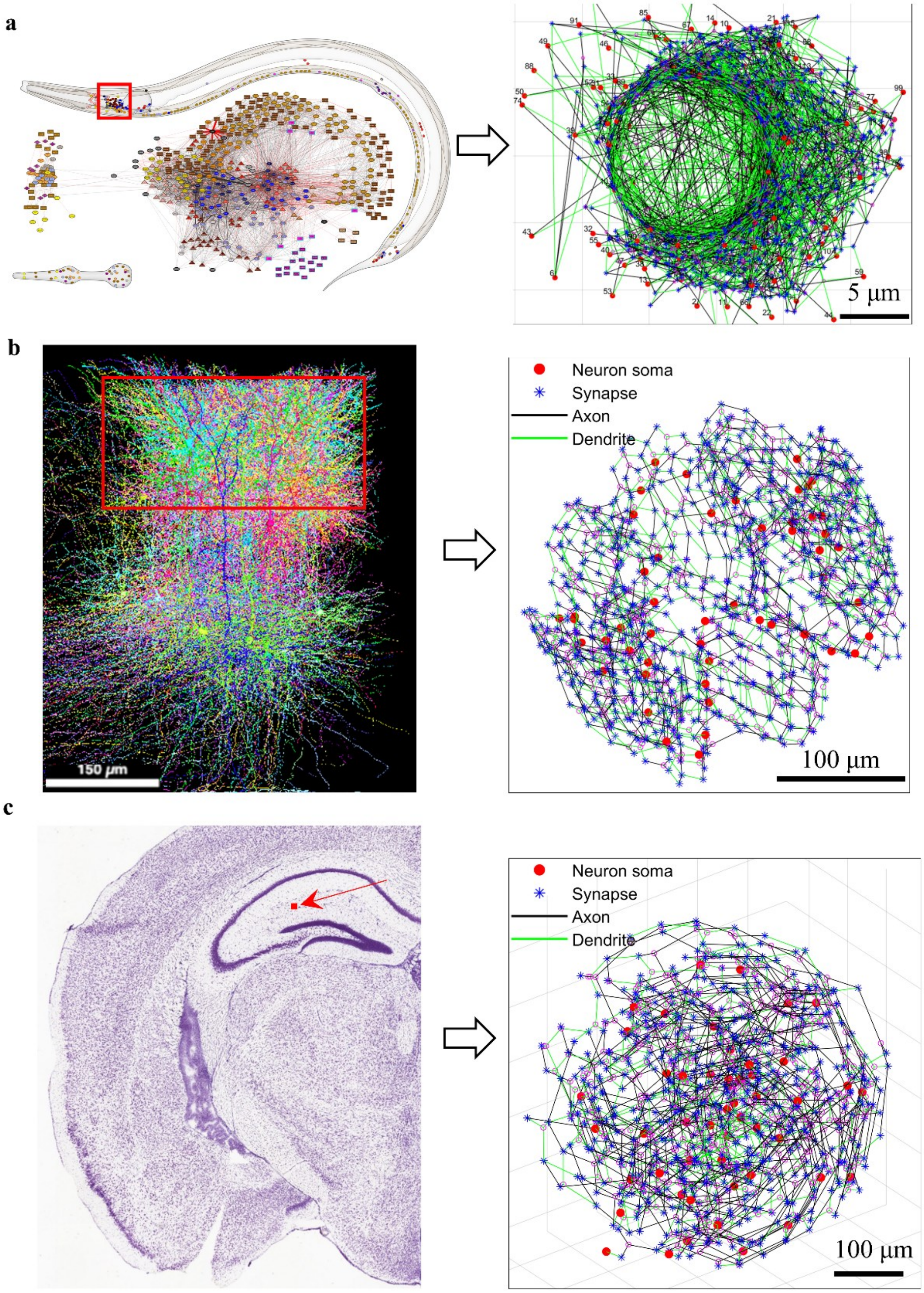

Fig. S7. Three examples of fragmented near-NS structures in biological systems. The left side of each subfigure shows a global view of the biological neural systems. The right side of each subfigure shows the distribution of local neurons (red box), represented by the digital neurons. (a) The neural ring of Caenorhabditis elegans [17]. (b) Local visual cortex of the mouse [18]. (c) Local hippocampus of the mouse [18].

Note 4 Connection & initial condition affect the electrophysiological activity of neural sphere (NS)

Through a series of systematic simulations of small-scale NSs, it has been found that the characteristics of electrophysiological activity are significantly affected by the connection probability p and initial condition. Taking the NS of 10 digital neurons as an example, Figure S8 shows the membrane potential activities of one of the neurons in the NS. The time step t_{min} is 0.01 ms. The digital neuron parameters adopt the data from Note 1. The radius of NS is adjusted so that the average lengths of axons and dendrites are l_{a} and l_{d}, respectively.

NS with different connection probabilities exhibit four typical membrane potential activities. In the bidirectional fully connected NS (p = 100%, Fig. S8a), digital neurons exhibit high-frequency action potential firing, approximately 340 Hz. According to the first peak of the autocorrelation coefficient function [19], the minimum period of action potential activity is 2.95 ms. This frequency is in the "ultra-fast" oscillatory band (200-600 Hz) [20] of the mammalian cerebral cortex. Local NEs with high probability of connection will be suitable for rapid responses with repeated high frequencies. In the unidirectional fully connected NS (p = 50%, Fig. S8b), the average and maximum action potential frequency are 76 Hz and 120 Hz, respectively. The action potential activity of this NS is in the oscillatory band of the gamma wave [20]. It does not have obvious periodicity or has an ultra-long period that exceeds the maximum simulation time. This random action potential activity implies that NEs will generate self-organizing information to support information processing and cognitive functions. In the sparsely connected NS (p = 20%, Fig. S8c), the digital neurons return to resting state after triggering a few action potentials. It is suitable for structurally simple local NE that provides transient reflections. In the full positive feedback connected NS (Fig. S8d), the action potential activity has an ultra-long period, reaching 288.11 ms, which is on the same level as the average human reaction time [21]. The average and maximum action potential frequency are 167 Hz and 209 Hz, respectively, belonging to high-frequency gamma waves [20]. It means that a large amount of ordered information is able to be stored in the NE through periodic changes in action potential activity to realize the learning and memory functions of the brain.

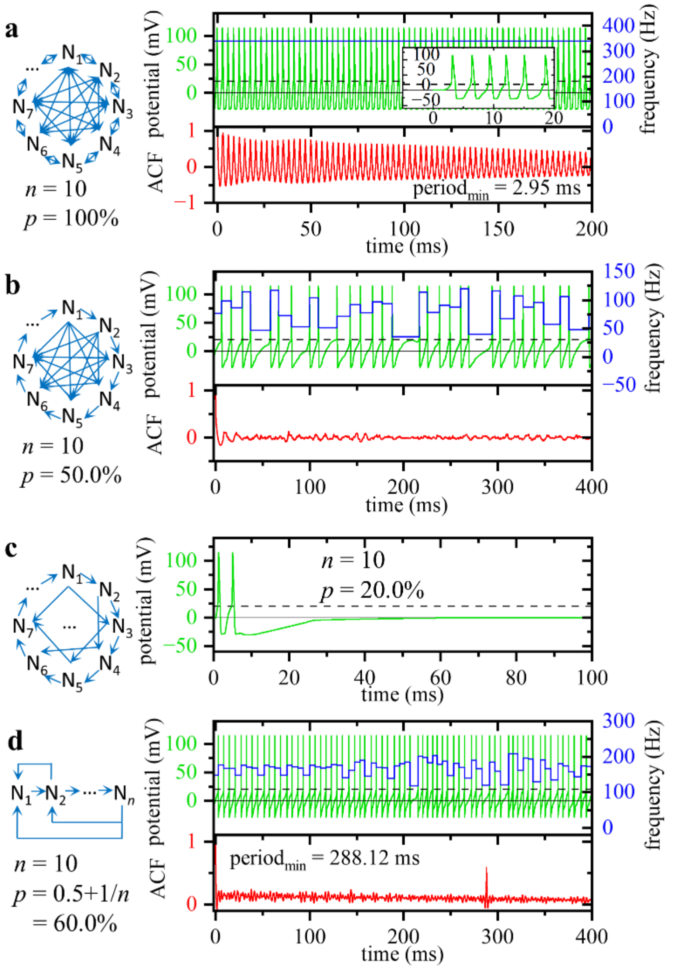

Fig. S8. The membrane potential activity of NS. (**a**) Bidirectional fully connected NS. (**b**) Unidirectional fully connected NS. (**c**) Sparsely connected NS. (**d**) Full positive feedback connected NS. (Upper left) Planar schematic of the connection types. Arrows are synaptic connections from axons to dendrites. N_i is the digital neuron with index i. (Lower left) The number of digital neurons n, and the probability of the connection p. (Right) The solid green line is the membrane potential time series of one of the digital neurons. The black dashed line is the threshold potential, 20 mV. The blue solid line is the real-time frequency spectra of action potential as a function of time. The red solid line is the curve of the autocorrelation coefficient function, ACF.

Besides, comprehensive membrane potential activity data are presented in this section. Figures S9-S12 correspond to the four subfigures of Fig. S8, respectively. The minimum period of the potential activity is at the first non-noise significant peak of the autocorrelation coefficient.

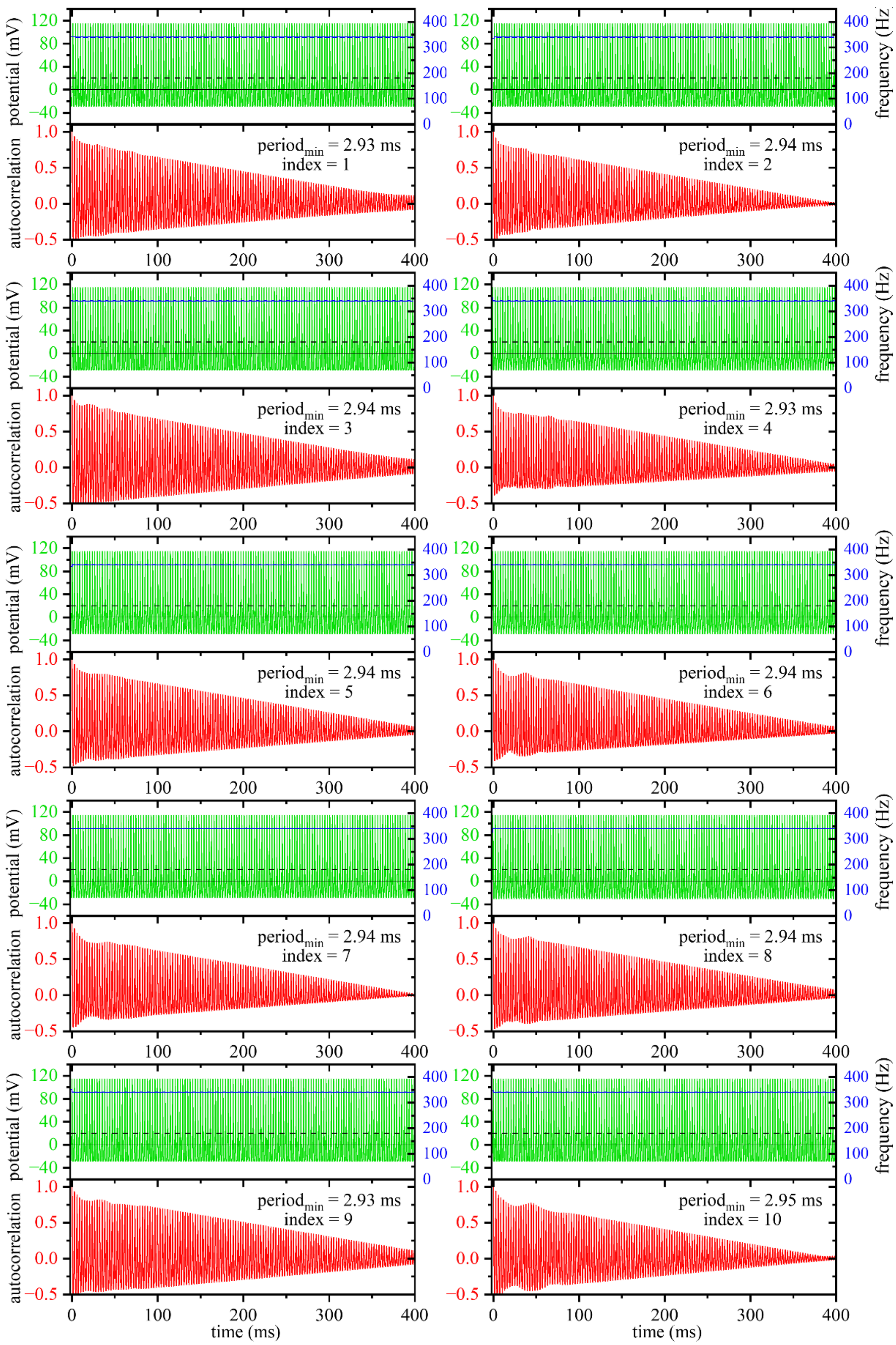

Fig. S9. Data for all neurons of the bidirectional fully connected NS.

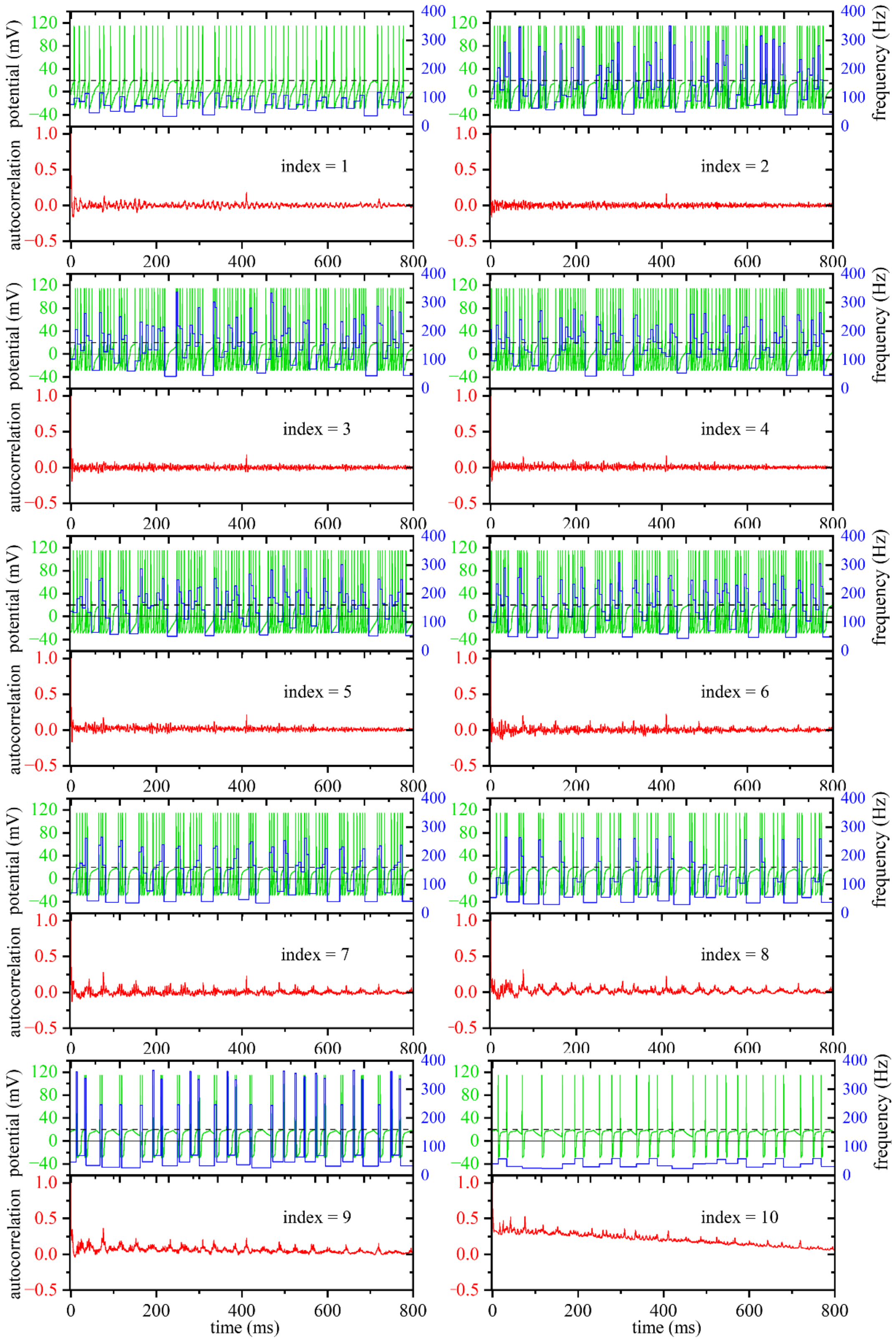

Fig. S10. Data for all neurons of the unidirectional fully connected NS.

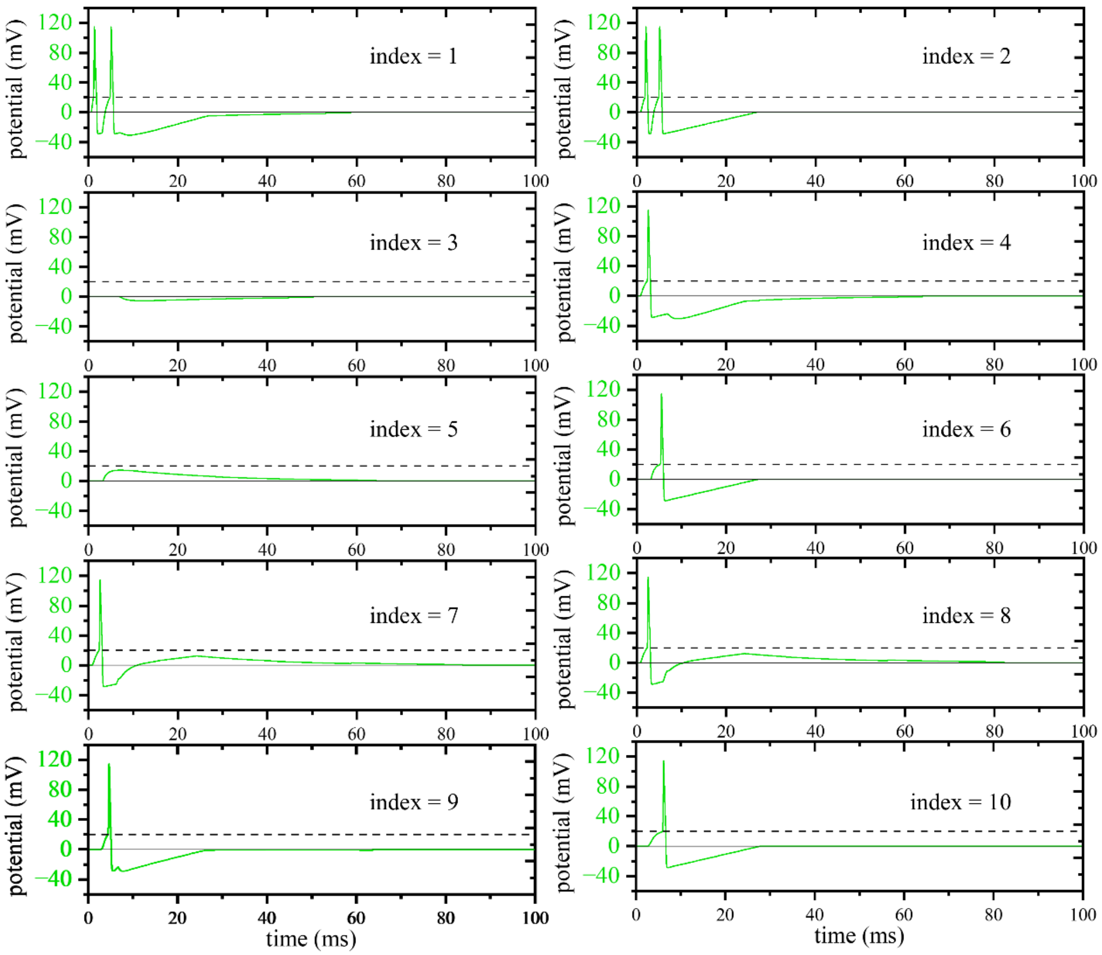

Fig. S11. Data for all neurons of the sparsely connected NS.

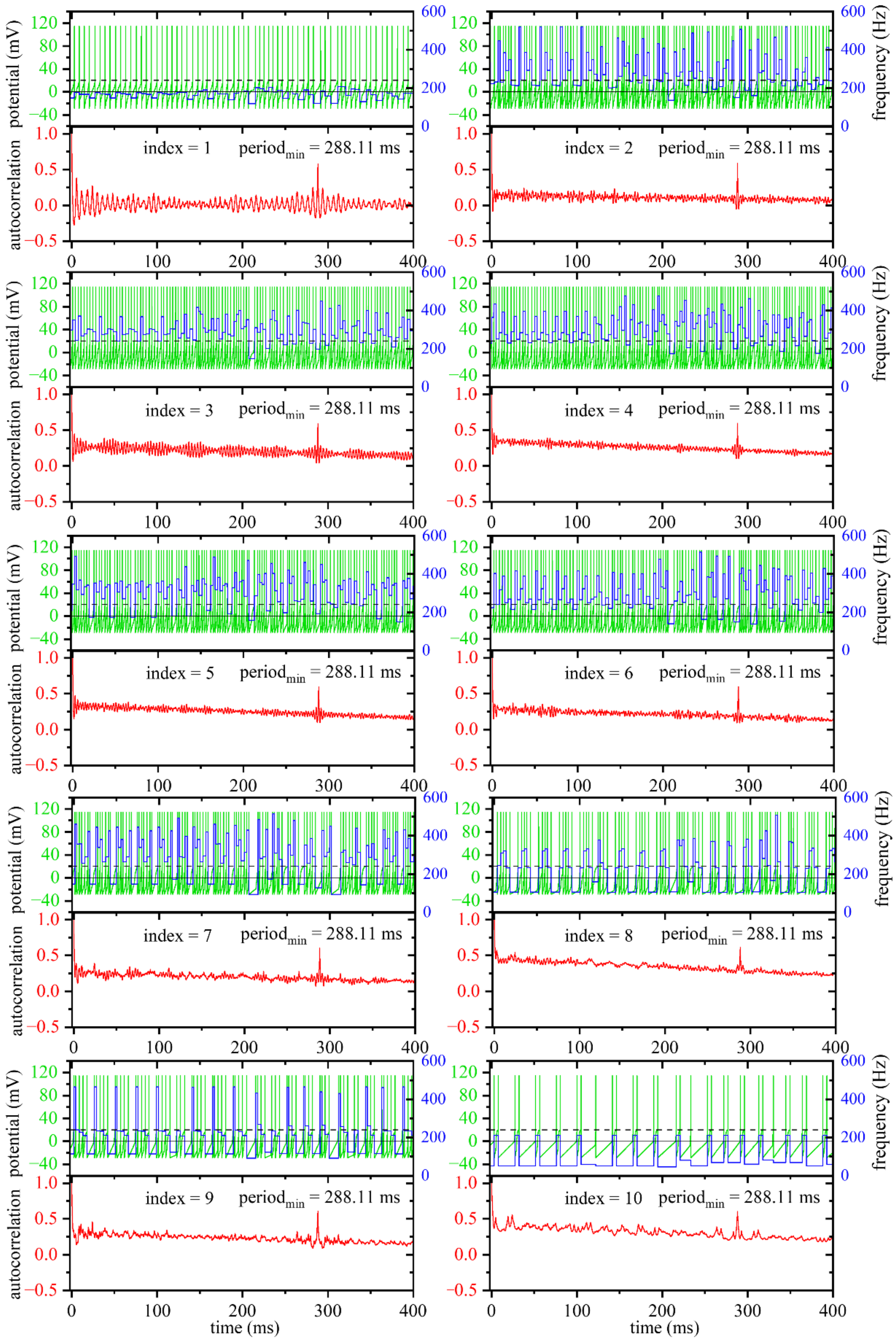

Fig. S12. Data for all neurons of the full positive feedback connected NS.

Furthermore, figures S13-S21 are the membrane potential activity data for the full positive feedback connected NS of nine different initial conditions to supplement Fig. 2c. When the initial condition is that the 10th neuron triggers an action potential at 0.00 ms, NS cannot generate continuous electrophysiological activities. Therefore, this situation is not recorded.

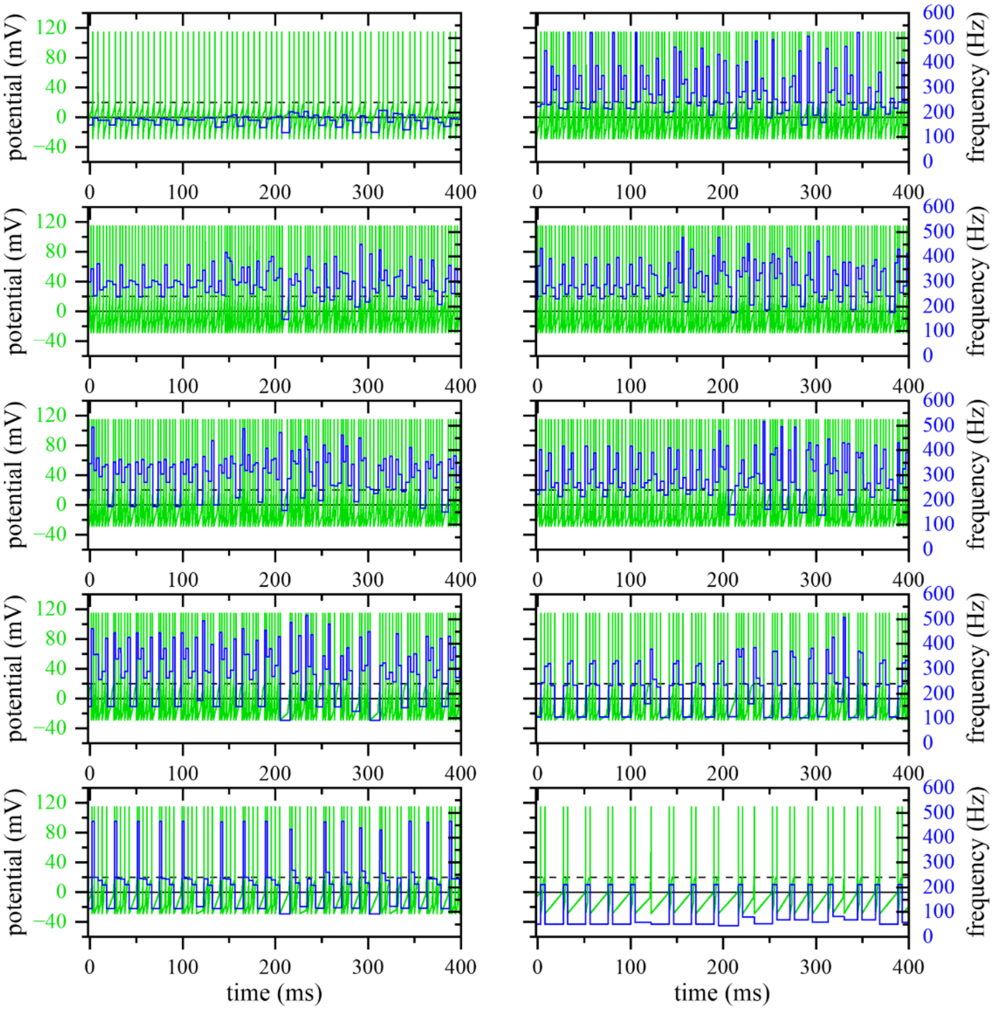

Fig. S13. Potential activity of the full positive feedback connected NS of 10 neurons. The initial condition is that the 1st neuron triggers an action potential at 0.00 ms.

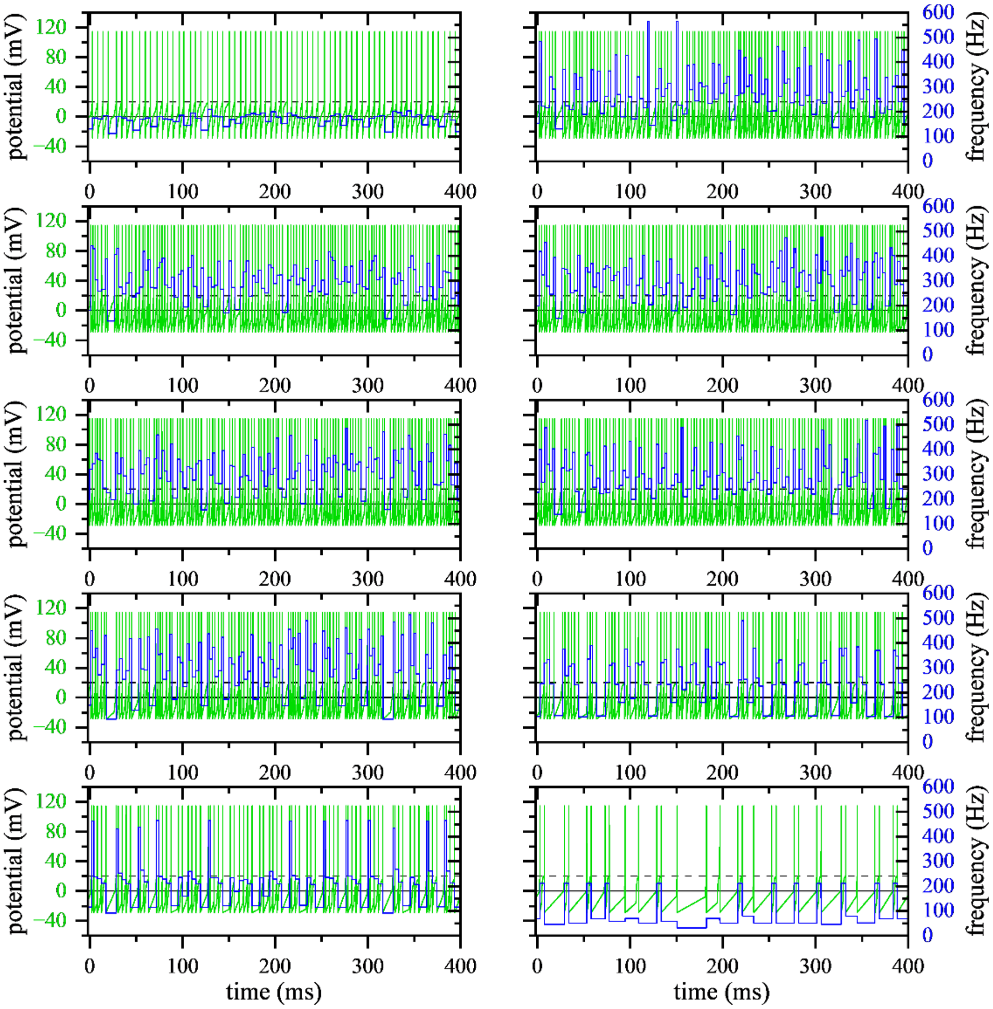

Fig. S14. Potential activity of the full positive feedback connected NS of 10 neurons. The initial condition is that the 2nd neuron triggers an action potential at 0.00 ms.

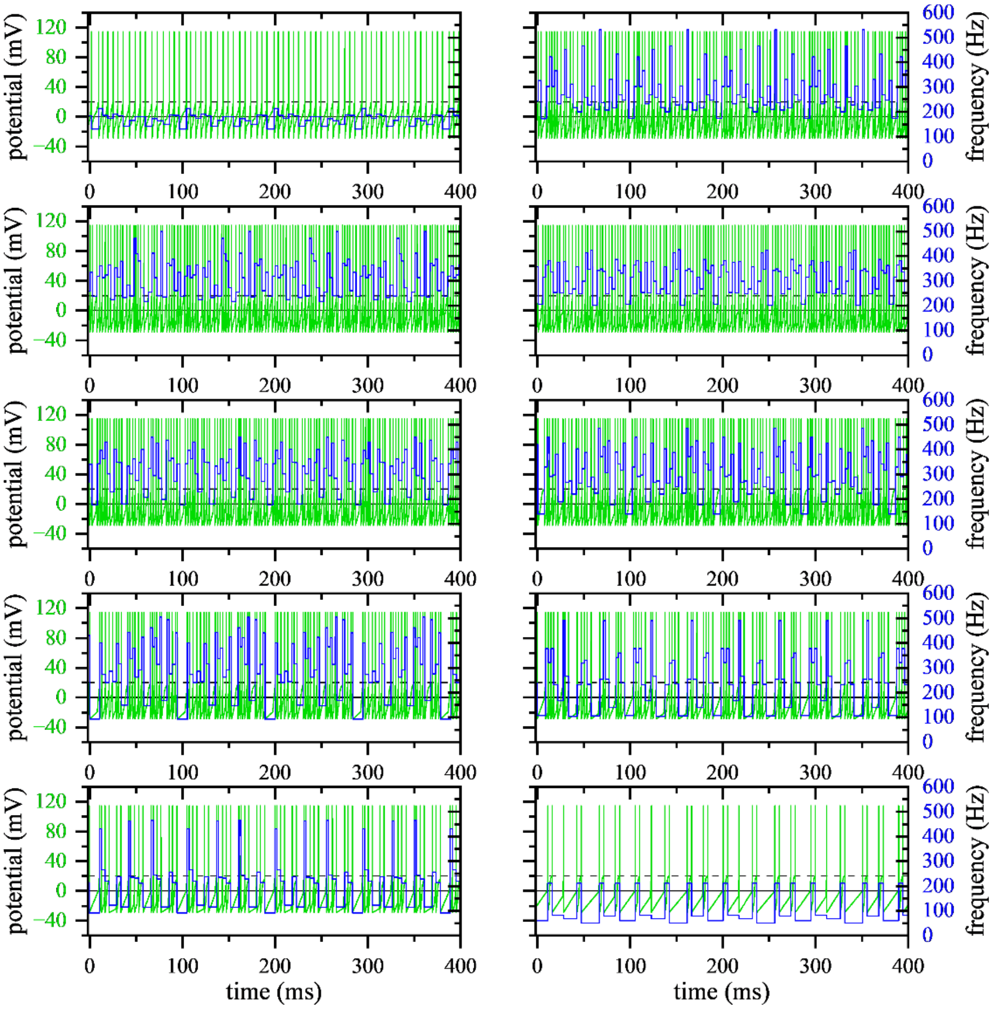

Fig. S15. Potential activity of the full positive feedback connected NS of 10 neurons. The initial condition is that the 3rd neuron triggers an action potential at 0.00 ms.

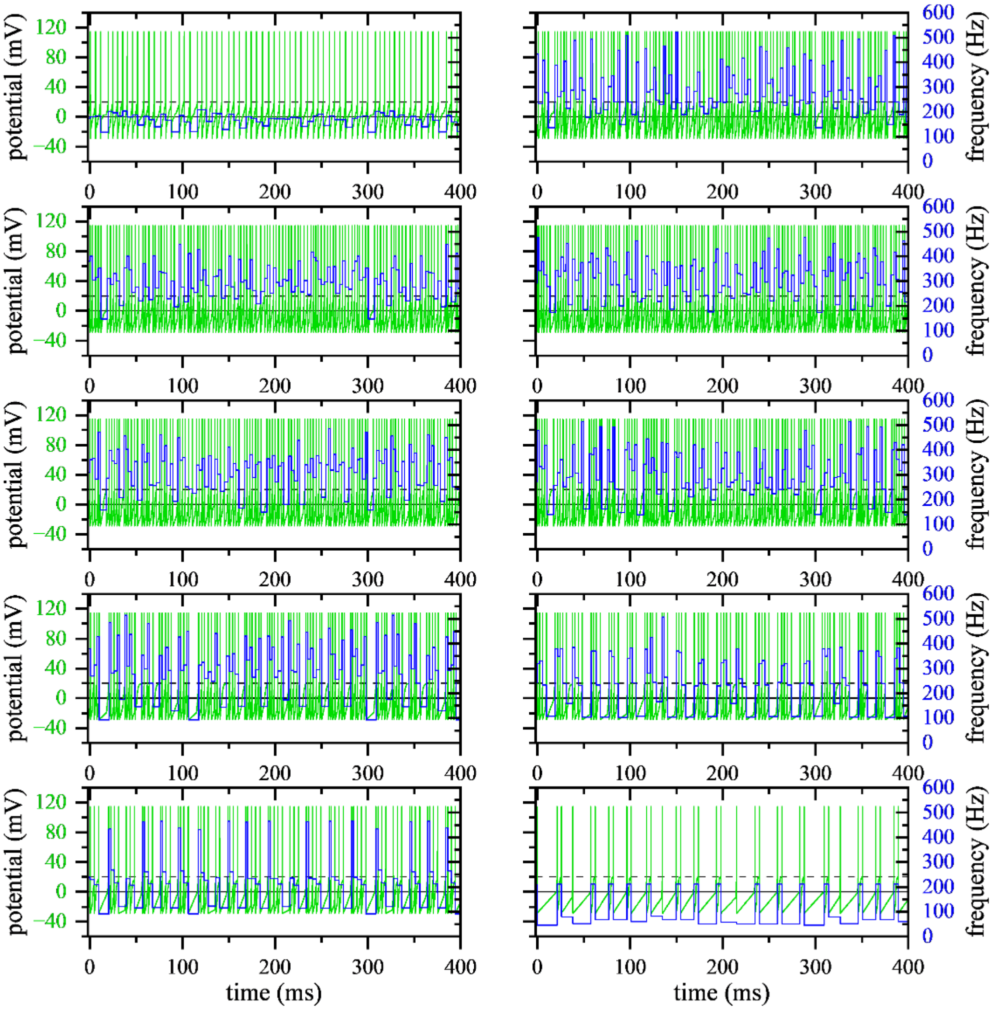

Fig. S16. Potential activity of the full positive feedback connected NS of 10 neurons. The initial condition is that the 4th neuron triggers an action potential at 0.00 ms.

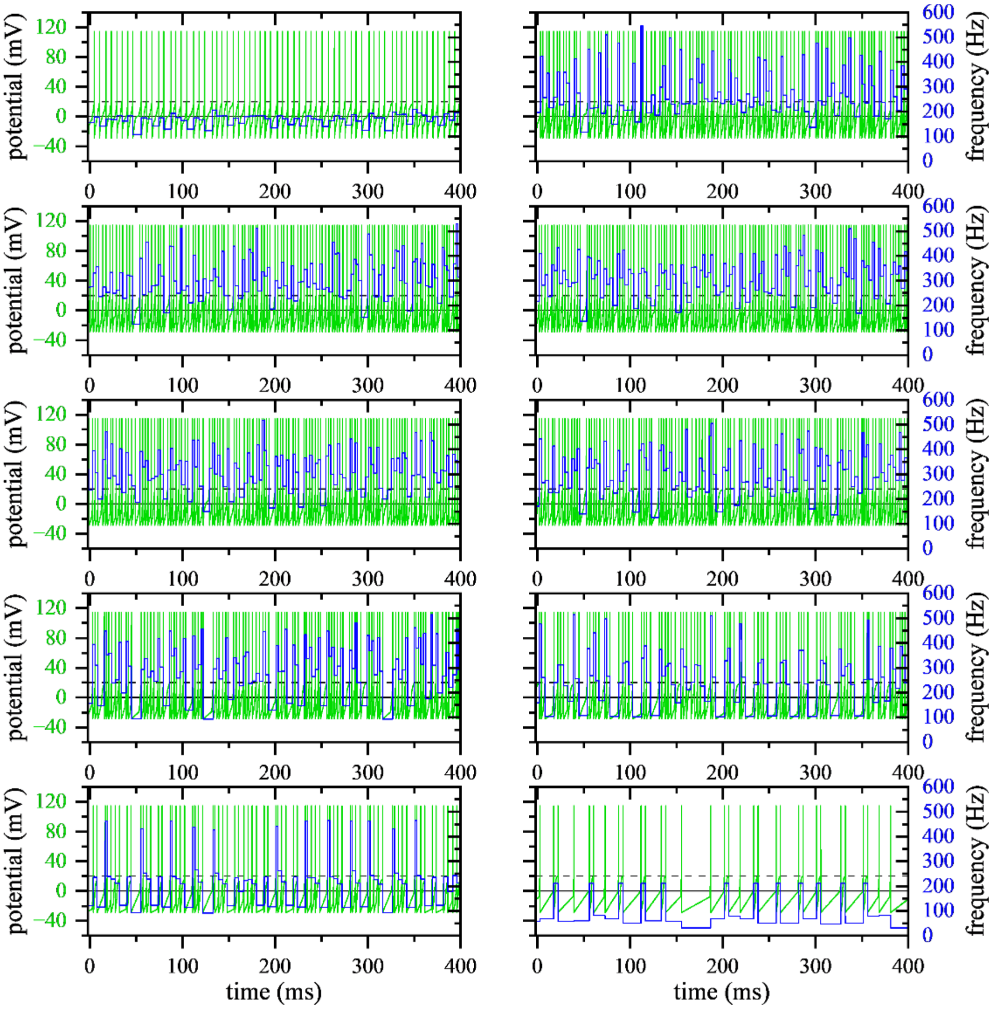

Fig. S17. Potential activity of the full positive feedback connected NS of 10 neurons. The initial condition is that the 5th neuron triggers an action potential at 0.00 ms.

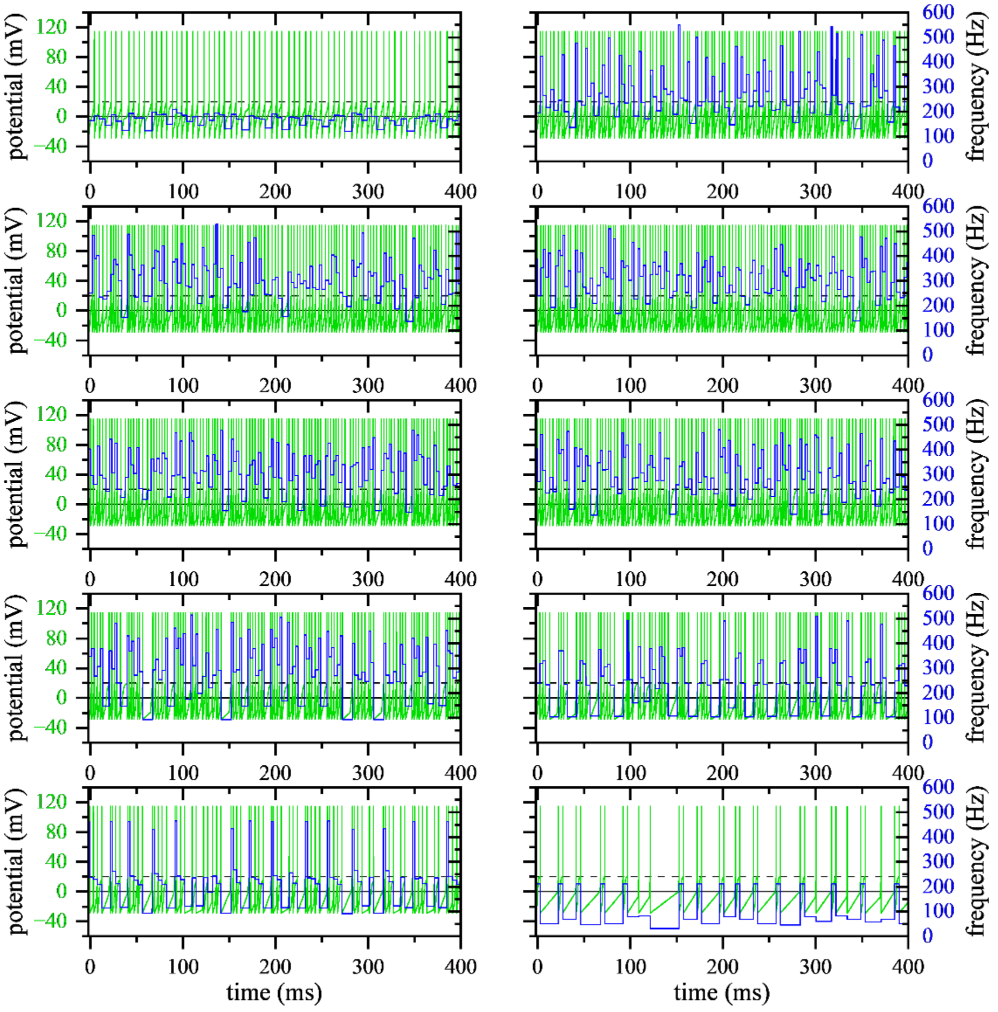

Fig. S18. Potential activity of the full positive feedback connected NS of 10 neurons. The initial condition is that the 6th neuron triggers an action potential at 0.00 ms.

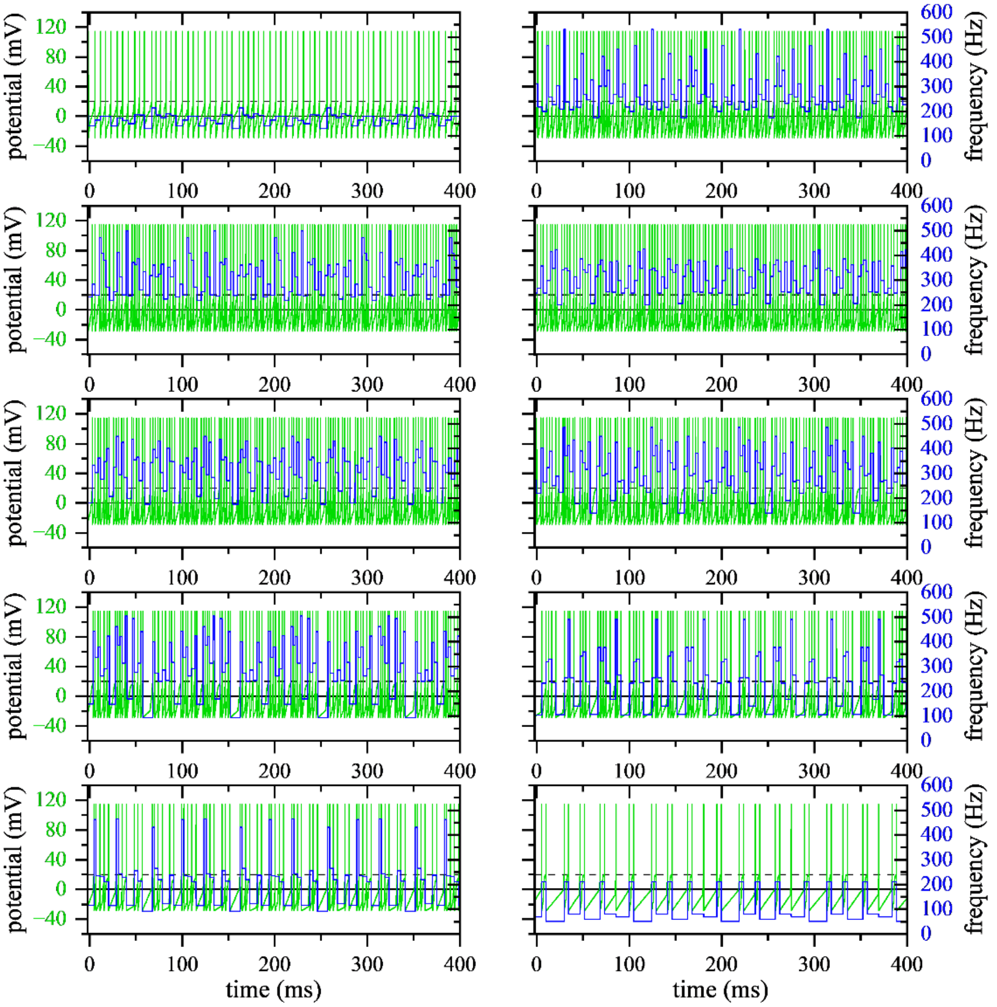

Fig. S19. Potential activity of the full positive feedback connected NS of 10 neurons. The initial condition is that the 7th neuron triggers an action potential at 0.00 ms.

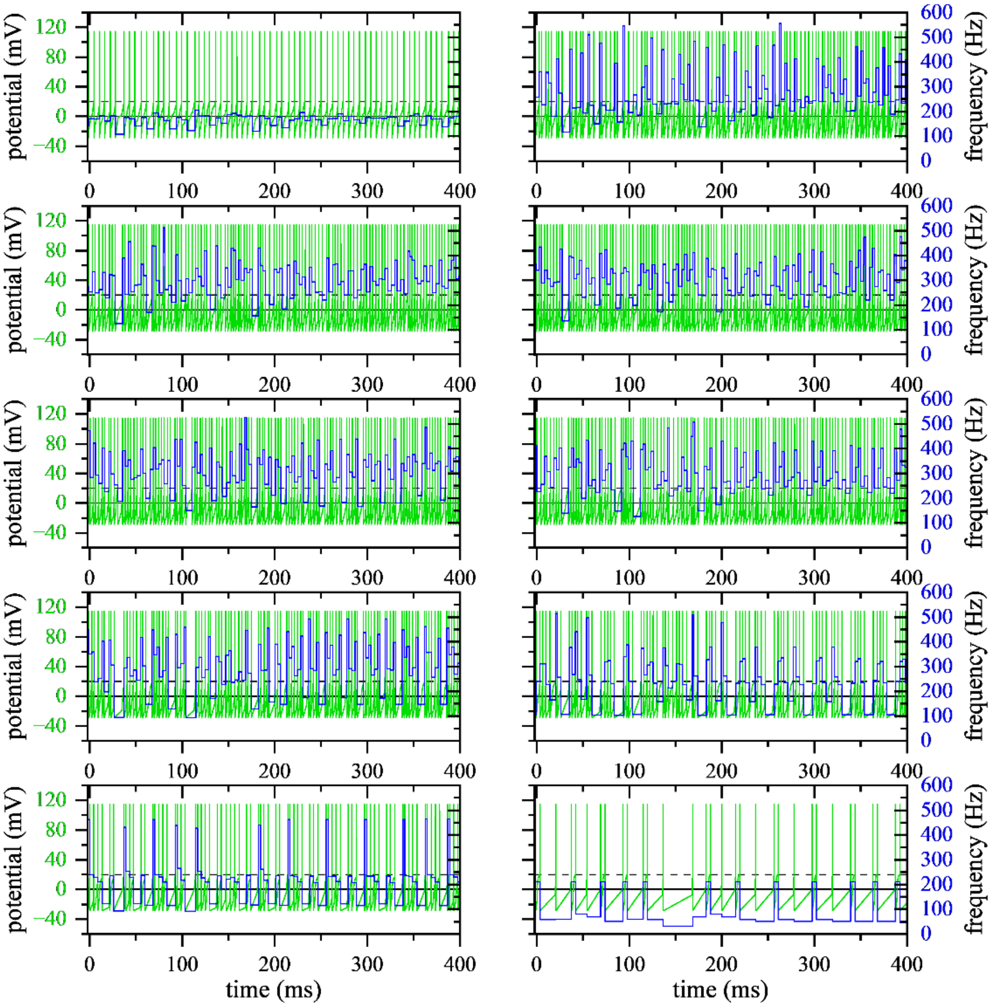

Fig. S20. Potential activity of the full positive feedback connected NS of 10 neurons. The initial condition is that the 8th neuron triggers an action potential at 0.00 ms.

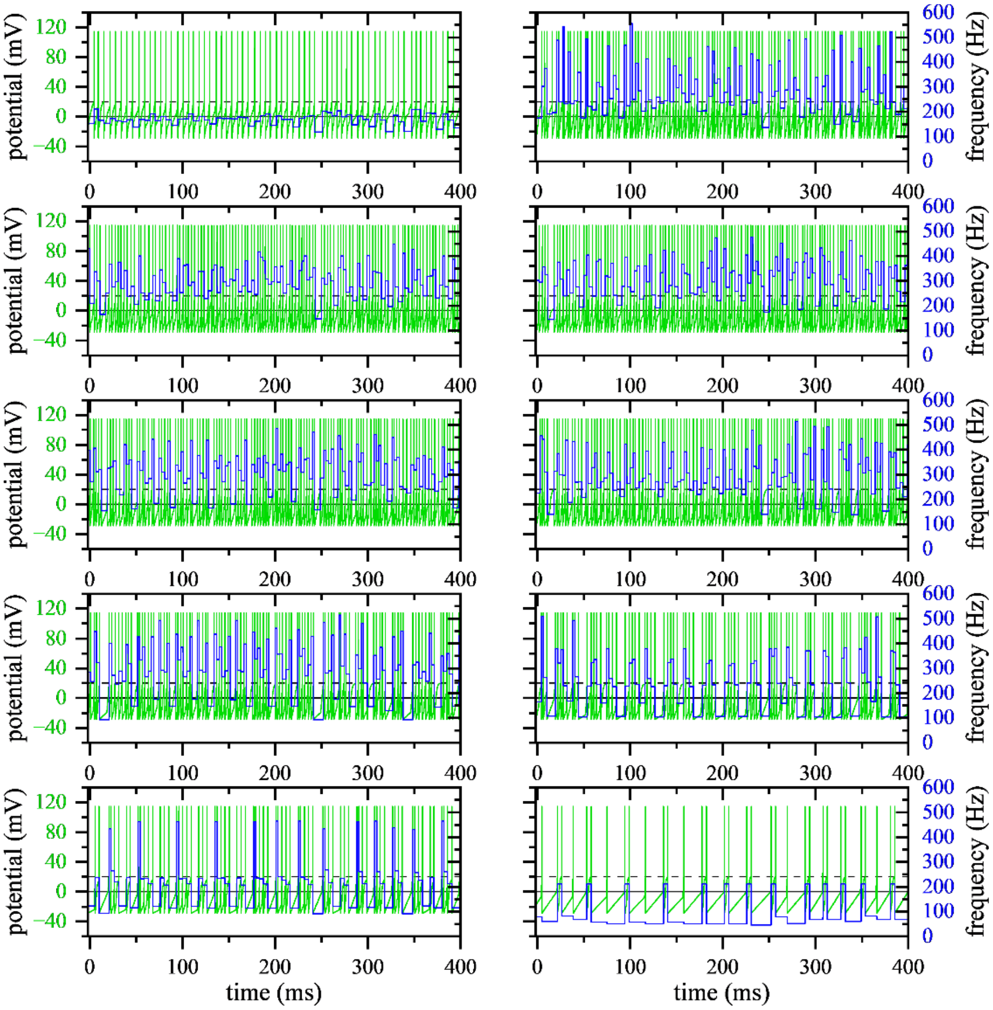

Fig. S21. Potential activity of the full positive feedback connected NS of 10 neurons. The initial condition is that the 9th neuron triggers an action potential at 0.00 ms.

Figure S22 shows the autocorrelation coefficients of the potential activity of the above full positive feedback connected NSs. With the change of initial condition, the full positive feedback connected NSs have a finite number of different periods. Most of the periods are 288.11 ms.

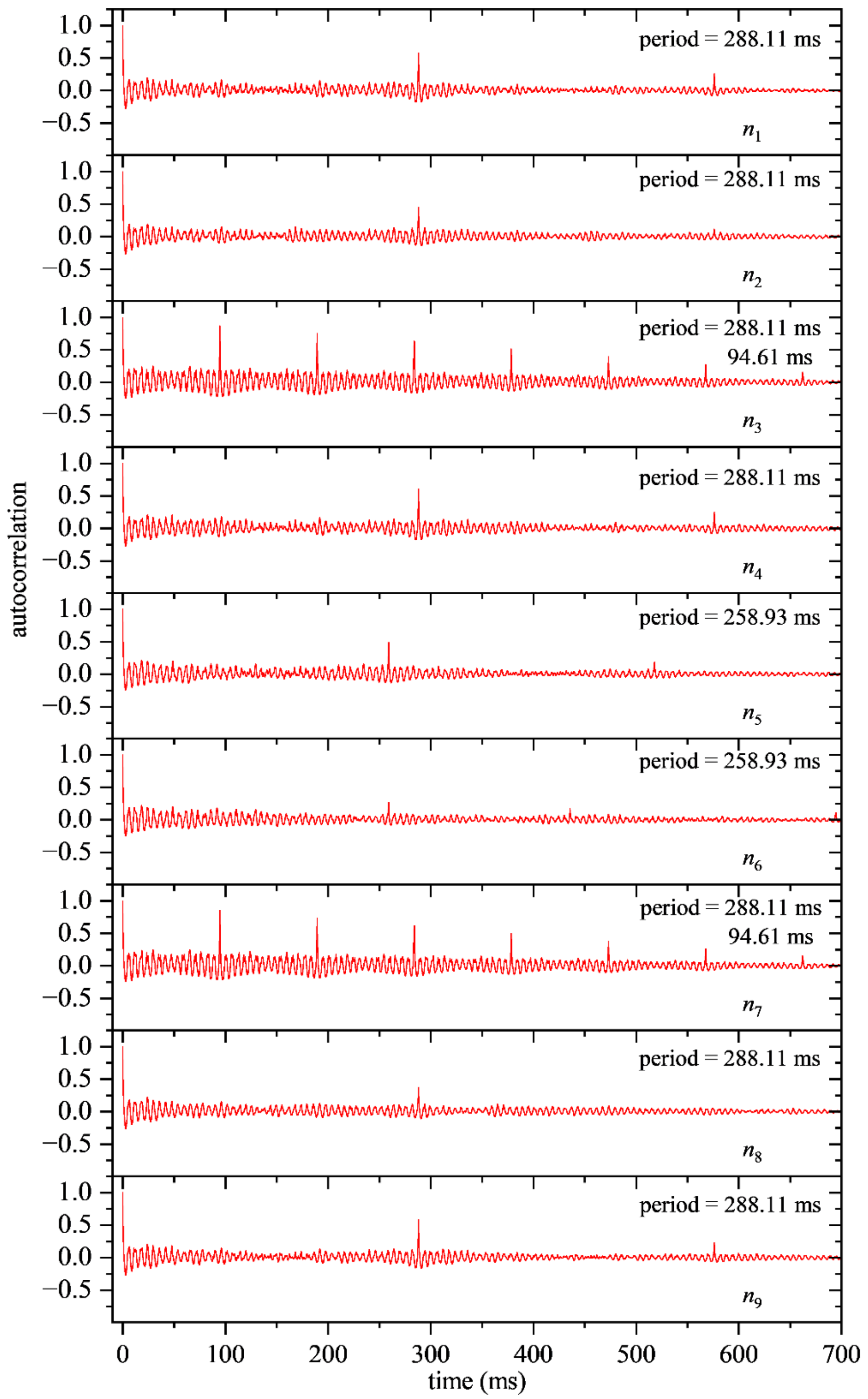

Fig. S22. The autocorrelation coefficient of the periodic potential waveform of full positive feedback connected NS of 10 neurons for different initial conditions. The initial condition n_i is that the neuron i triggers an action potential at 0.00 ms.

Note 5 Electrophysiological simulations of large-scale neural sphere (NS)

A fractal NS with 3600 neurons is used for the simulation. The connection structure of this large-scale fractal NS is shown in Fig. S23. It is composed of a large number of local connections and a small number of long-distance connections. The initial condition for the simulation of the fractal NS is that neuron 1 in each 5-neuron NS triggers an action potential at 0 ms.

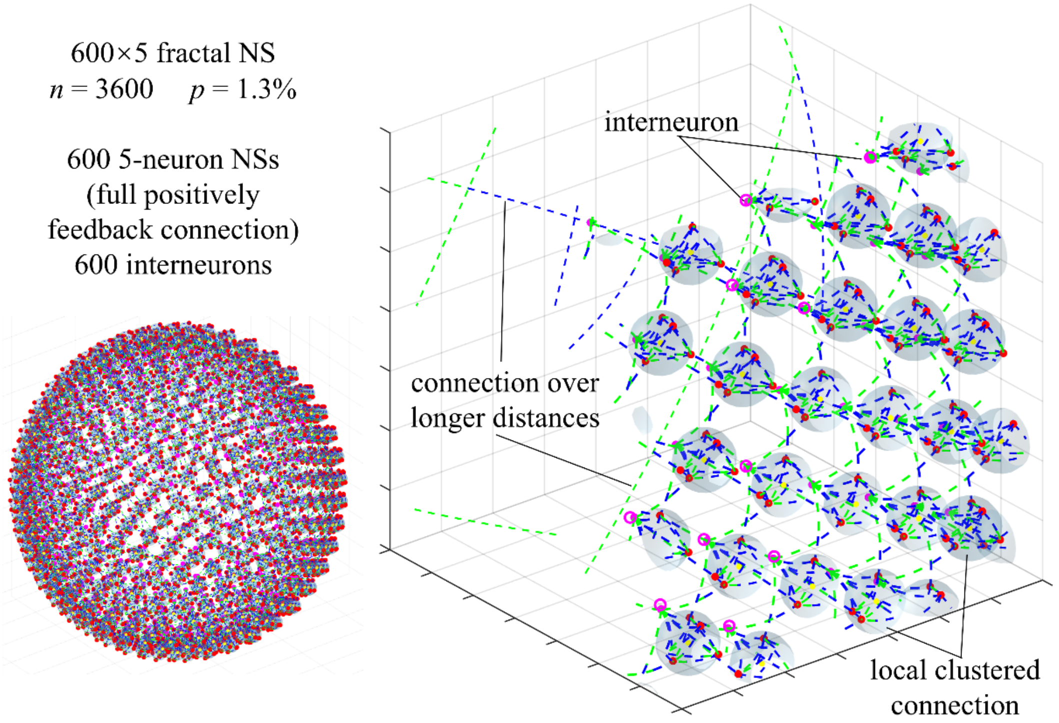

Fig. S23. The schematic diagram of the large-scale fractal NS composed of 3600 neurons. The red dots are somas. The pink hollow dots are interneuron somas. The green dashed line is the dendrite. The blue dotted line is the axon.

In such a massive computational task of electrophysiology, the consumption of computing power increases exponentially with the increase in the number of neurons. The current computational power is insufficient to support the electrobiological computations of an NS system with more than 100 neurons (or 3000 synapses). Fortunately, the NS with periodic structure and the same initial conditions as shown in Fig. S23 can be calculated through an indirect calculation method. In the simulation of fractal NS, each 5-neuron NS has the same electrophysiological characteristics, with only differences in the phase of the initial condition. Therefore, based on the simulation of the full initial conditions of the 5-neuron NS (Note 6), the

electrophysiological activity of a 5-neuron NS in the large-scale fractal NS can be simulated by recording the phase differences of the inputs from other interneurons.

The electrophysiological activity of one of the 5-neuron NSs in this fractal NS is shown in Fig. S24. Their electrophysiological activities are the permutation of a set of potential waveforms of the 5-neuron NS corresponding with different initial conditions on a time series. The permutation of different types of electrophysiological activities in small-scale NS (four types: briefly triggering a few action potentials, sustained short-period action potential activity, sustained long-period action potential activity, and sustained random potential activity) will have an impact on the entire electrophysiological activity. Since NS frequently receives new inputs, the case of briefly triggering a few action potentials will not occur. The combination of long-term and short-term potential activities will result in a longer-term potential activity. Random potential activity will reduce the periodicity of the entire electrophysiological activity. To sum up, in symmetrical large-scale fractal NS, the electrophysiological activities of neurons generally tend to exhibits ultra-long periodicity, while a small number of random waveforms are observed locally.

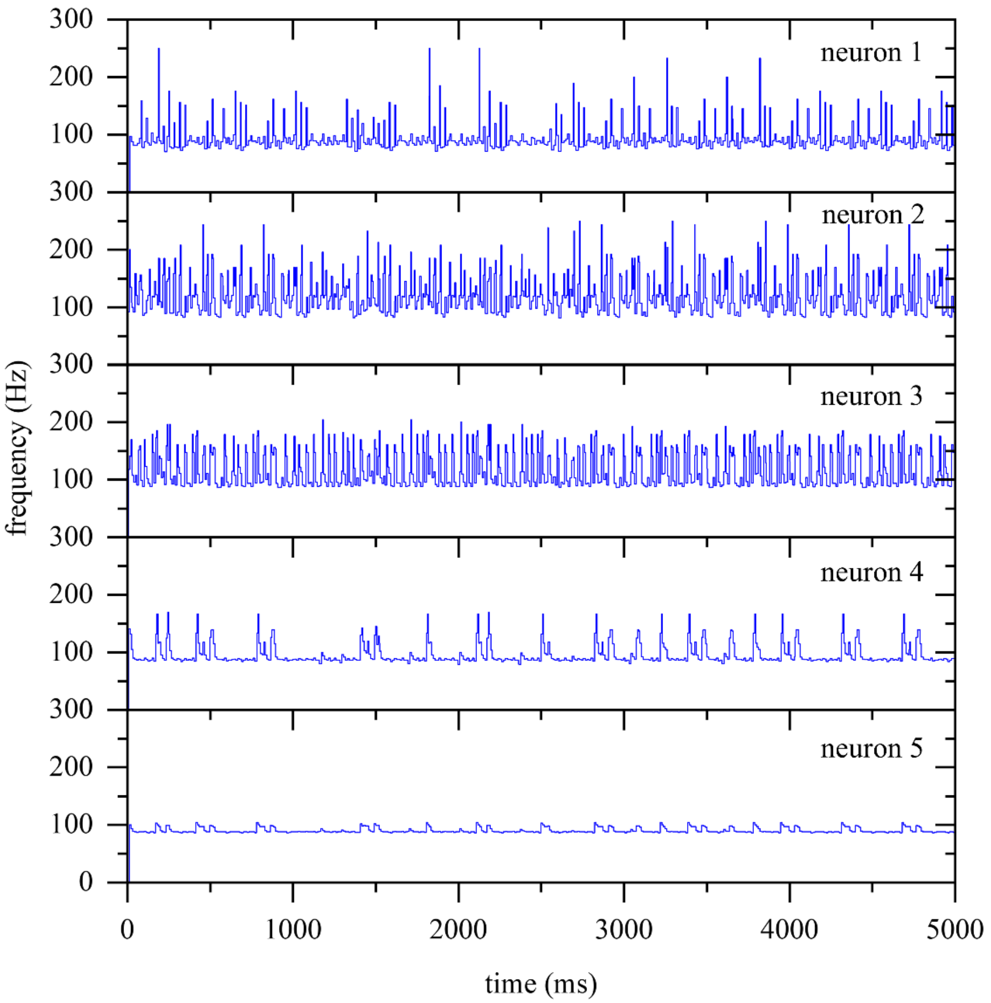

Fig. S24. The frequency of the electrophysiological activity of one of the 5-neuron NSs in the fractal NS with 3600 neurons.

Moreover, in large-scale NS with reduced symmetry, the electrophysiological activities of neurons exhibit a variety of characteristics. For example, a two-level fractal NS with 15600 neurons is constructed to analyze its electrophysiological characteristics. It is composed of 1200 small-scale 5-neuron NSs, 1200 small-scale 6-neuron NSs, and 2400 interneurons. The two types of small-scale NSs are randomly arranged on a large-scale NS to generate a small amount of asymmetry. In the regions with higher local symmetry, some small-scale NSs exhibit sustained long-period action potential activities such as Fig. S25. It only has a few peaks of autocorrelation coefficients in the long-term simulation. In the regions with lower local symmetry, some small-scale NSs exhibit sustained random potential activities such as Fig. S26. It don't have any peak of autocorrelation coefficients in the long-term simulation. Other small-scale NSs exhibit

sustained short-period action potential activities such as Fig. S27. It has dense peaks of autocorrelation coefficients in the long-term simulation. The electrophysiological activities of neurons in large-scale fractal NS are pieced together by those of neurons in lower-level small-scale NS. Therefore, large-scale NS exhibit similar electrophysiological activity characteristics to those of small-scale NS. It only differs in terms of the time scale. Based on the above numerical experiments, the electrophysiological activity of a neuron in brain is the permutation of a set of potential waveforms corresponding with different initial conditions on a time series.

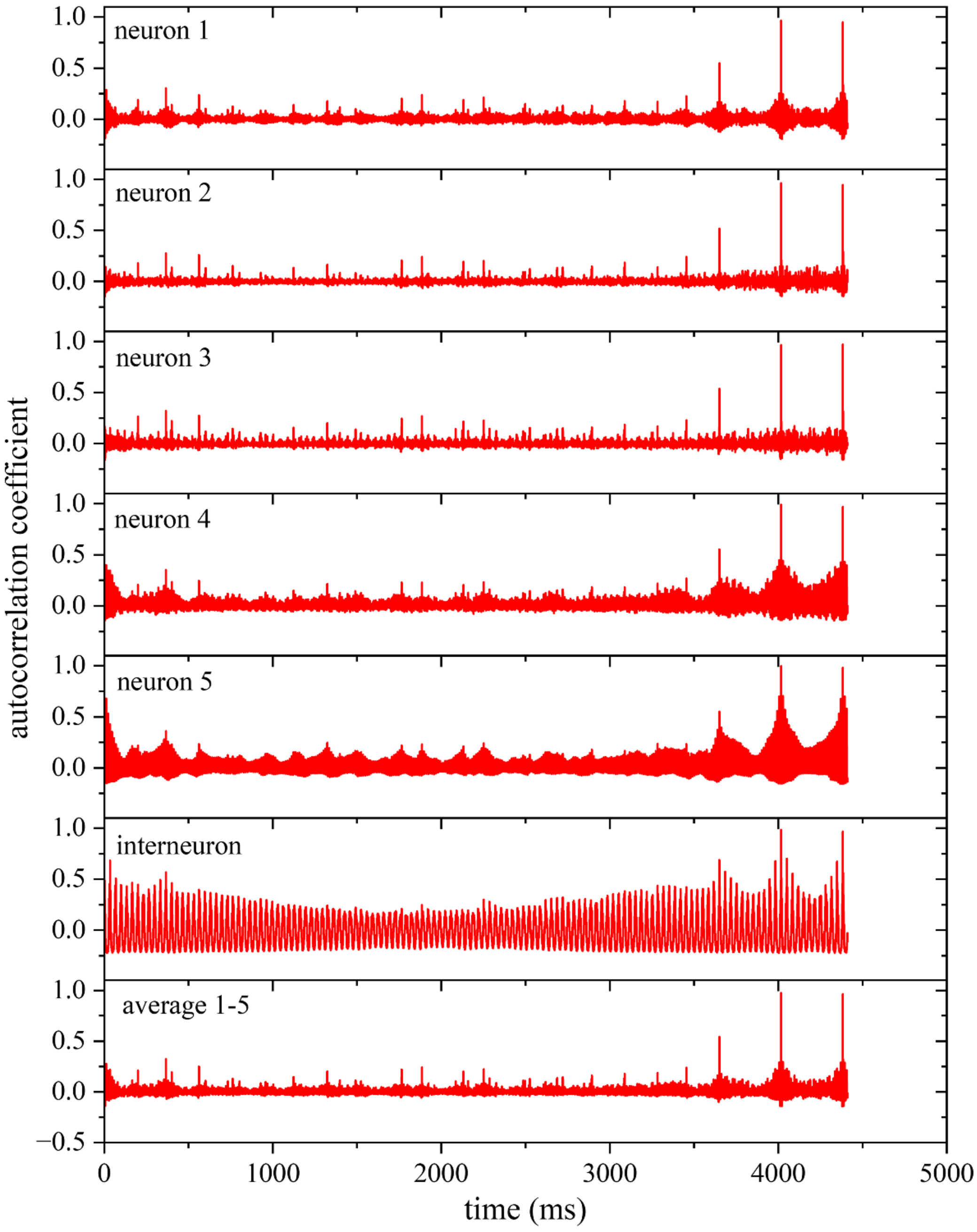

Fig. S25. The autocorrelation coefficient of the electrophysiological activity of one of the 5-neuron NSs in the fractal NS with 15600 neurons. It indicates that the electrophysiological activity of this NS exhibits sustained long-period action potential activity.

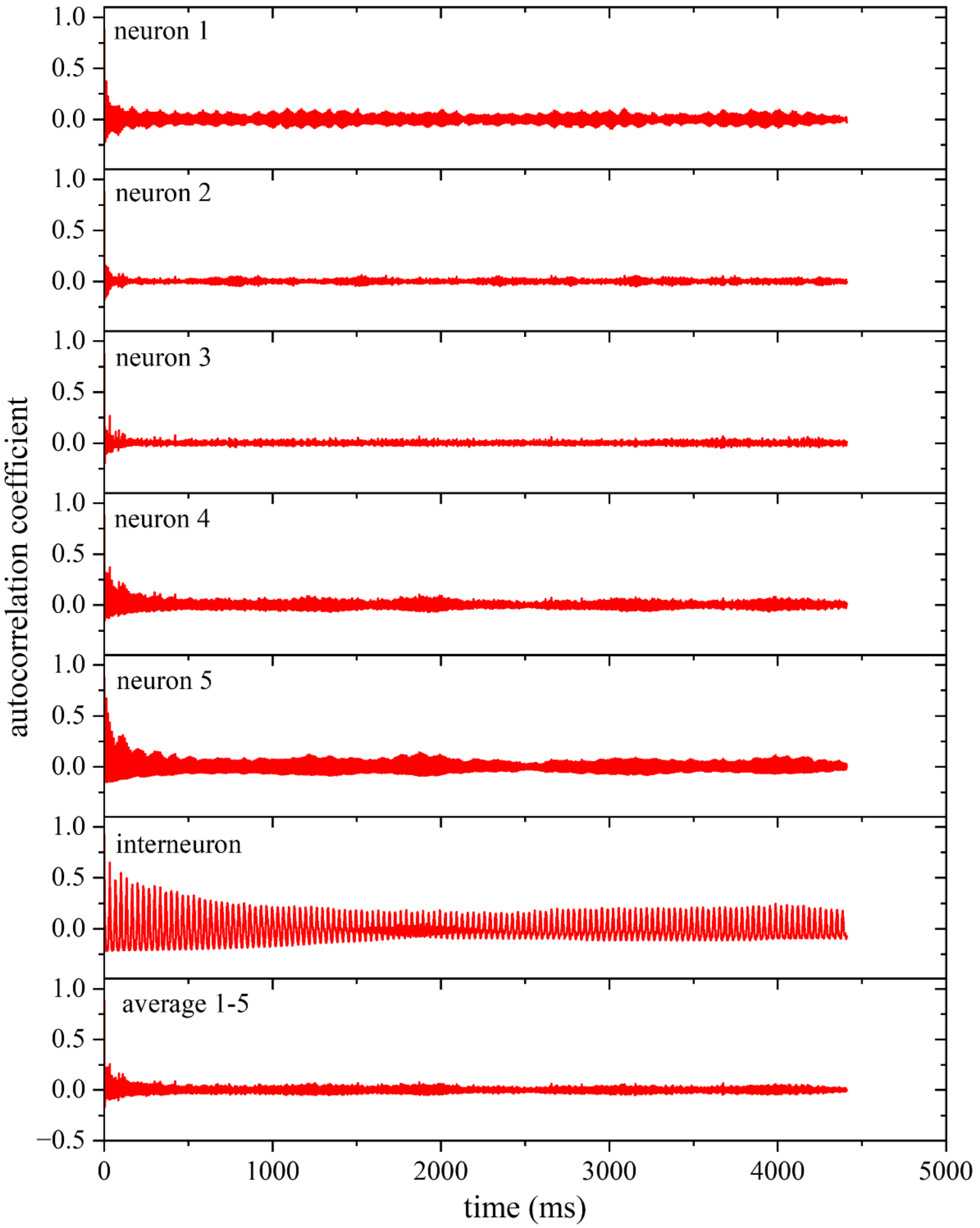

Fig. S26. The autocorrelation coefficient of the electrophysiological activity of one of the 5-neuron NSs in the fractal NS with 15600 neurons. It indicates that the electrophysiological activity of this NS exhibits sustained random potential activity.

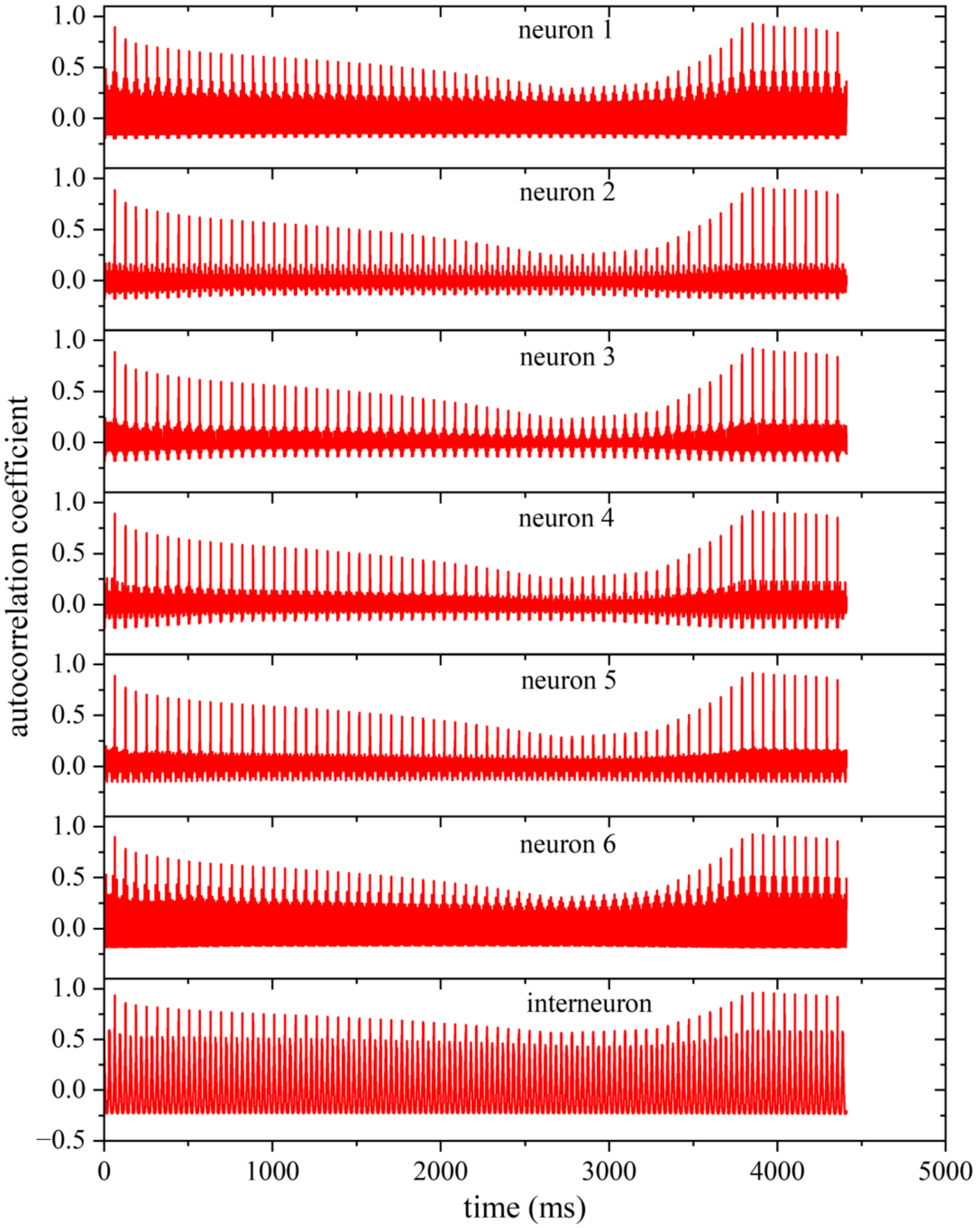

Fig. S27. The autocorrelation coefficient of the electrophysiological activity of one of the 6-neuron NSs in the fractal NS with 15600 neurons. It indicates that the electrophysiological activity of this NS exhibits sustained short-period action potential activity.

Note 6 Electrophysiological simulations of full initial conditions for neural sphere (NS) with five neurons

To characterize the information features of NS, the electrophysiological activities of full positive feedback NS with $n = 5$ and $q = 1$ in full initial conditions are simulated. The minimum difference between the elements of the initial condition matrix is 0.1 ms. Their upper limits are all less than 5 ms. These initial conditions cover all situations that guarantee that the NS generates sustained potential activity. For each initial condition, membrane potential activity of NS was simulated for up to 50 ms at the time resolution of 0.01 ms. A total of 221,551 groups of such simulations were performed. The connection matrix of the full positive feedback NS with 5 neurons is shown in Table S4. The original Data of above simulation are stored in Data S1.

Data S1. (original data.rar) The "original data.rar" file contains two folders, "input" and "output". In the file of "input", "1.csv" to "5.csv" are the information of five neurons in turn. "matrices.csv" contains information about all the initial condition matrices. "synapse_connection_matrix_1.csv" and "synapse_connection_matrix_2.csv" contains information about the synaptic connections between neurons. In the file of "output", "membrane_potential_activity_1.csv" to "membrane_potential_activity_221551.csv" are the simulated information of 221551 groups of membrane potential activity in turn. Please refer to "readme.txt" for details.

Index of connection	Index of neuron (axon)	Index of synapse (axon)	Index of neuron (dendrite)	Index of synapse (dendrite)	stimulus intensity coefficient k_v
1	1	5	2	1	400
2	2	5	3	1	400
3	2	6	1	2	80
4	3	4	4	1	400
5	3	5	2	2	160
6	3	6	1	3	80
7	4	3	5	1	400
8	4	4	3	2	240
9	4	5	2	3	160
10	4	6	1	4	80
11	5	3	4	2	320

12	5	4	3	3	240
13	5	5	2	4	160

Table S4. The connection matrix of the full positive feedback NS with 5 neurons.

By calculating the first peak of the autocorrelation coefficient function of the membrane potential activity time series for all electrophysiological simulations, two frequency histograms of roughly lognormal distribution in Fig. S28 are obtained. The autocorrelation coefficient close to 1 or 0 indicates that the potential activity has stronger periodicity or chaotic property, respectively.

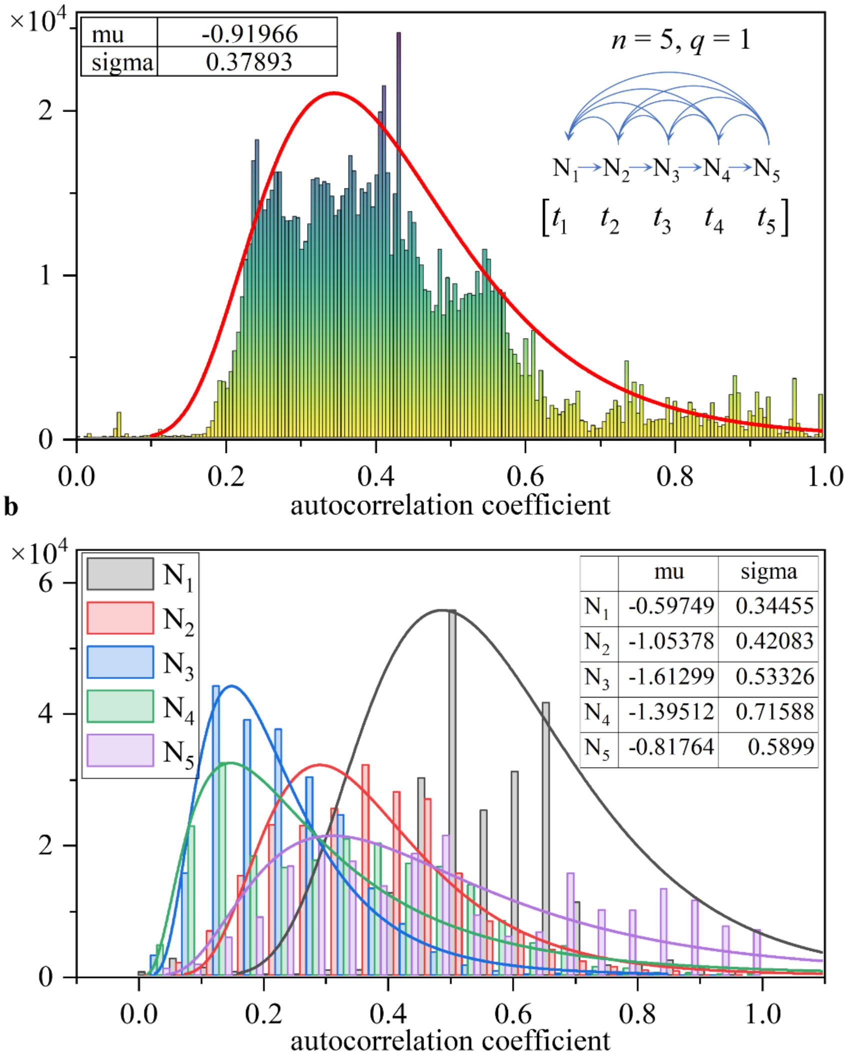

Fig. S28. The frequency histogram of the autocorrelation coefficient under all initial conditions. (**a**) The frequency histogram of the autocorrelation coefficient of all neuronal potential activities under all initial conditions. The simulation object is the full positive feedback NS of five neurons (n = 5). The initial condition is that each neuron triggers an action potential at

most once ($q = 1$). The connection mode of NS and the initial condition matrix are given in the upper right corner of the figure. (**b**) The frequency histogram of the autocorrelation coefficient of the potential activity of each neuron under all initial conditions.

In the five-dimensional space spanned by the initial conditions, similar autocorrelation coefficients exhibit distinct clustering characteristics on ten plane projections (Fig. S29a). It indicates that the initial conditions for the same type of potential activity are similar. Besides, by calculating the correlation coefficient of the potential activities generated by 1000 groups of similar initial conditions from the simulation data (Fig. S29b), it is found that most of the potential activities have weak correlation with each other (< 0.5). The comprehensive correlation analysis is shown in Fig. S30-34. It means that NS can well distinguish initial conditions with slight differences.

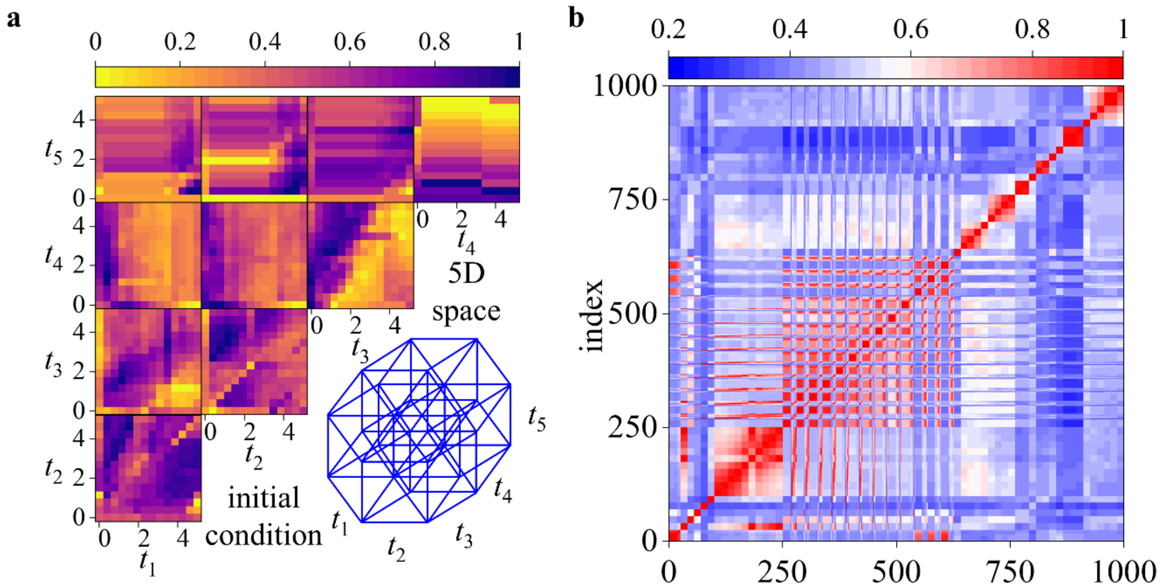

Fig. S29. Correlation coefficient heat map of Electrophysiological simulation of NS. (**a**) All planar projections of the autocorrelation coefficient heat map in the five-dimensional space spanned by the initial condition matrix. (**b**) Heat map of potential activity correlation coefficients under 1000 similar initial conditions.

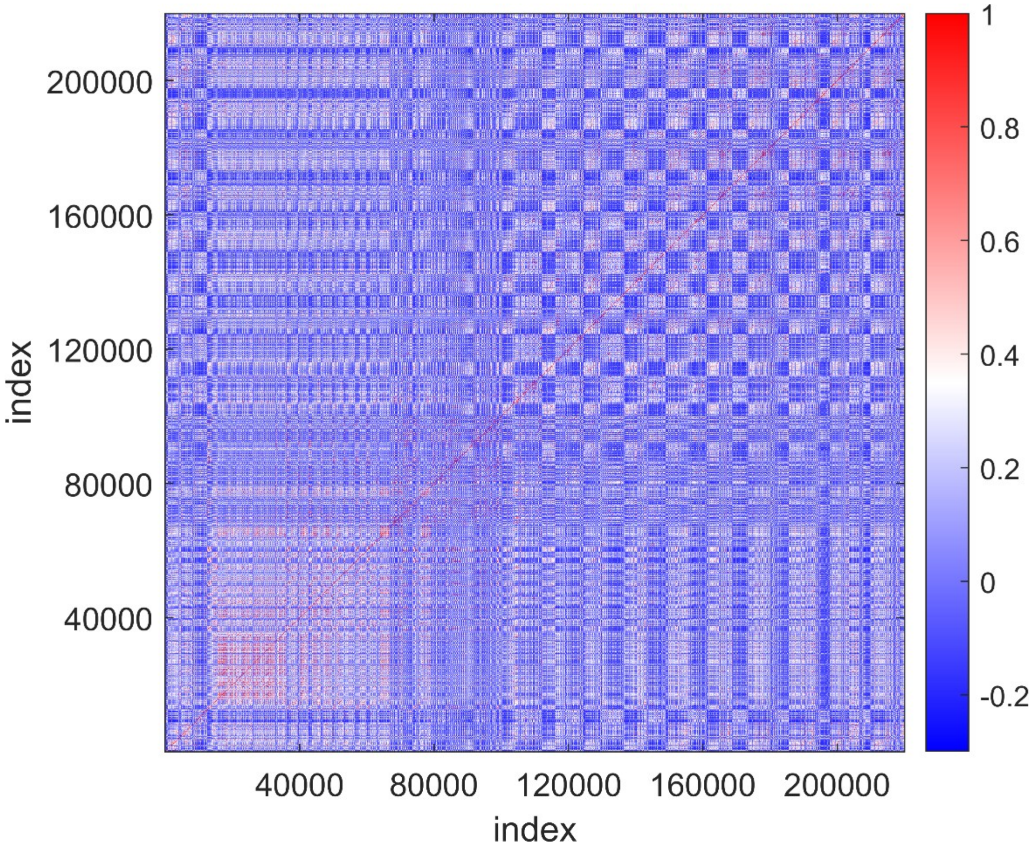

Fig. S30. Heat map of potential activity correlation coefficients for all initial conditions in neuron 1.

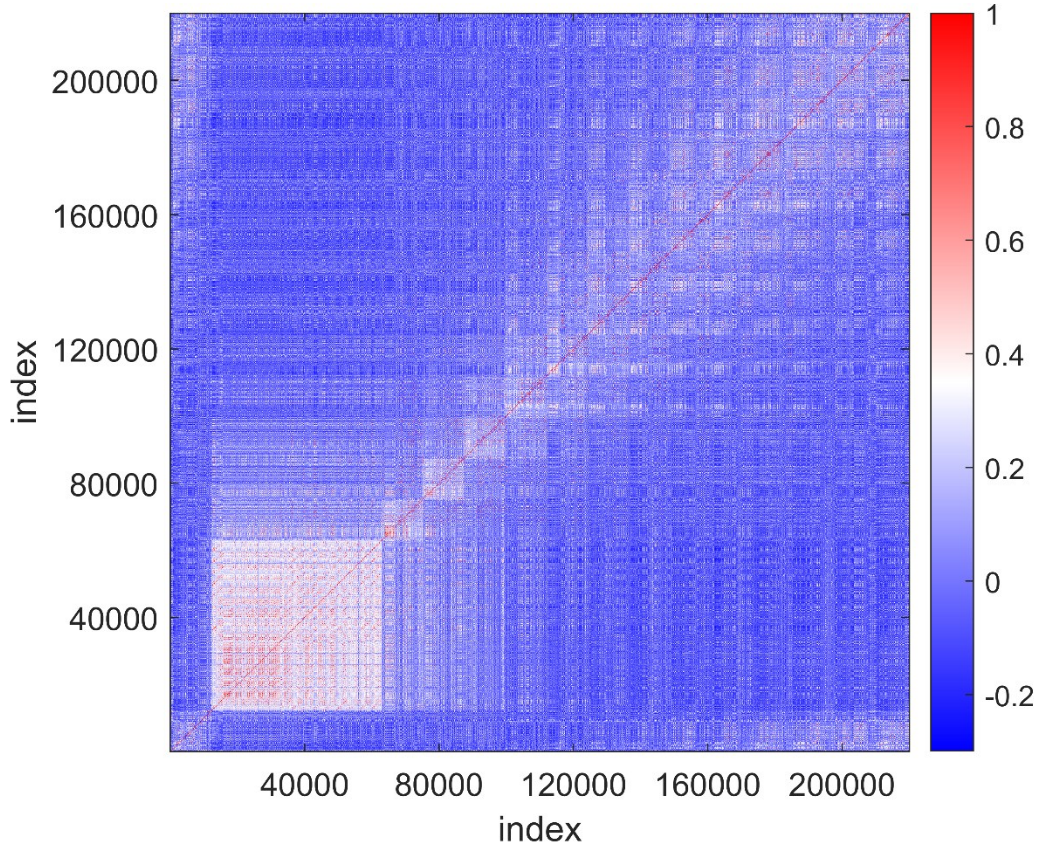

Fig. S31. Heat map of potential activity correlation coefficients for all initial conditions in neuron 2.

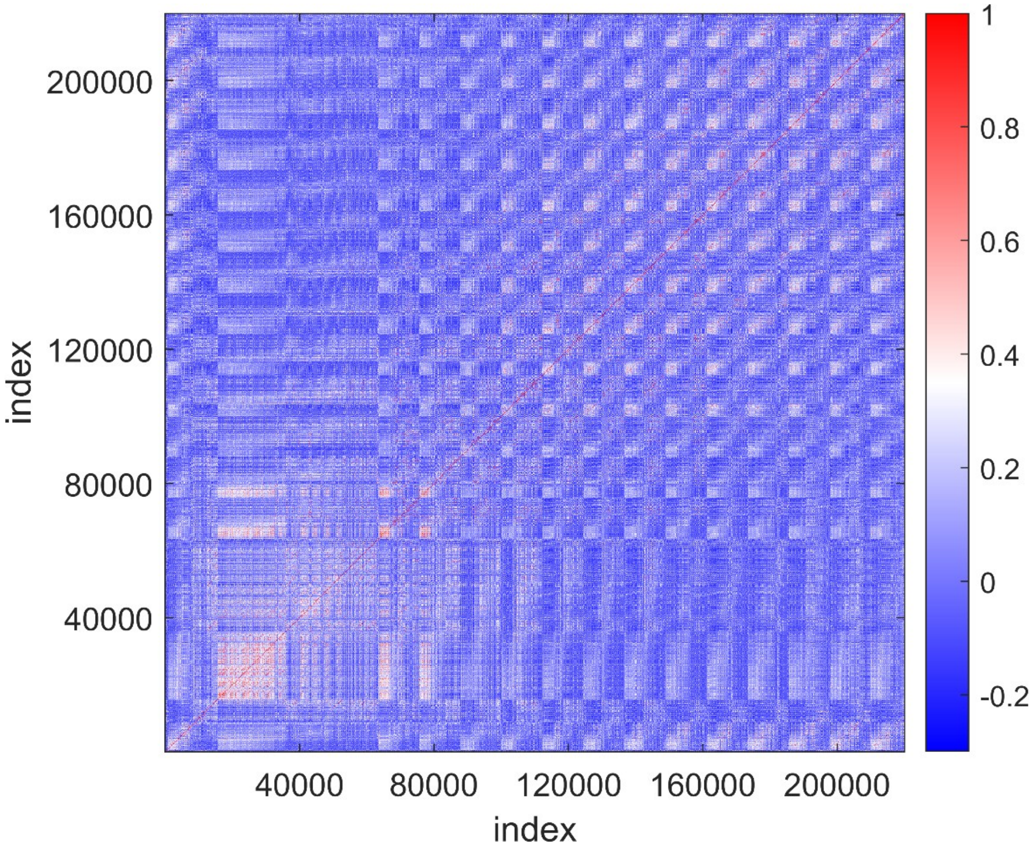

Fig. S32. Heat map of potential activity correlation coefficients for all initial conditions in neuron 3.

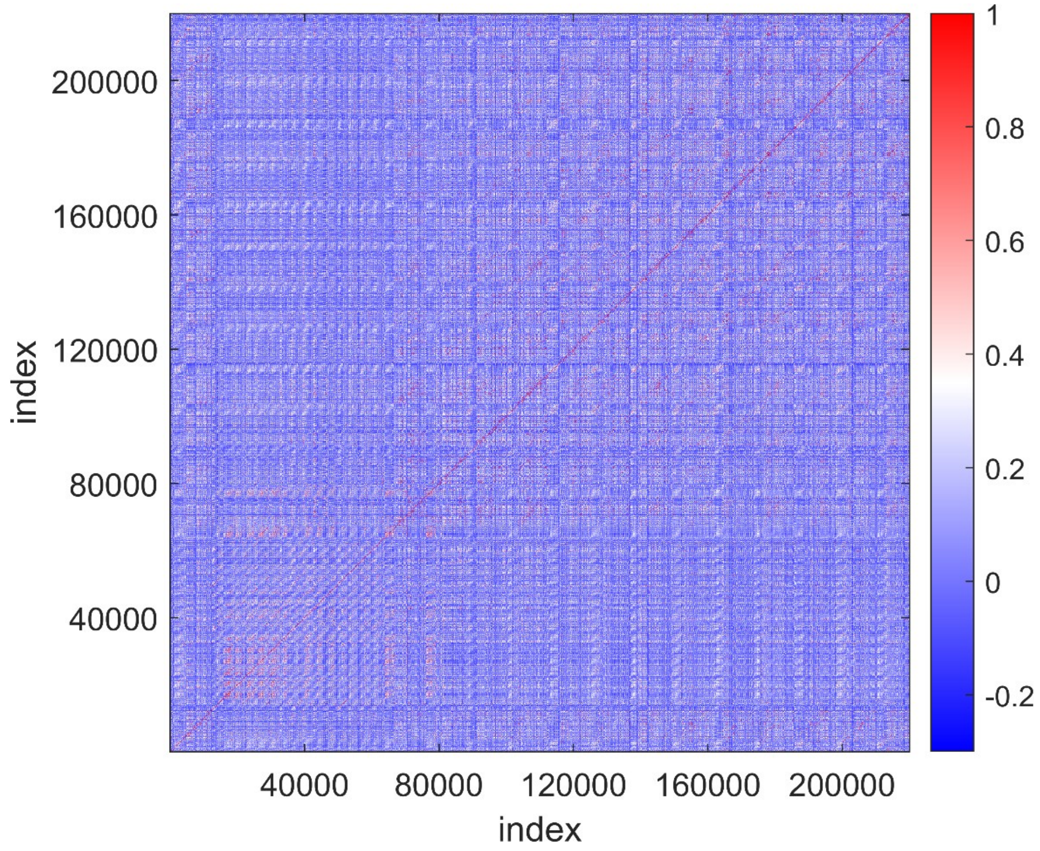

Fig. S33. Heat map of potential activity correlation coefficients for all initial conditions in neuron 4.

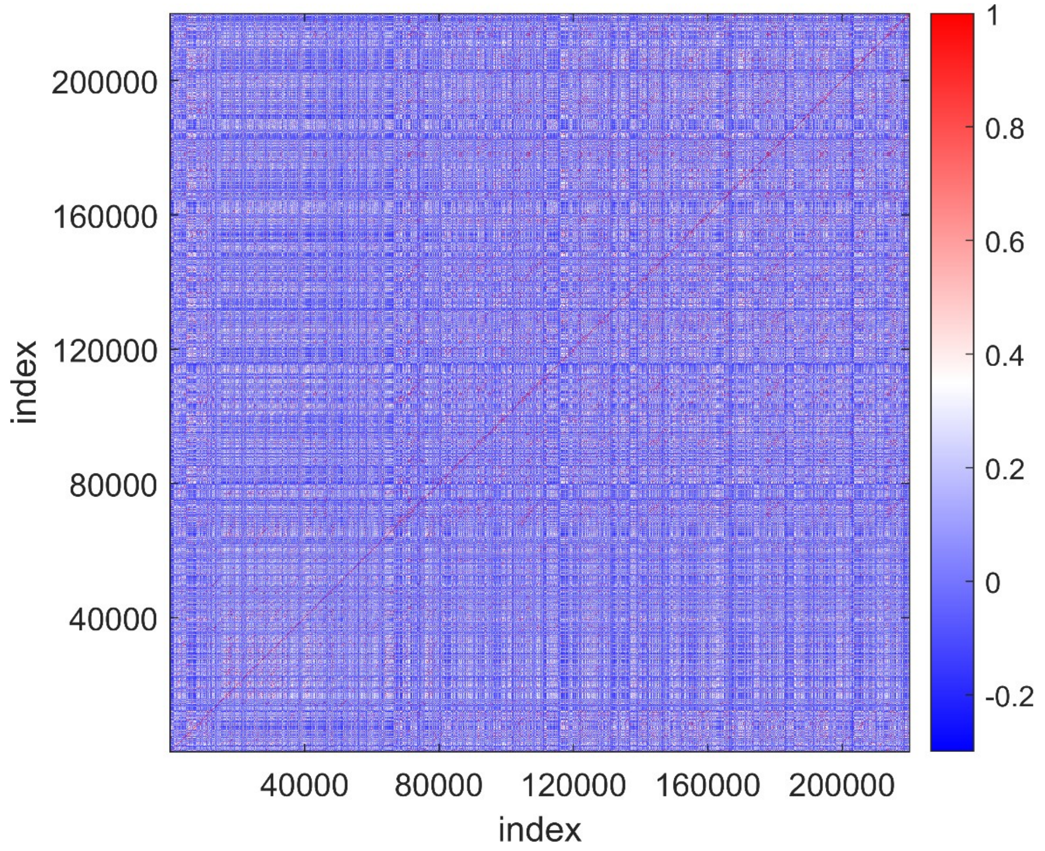

Fig. S34. Heat map of potential activity correlation coefficients for all initial conditions in neuron 5.

Note 7 Simplified neural sphere (NS) dynamical system

In order to explain the above mathematical principle of the information features of NS, this letter constructed a simplified NS dynamical system to analyze how the initial conditions drive the potential activity of NS. As shown in Fig. S35a, the phase diagram of one of the neurons is a beautiful fractal graph. After being infinitely magnified, each part has a similar structure. An attractor corresponds to a period potential activity. The point of convergence to the attractor corresponds to convergence to period potential activity. Randomly being alternately attracted by several attractors corresponds to random potential activity. The proportion of area in the phase portrait of the above three cases is consistent well with the simulated data in Fig. 3a. Taking the initial state of each neuron as a dimension, the NS dynamical system will constitute an *n*-dimensional complex space (Fig. S35b). The periodic potential waveform is a high-dimensional strange attractor in this space. The points that periodically flow on the high-dimensional attractor projected onto a single dimension will exhibit a periodic complex oscillation. If attractor projected onto one of the neurons, it will represent a set of initial conditions corresponding to the current periodic electrophysiological activity.

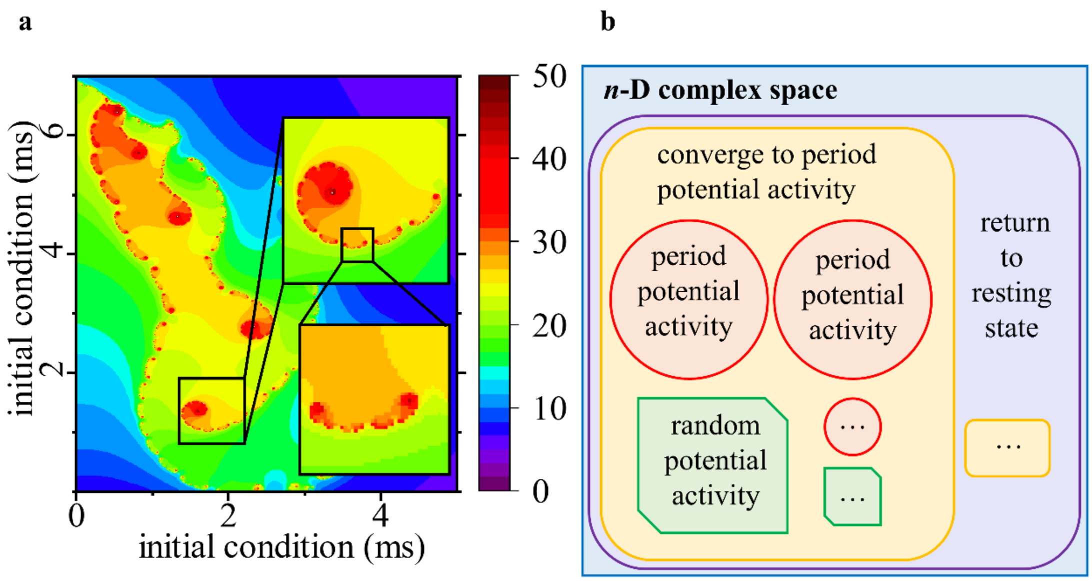

Fig. S35. Phase space of simplified NS dynamical system. (**a**) One of the two-dimensional local projection of the phase portrait for simplified NS dynamical system. The horizontal and vertical coordinates respectively correspond to the initial conditions of the two neurons in NS. The color graph represents the number of iterations. (**b**) Schematic diagram of the positions and containment relationships of four typical potential activities in the *n*-dimensional complex space spanned by the initial condition and potential state.

By changing the control parameters a_i', K_{ij}' and Φ_i' of the simplified NS dynamical system, different Phase portraits with beautiful fractal patterns can be generated. Figures S36-S40 show the phase portraits of the full positive feedback NS with $n = 5$ for some control parameters, where a_i' is 1.00 ~ 1.00+0.25i,

$$\boldsymbol{K}_{\mathrm{V}}' = \begin{bmatrix} 0 & 0.4+0.01i & 0.3+0.01i & 0.2+0.01i & 0.1+0.01i \\ 0.5+0.01i & 0 & 0.4+0.01i & 0.3+0.01i & 0.2+0.01i \\ 0 & 0.5+0.01i & 0 & 0.4+0.01i & 0.3+0.01i \\ 0 & 0 & 0.5+0.01i & 0 & 0.4+0.01i \\ 0 & 0 & 0 & 0.5+0.01i & 0 \end{bmatrix},$$

and Φ_i' is 0.02 ~ 0.2. When plotting the phase portrait of neuron j, the rest of the z_i ($i \neq j$) are all 0. The detailed visualization of the evolution process of the simplified NS dynamical system is presented in Movie S2.

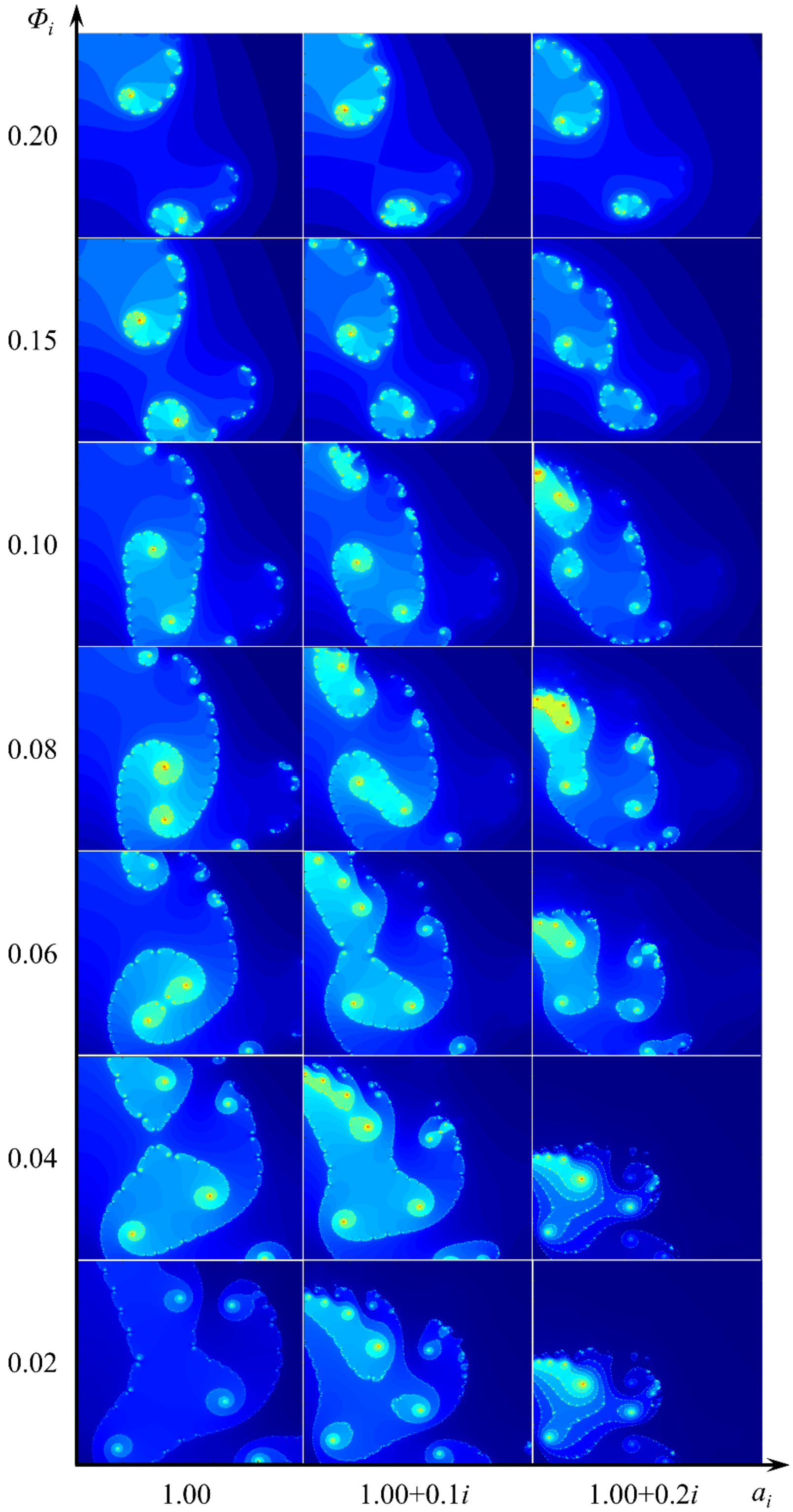

Fig. S36. Phase portraits of the simplified NS dynamical system with different control parameters for neuron 1. The horizontal and vertical coordinates correspond respectively to the real part and the imaginary part of the variable z. From blue to yellow and then to red, the number of iterations increased successively. The range of the coordinates of each phase portrait are all [0 5 ms].

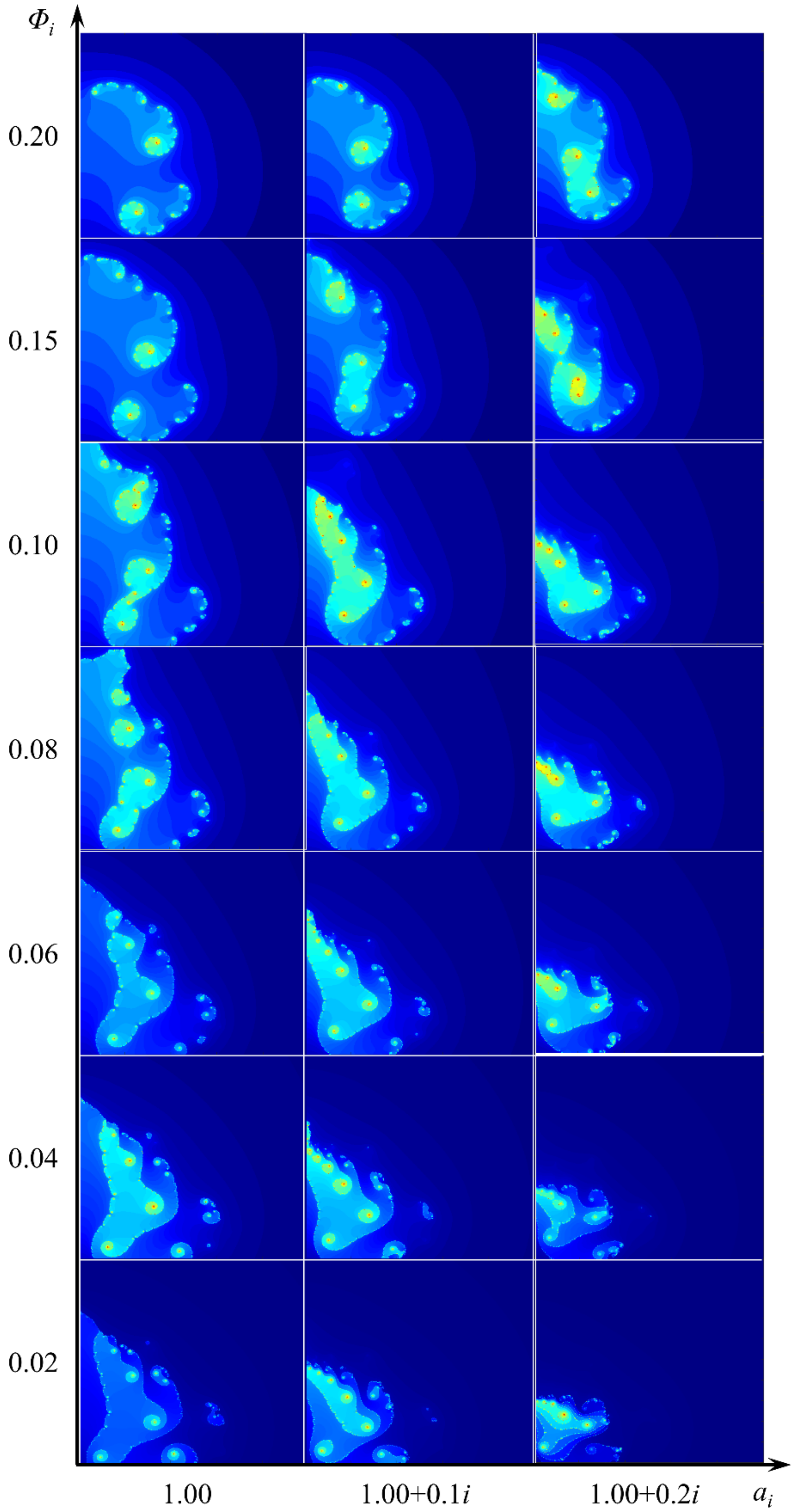

Fig. S37. Phase portraits of the simplified NS dynamical system with different control parameters for neuron 2. The horizontal and vertical coordinates correspond respectively to the real part and the imaginary part of the variable z. From blue to yellow and then to red, the number of iterations increased successively. The range of the coordinates of each phase portrait are all [0 5 ms].

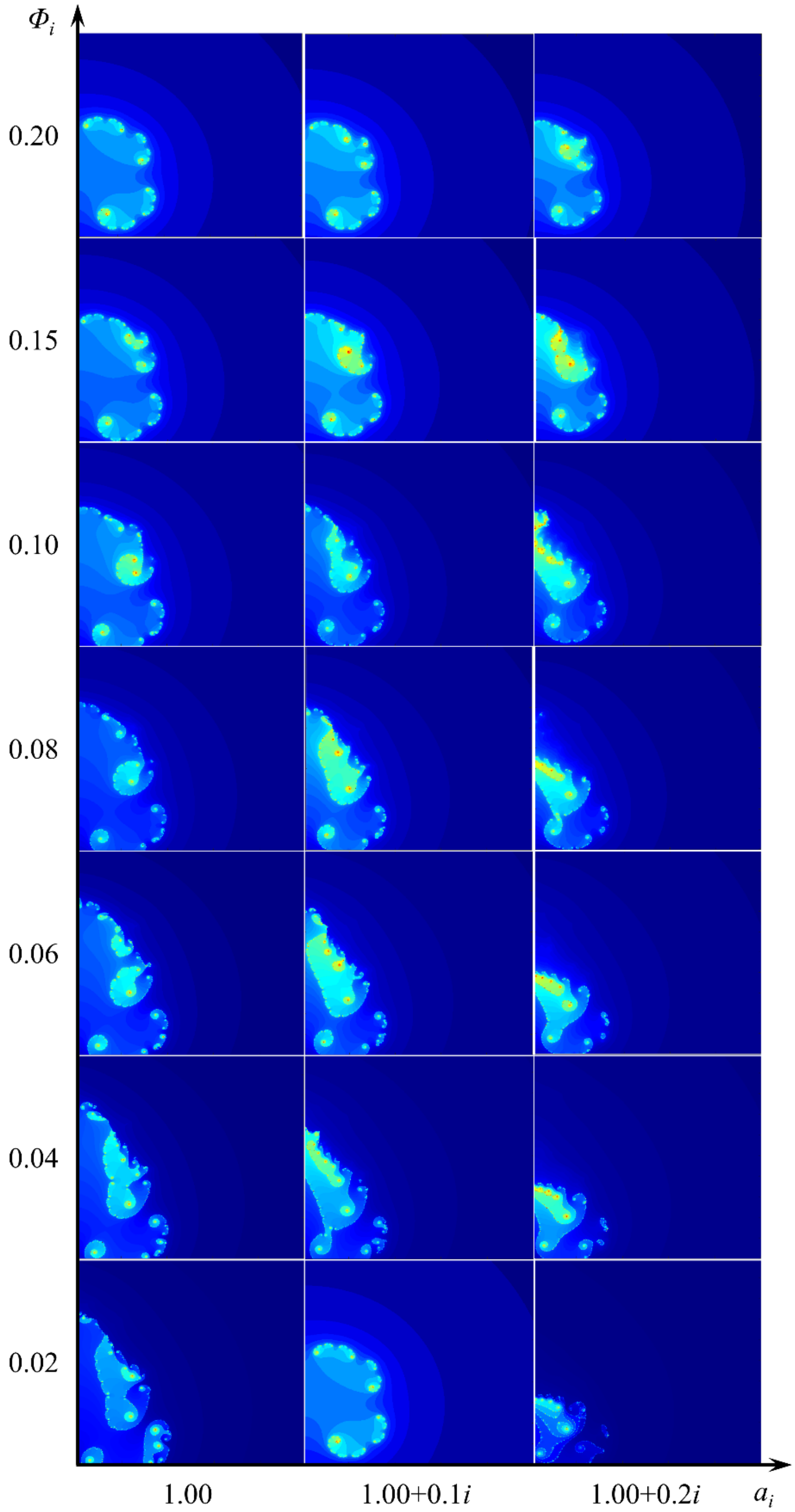

Fig. S38. Phase portraits of the simplified NS dynamical system with different control parameters for neuron 3. The horizontal and vertical coordinates correspond respectively to the real part and the imaginary part of the variable z. From blue to yellow and then to red, the number of iterations increased successively. The range of the coordinates of each phase portrait are all [0 5 ms].

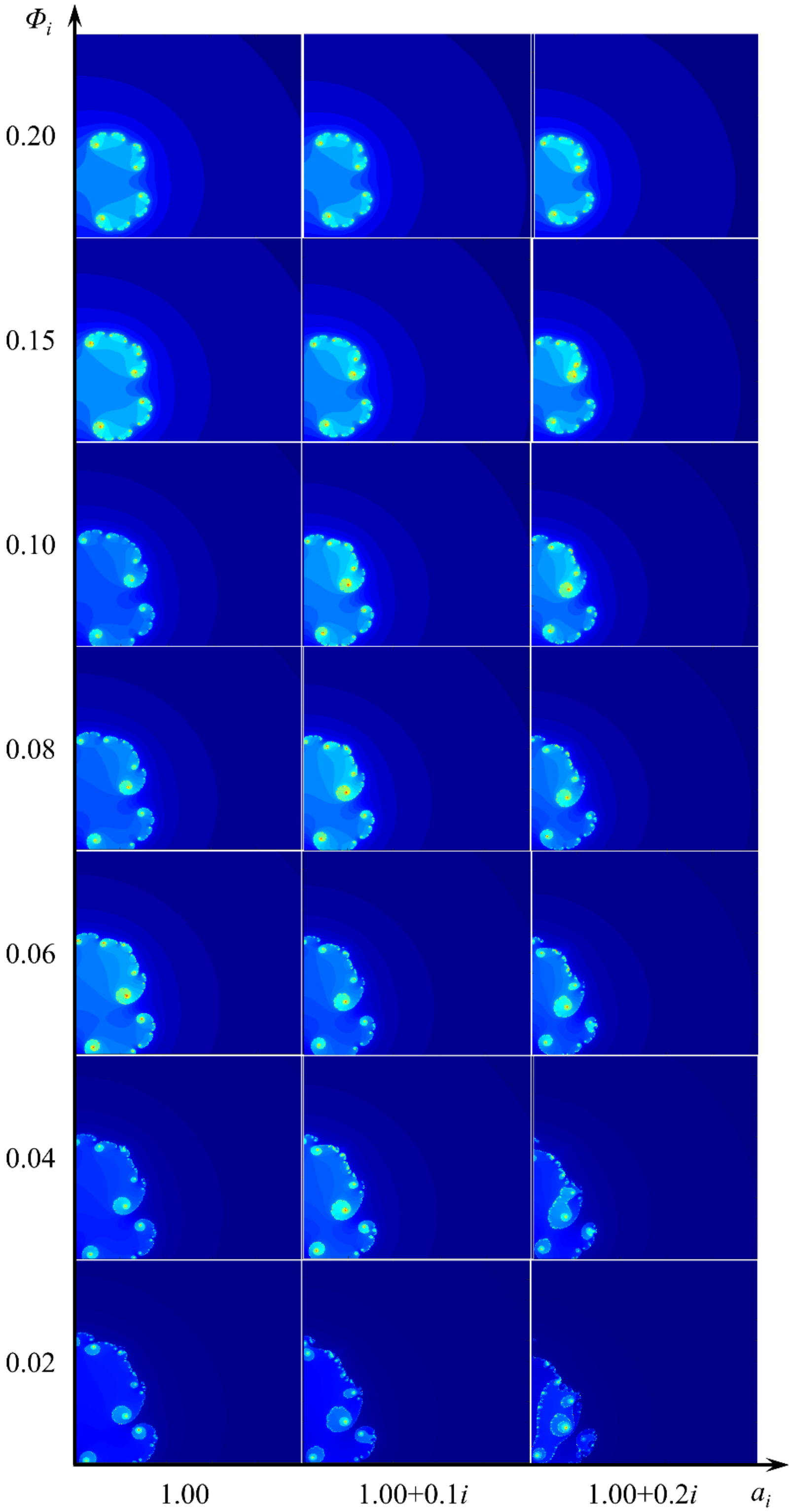

Fig. S39. Phase portraits of the simplified NS dynamical system with different control parameters for neuron 4. The horizontal and vertical coordinates correspond respectively to the real part and the imaginary part of the variable z. From blue to yellow and then to red, the number of iterations increased successively. The range of the coordinates of each phase portrait are all [0 5 ms].

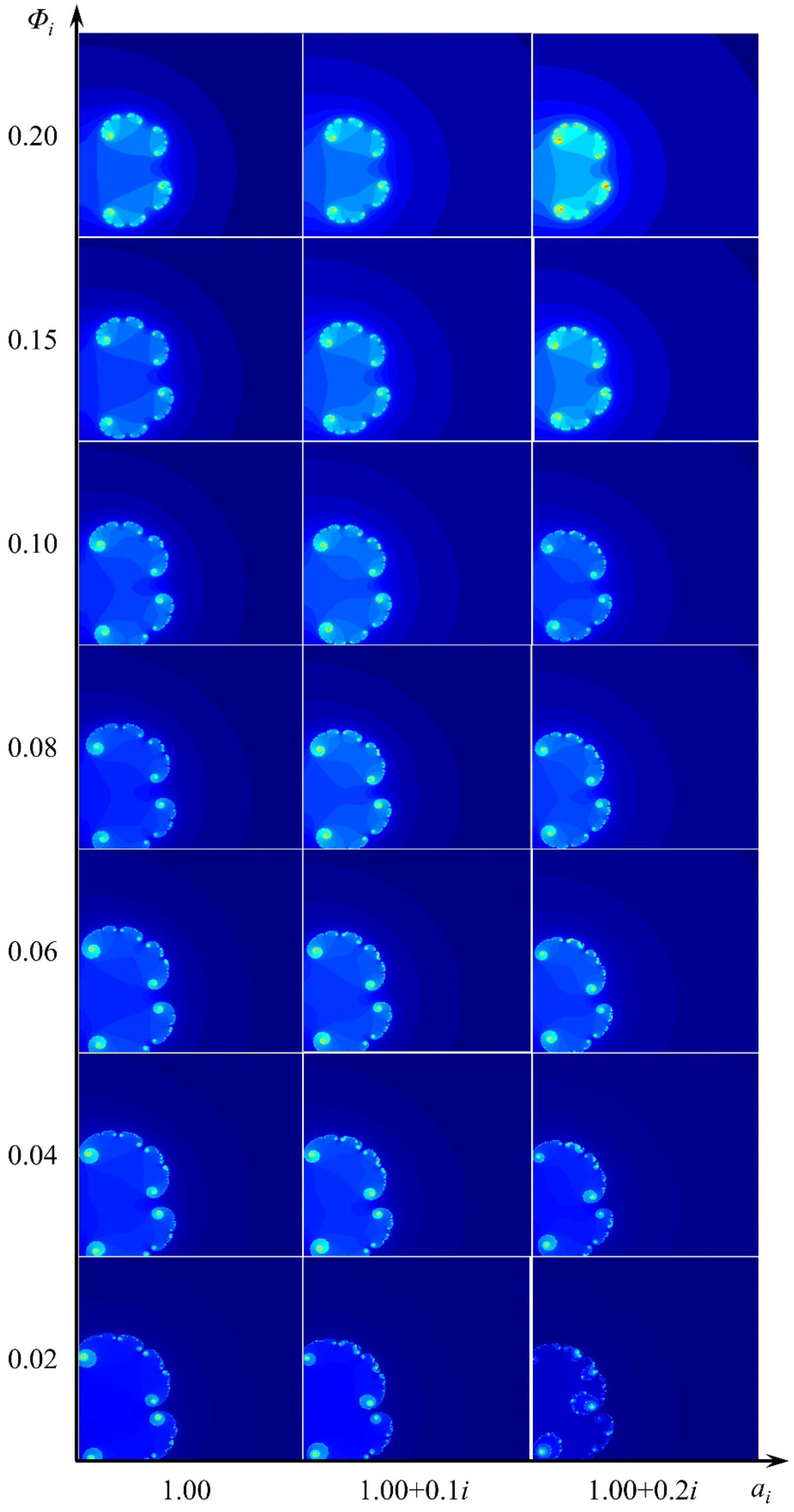

Fig. S40. Phase portraits of the simplified NS dynamical system with different control parameters for neuron 5. The horizontal and vertical coordinates correspond respectively to the real part and the imaginary part of the variable z. From blue to yellow and then to red, the number of iterations increased successively. The range of the coordinates of each phase portrait are all [0 5 ms].

Note 8 Evaluation of the storage capacity for the neural sphere (NS)

The storage capacity of the NS can be quantified to compare with other artificial devices. In addition to the widely used binary systems, the emerging two-dimensional material devices can generate many distinguishable states through regulation. The latest achievement of our team can regulate two-dimensional materials to generate up to thousands of states. Therefore, it is included in the comparison with NS here.

In addition to the various parameters of the NS, the storage capacity of the NS is affected by the temporal resolution t_{min}. In the ideal mathematical limit, the temporal resolution can be infinitesimal. In this case, NS can store an infinite amount of information. In the physical limit, the smallest temporal resolution is Planck time, 5.39×10^{-44} s. Although the exact limits of biological temporal resolution remain unknown, the temporal resolution of neurons can be roughly estimated based on the timescale of ion transport in neuronal ion channels and may potentially reach values on the order of attoseconds to picoseconds. These results are presented in Fig. S41. The figure shows that the self-information of NS is also much higher than that of the artificial device by about 1 to 2 orders of magnitude when the temporal resolution is between attoseconds and picoseconds.

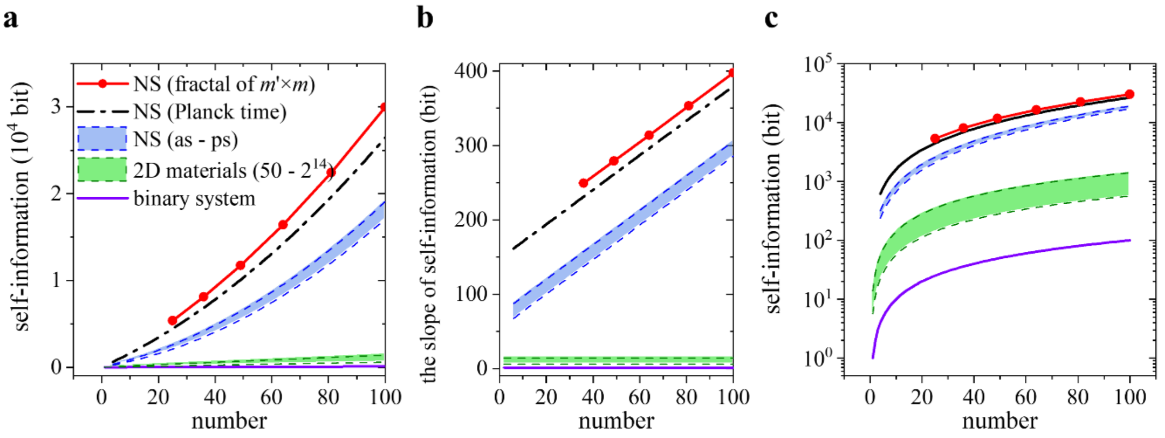

Fig. S41. Evaluation of storage capacity for NS. (a) Curves of the self-information of different systems as a function of the number of neurons or devices. The red line represents six **NS**s with the fractal of $m' \times m$ (m = 5, 6, …, 10). The black line represents the NS with a Planck time as its temporal resolution. The blue area represents the temporal resolution of the NS from one attosecond to one picosecond. The green area represents a system of some neuromorphic two-dimension material devices, with a single device having 50 to 2^{14} distinguishable states [22-24]. The purple line represents the binary system. (b) The slopes of the self-information curves in a. (c) The logarithmic coordinate of the self-information curves in a.

The storage capacity of the human brain can be simply estimated via NS. Assuming that the human brain with 86 billion neurons, each neuron has 5000 synapses. The connection structure of neurons in the human brain is a "small-world" network. The time resolution is Planck time.

The first is the conservative estimate. In this case, the brain is simply sparsely linearly connected by a large number of small-scale ensembles. Therefore, the self-information of the brain is only a linear accumulation of the self-information of small-scale ensembles. Directly applying Equation (53), the self-information of the brain can be estimated to be about 8.24 PB.

The second is the aggressive estimation. In this case, the neural network in the brain is a complex fractal structure as shown in box IV of Fig. S3 and Fig. 1d. According to the main text and Note 5, the electrophysiological activities of neurons in large-scale fractal NS are the arrangement of potential waveforms of small-scale NSs. 86 billion neurons correspond to a four-layer fractal for a small-scale ensemble of 5 neurons, i.e., fractal of 5'''×5''×5'×5. After three recurrences through Equations (53) and (56), the self-information of the brain can be estimated to be about 7.48 EB (i.e., 7.48×10^6 TB).

Besides, the information compression ratio of NS can be simply estimated at a low temporal resolution. When $t_{min} = 1\times10^{-9}$, the self-information of the initial conditions for four neurons is 3.74×10^3 bit according to Equation (59) and the self-information of the NS of the four neurons is 198 bit. Therefore, the information compression ratio is $1 - (198 / 3.74\times10^3) = 94.7\%$. In smaller t_{min} and NS with more neurons, the information compression ratio will be closer to 100%. It indicates that the brain has a very powerful ability of information compression and feature extraction.

Note 9 Evaluation of the computational power for the neural sphere (NS)

The computational power of silicon-based devices is generally measured by floating point operations per second (FLOPS) [25]. For CPU, GPU and other high performance chips, there are a set of well-established methods to test their computational power. According to the Table S5, the data of the latest generation high performance CPU and GPU and the top 10 supercomputer from various institutions or manufacturers are presented in Fig. 4c.

The computational power for floating-point operation of NS can be estimated by two equivalent methods according to Methods 6. In NSs with random action potential activity, Equation (60) is used for estimation. For the entire human brain, $FLOP_{NS} \in [10^3, 10^4]$ and f_{max} = 1/ absolutely refractory period ∈ [1000, 2000]. Therefore, the computational power of the human brain modeled by NS is estimated to range between 86 PFLOPS and 1.72 EFLOPS. Moreover, in this equivalent procedure, the precision of one floating-point operation is $\log_2(\tau_m / t_{min}) \in$ [34 bit, 138 bit]. In NSs with random action potential activity, Equation (61) is used for estimation. For the entire human brain, I = 7.5 EB, μ = 64 bit and T = 50 ms. Therefore, the computational power of the human brain modeled by NS is estimated to be 6.24 EFLOPS.

Type	Model number	Computational power	Design power	Data sources
CPU	Intel® Xeon® 6745P Processor	4.40 TFLOPS	300 W	https://www.intel.com/content/www/us/en/ark.html#@Processors
CPU	AMD EPYC™ 9965	6.91 TFLOPS	500 W	https://www.amd.com/en/products/processors/server/epyc/9005-series.html
GPU	AMD Radeon™ PRO W7900 Dual Slot	61.3 TFLOPS	295 W	https://www.amd.com/en/products/graphics/workstations.html
GPU	GeForce RTX 4090	82.5 TFLOPS	450 W	https://www.nvidia.com/en-us/geforce/graphics-cards/40-series/rtx-4090/
Super-computer	El Capitan	2,746.38 PFLOPS	29,581 kW	https://www.top500.org/lists/top500/2024/11/
Super-computer	Frontier	2,055.72 PFLOPS	24,607 kW	https://www.top500.org/lists/top500/2024/11/

Super-computer	Aurora	1,980.01 PFLOPS	38,698 kW	https://www.top500.org/lists/top500/2024/11/
Super-computer	Eagle	846.84 PFLOPS	/	https://www.top500.org/lists/top500/2024/11/
Super-computer	HPC6	606.97 PFLOPS	8,461 kW	https://www.top500.org/lists/top500/2024/11/
Super-computer	Supercomputer Fugaku	537.21 PFLOPS	29,899 kW	https://www.top500.org/lists/top500/2024/11/
Super-computer	Alps	574.84 PFLOPS	7,124 kW	https://www.top500.org/lists/top500/2024/11/
Super-computer	LUMI	531.51 PFLOPS	7,107 kW	https://www.top500.org/lists/top500/2024/11/
Super-computer	Leonardo	306.31 PFLOPS	7,494 kW	https://www.top500.org/lists/top500/2024/11/
Super-computer	Tuolumne	288.88 PFLOPS	3,387 kW	https://www.top500.org/lists/top500/2024/11/

Table S5. The computational power and design power data of the latest generation high performance CPU and GPU and the top 10 supercomputer from various institutions or manufacturers.

Note 10 Neural sphere (NS) can explain the unique functions of the brain

By integrating memories from these waveforms to accumulate experience, the NS provides an information handling dynamics for understanding the brain's unique capacities to generate reflexes, cognition, and creativity.

First, the NS exhibits an intrinsic ability to associate heterogeneous inputs. The same periodic potential waveform can store a large number of different inputs. This associative mechanism underlies cognitive phenomena such as associative memory, analogical reasoning, and discontinuous or "leaping" thinking, enabling the brain to flexibly connect disparate experiences and abstract relationships.

Second, the NS possesses a multi-storage architecture. Within a single memory NS, a large ensemble of distinct periodic potential waveforms can coexist, allowing multiple, independent information groups to be stored concurrently. Such an organization significantly enhances cross-interference resistance, ensuring the stability and integrity of memory retrieval even under overlapping neural representations.

Third, to support high-precision operations under low power consumption, the NS implements an offline storage mechanism. Once information storage or retrieval is completed, inhibitory neurons are activated to suppress the activity of the memory NS, thereby minimizing redundant neural activity and optimizing the energy efficiency of neural computation.

Fourth, coupling among multiple memory NSs enables reprocessing and reorganization of stored information. Through this coupling, the NS achieves an intrinsic integration of storage and computation—realizing a computing-in-memory paradigm that allows adaptive and context-dependent information processing without the need for distinct memory and computation modules.

Finally, the NS is capable of compressing high-dimensional initial conditions into low-dimensional feature representations. By selectively extracting and preserving essential informational components while discarding redundancies, the system maximizes memory efficiency and abstraction capacity. This property provides a plausible neural basis for higher-order cognitive functions such as generalization, concept formation, and creative synthesis.

References

1. Ascoli GA, Donohue DE, Halavi M. NeuroMorpho.Org: A Central Resource for Neuronal Morphologies. *J Neurosci*. 2007; **27**(35): 9247–51.
2. Rall W. Branching dendritic trees and motoneuron membrane resistivity. *Exp Neurol*. 1959; **1**: 491–527. doi: 10.1016/0014-4886(59)90046-9
3. Hodgkin AL, Huxley AF. Currents carried by sodium and potassium ions through the membrane of the giant axon of loligo. *J Physiol*. 1952; **116**: 449–72.
4. Hodgkin AL, Huxley AF. The components of membrane conductance in the giant axon of loligo. *J Physiol*. 1952; **116**: 473–96.
5. Hodgkin AL, Huxley AF. The dual effect of membrane potential on sodium conductance in the giant axon of loligo. *J Physiol*. 1952; **116**: 497–506.
6. Hodgkin AL, Huxley AF. A quantitative description of membrane current and its application to conduction and excitation in nerve. *J Physiol*. 1952; **116**: 500–44.
7. Hodgkin AL, Huxley AF, Katz B. Measurement of current-voltage relations in the membrane of the giant axon of loligo. *J Physiol*. 1952; **116**: 449–72.
8. Sporns O, Kötter R. Motifs in brain networks. *PLOS Biol*. 2004; **2**: e369.
9. Gonzalez, Alvaro. Measurement of Areas on a Sphere Using Fibonacci and Latitude-Longitude Lattices. *Math Geosci*. 2010; **42**(1): 49–64.
10. Ding SL, Royall JJ, Sunkin SM. Comprehensive cellular-resolution atlas of the adult human brain. *J Comp Neurol*. 2016; **524**(16): 3127–481.
11. Shannon CE. A mathematical theory of communication. *The Bell System Technical Journal*. 1948; **27**(3): 379–423. doi: 10.1002/j.1538-7305.1948.tb01338.x
12. Bérut A, Arakelyan A, Petrosyan A *et al.* Experimental verification of Landauer's principle linking information and thermodynamics. *Nature*. 2012; **483**: 187–9.
13. Jun Y, Gavrilov M, Bechhoefer J. High-Precision Test of Landauer's Principle in a Feedback Trap. *Phys Rev Lett*. 2014; **113**: 190601.
14. Lennie P. The Cost of Cortical Computation. *Curr Biol*. 2003; **13**(6): 493–7.
15. Markram H, Muller E, Ramaswamy S *et al.* Reconstruction and Simulation of Neocortical Microcircuitry. *Cell*. 2015; **163**(2): 456-92.
16. Mombaerts P, Wang F, Dulac C *et al.* Visualizing an Olfactory Sensory Map. *Cell*. 1996; **87**(4): 675-86.
17. Cook SJ, Jarrell TA, Brittin CA *et al.* Whole-animal connectomes of both Caenorhabditis elegans sexes. *Nature*. 2019; **571**: 63–71.
18. Yao Z, Velthoven CTJv, Kunst M *et al.* A high-resolution transcriptomic and spatial atlas of cell types in the whole mouse brain. *Nature*. 2023; **624**: 317–32.
19. Park KI, Park M. Parameters of a Stochastic Process. *Fundamentals of probability and stochastic processes with applications to communications*. Cham: Springer; 2018. 165–8.
20. Buzsáki G, Draguhn A. Neuronal Oscillations in Cortical Networks. *Science*. 2004; **304**: 1926–9.
21. Kosinski RJ. A literature review on reaction time. *Clemson Univ*. 2008; **10**(1): 337–44.
22. Zheng Z, Ma Q, Bi Z *et al.* Unconventional ferroelectricity in moiré heterostructures. *Nature*. 2020; **588**: 71–6.
23. Yasuda XWK, Watanabe K, Taniguchi T *et al.* Stacking-engineered ferroelectricity in bilayer boron nitride. *Science*. 2021; **372**: 1458–62.
24. Sharma D, Rath SP, Kundu B *et al.* Linear symmetric self-selecting 14-bit kinetic molecular memristors. *Nature*. 2024; **633**: 560–6.

25. J KD. *Parameters of a Stochastic Process*. US Department of Commerce: National Bureau of Standards, 1974.
